# Generic multidimensional economic environmental operation of power systems using equilibrium optimization algorithm

**DOI:** 10.1038/s41598-025-00696-x

**Published:** 2025-05-16

**Authors:** Mohamed T. Mouwafi, Adel A. Abou El-Ela, Amany A. El-Hamoly, Ragab A. El-Sehiemy

**Affiliations:** 1 Electrical Engineering Department, Faculty of Engineering, Menoufia University, Shebin El-Kom, 32511 Egypt; 2 Electrical Engineering Department, Faculty of Engineering, Kafrelsheikh University, Kafrelsheikh, 33511 Egypt; 3 Sustainability Competence Centre, Szecheny Istvan University, Egyetem square 1, gyor, H-9026 Hungary

**Keywords:** Economic emission load dispatch, Emission, Equilibrium optimization algorithm, Generation cost function, Valve-points effects, Energy science and technology, Engineering

## Abstract

The economic emission load dispatch (EELD) problem is one of the main challenges to power system operators due to the complexity of the interconnected power systems and the non-linear characteristics of the objective functions (OFs). Therefore, the EELD problem has attracted significant attention in the electric power system because it has important objectives. Thus, this paper proposes the equilibrium optimization algorithm (EOA) to solve the EELD problem in electrical power systems by minimizing the total fuel cost and emissions, considering system and operational constraints. The OFs are optimized with and without considering valve point effects (VPE) and transmission system loss. The multi-OF, which aims to optimize these objectives simultaneously, is considered. In the proposed EOA, agents are particles and concentrations that express the solution and position, respectively. The proposed EOA is evaluated and tested on different-sized standard test systems having 10, 20, 40, and 80 generation units through several case studies. The numerical results obtained by the proposed EOA are compared with other optimization techniques such as grey wolf optimization, particle swarm optimization (PSO), differential evolution algorithm, and other optimization techniques in the literature. To show the reliability of the proposed algorithm for solving the considered OFs on a large-scale power system with and without considering different practical constraints such as VPE, ramp-rate limits (RRL), and prohibited operating zones (POZs) of generating units, the proposed EOA is evaluated and tested on the 140-unit test system. Also, the proposed multi-objective EOA (MOEOA) successfully acquires the Pareto optimal front to find the best compromise solution between the considered OFs. Also, the statistical analysis and the Wilcoxon signed rank test between the EOA and other optimization techniques for solving the EELD problem are performed. From numerical results, the total fuel cost obtained without considering VPE using the proposed EOA is reduced by 0.1414%, 0.1295%, 0.6864%, 5.8441% than the results of PSO, with maximum savings of 150 $/hr, 78 $/hr, 820 $/hr, and 14,730 $/hr for 10, 20, 40, and 80 units, respectively. The total fuel cost considering VPE is reduced by 0.0753%, 0.2536%, 2.8891%, and 3.6186% than the base case with maximum savings of 80 $/hr, 158 $/hr, 3610 $/hr, 9230 $/hr for 10, 20, 40, and 80 units, respectively. The total emission is reduced by 1.7483%, 12.8673%, and 7.5948% from the base case for 10, 40, and 80 units, respectively. For the 140-unit test system, the total fuel cost without and with considering VPE, RRL, and POZs is reduced by 6.4203% and 7.2394%, than the results of PSO with maximum savings of 107,200 $/hr and 126,400 $/hr. The total emission is reduced by 2.5688% from the base case. The comparative studies show the superiority of the EOA for the economic/environmental operation of the power system by solving the EELD problem with more accuracy and efficiency, especially as the system size increases.

## Introduction

### Motivation

The economic dispatch (ED) problem is a subroutine of the unit commitment problem, which aims to find the optimal real power outputs of generation units such that the entire load may be supplied most economically. To reduce the total production costs, we need to satisfy the constraints of total load demand as well as respect the limits of resource capacity^1^. The dispatch problems become more complex when the system and operational constraints are considered, such as network transmission losses and valve-point loading effects. Therefore, the cost function must be represented by a quadratic convex/linear function to become easy and can be solved^2^. There is a rippling effect on the curve of the unit’s power cost when a steam valve starts to open. The term “optimization” can be defined as the procedure of detection that provides the minimum or maximum value of an objective function (OF)^3^. Therefore, the economic emission load dispatch (EELD) problem can be formulated as a constrained optimization problem.

### Literature survey

Initially, conventional optimization methods were used mainly as an optimization tool for solving the ED problem, such as linear programming techniques^3^. In addition, there are methods based on classical calculus or deterministic numerical methods are developed to solve convex ED problems, such as the Lagrangian multipliers (LM) method^4^, base point and participation factors method^5^, lambda-iteration method^6^, interior point method^7^, gradient method^8^, Newton method^9^, linear programming (LP)^10–12^and nonlinear programming (NLP)^13,14^. However, these methods suffer from several drawbacks, such as the convergence to local optima instead of global solution and the theoretical assumptions such as convexity, differentiability, and continuity. Due to the nature of control variables, objectives, and constraints of the ED problem, conventional optimization techniques may not be suitable for solving the ED problem.

Recently, the drawbacks of conventional methods in solving ED problems have been treated successfully by meta-heuristic optimization techniques due to their simplicity, flexibility to solve any optimization problem, capability to find global-optimal solutions, and independence from the nature of the problem since they use a stochastic approach for finding optimal solutions without being concerned about the nonlinearity types of the problem’s search space and its constraints^15^. Therefore, these techniques have gained more attention for solving different optimization problems. Many standard meta-heuristic optimization techniques have been applied to solve the ED problem. In^16^, a numerical algorithm based on a Python computer program was used to solve the environmental/economic load dispatch (EELD) problem considering emissions constraints, which considers the emissions trading system’s effect on electricity generation cost. However, only one small standard test system size is considered. In^17^, The search and rescue optimization algorithm (SAR) was applied to solve the combined emission and economic dispatch (CEED) and economic load dispatch (ELD). However, only two small standard test systems are considered. In^18^, a dual-population adaptive differential evolution (DPADE) algorithm was utilized to solve the complex large-scale and non-convex ED problems considering both multi-fuel options (MFO) and valve-point effects (VPE). Firstly, a dual-population framework was employed to improve the search space efficiency. Then, an adaptive technology was adopted to adjust two important control parameters and avoid inappropriate parameters. However, the emission effects are not considered. In^19^, a hybrid algorithm based on a combination of a modified genetic algorithm (GS) and an improved particle swarm optimization (PSO) was used to solve the CEED problem considering practical operational constraints such as VPE, MFO, ramp-rate limits (RRL), prohibited operating zones (POZs) of generating units, and transmission lines losses. In^20^, an improved PSO integrated with a simplex search method (MPSO_SSM) to perform the hybrid operation using stochastic and deterministic methods was applied to solve the economic-emission power dispatch (EEPD) multi-objective problems considering the VPE and multifuel dispatch. In^21^, a probability distribution arithmetic optimization algorithm (AOA) based on a variable order penalty strategy was utilized to solve the CEED problem considering five probability distribution functions to enhance the searching ability, improve the convergence speed, and enhance the ability to jump out of the local optimal. However, only one small standard test system with 6 units is considered. In^22^, the membrane search algorithm (MSA) was used to solve the combined heat and power economic emission dispatch (CHPEED) problem by allocating heat and electrical power loads to various types of units to minimize the total cost and emissions of thermal generation units, while satisfying system constraints.

In^23^, a comparison between the flower pollination algorithm (FPA) and the bat algorithm (BA) was presented to solve the ED problem with and without emission effects in the power system considering the operational constraints of the generators. In^24^, the quasi-oppositional search-based political optimizer (QOPO) was used to solve a single and bi-objective CEED problem by minimizing total fuel costs and emissions considering different constraints such as the VPE and generator limits were achieved. However, large-scale power systems are not considered. In^25^, a comparison between the Osprey optimization algorithm (OOA) and other optimization algorithms was introduced to solve the ED problem with and without emission effects. However, the optimal values of generations’ output powers are not mentioned in the results. In^26^, the non‑dominated sorting multi‑objective PSO with local best was used to solve the CEED problem in power systems, while a Markov chain state jumping technique was employed to control the Pareto‑optimal set size. In^27^, an oppositional driven crisscross gravitational search approach (OCcGSA) was applied to solve the ED problem by minimizing the total operating cost considering operational constraints such as VPE, RRL, and POZs of generating units. However, the emission effects are not considered. In^28^, an updated differential evolution (UDE) algorithm based on a new mutation strategy was used to solve the ED problem considering RRL, POZs, and transmission line capacity. However, only small standard test systems are considered. In^29^, a novel based on constraints handling method was employed to solve the ED problem with VPE, consisting of the power repair strategy to modify the generator output power, and the adaptive penalty function to change according to the fitness value of the OF. In^30^, a semi-definite programming approach was used to solve the ED problem by minimizing the total fuel cost in two areas of an electrical power system, where tie transmission line capacity was considered a constraint. In^31^, the social small group optimization (SSGO) algorithm was applied to solve the ED problem considering VPE, MFO, POZs, and transmission line losses. In^32,33^, a reinforcement learning-based DE algorithm was developed to solve the CEED problem considering the quadratic function in^32^, and both quadratic function and cubic criterion function in^33^.

In^34^, the numerical polynomial homotropy continuation (NPHC) method was applied to solve the CEED problem considering transmission line losses. However, different constraints such as VPE, RRL, and POZs are not considered. In^35^, the hybrid firefly algorithm (FA) and genetic algorithm (GA) were used to solve the EELD problem considering nonlinear constraints such as VPE, POZs, and RRL. The hybrid algorithm started with a potential answer searched around it based on a creative heuristic and then moved on to another potential answer. However, only small standard test systems are considered. In^36^, the DE based on a comprehensive learning strategy (CLS) was applied to solve large-scale power system multi-area ED considering the VPE. Three improved components, including a global guided mutation strategy based on CLS, a time-varying increasing crossover rate, and a crossover strategy based on CLS to address DE’s shortcomings were incorporated to enhance the performance of comprehensive learning DE (CLDE). However, the emission effects are not considered. In^37^, the AOA with three-dimensional chaotic mapping in a spherical coordinate system was used to solve the CEED problem. Five three-dimensional chaotic mappings in a spherical coordinate system were employed to improve the algorithms’ ability to balance exploration and exploitation and avoid falling into the local optimums. In^38^, the optimization without penalty-based optimization by morphological filter algorithm (OWP-based OMF) was applied to solve the CEED problem considering the equality and inequality constraints such as VPE and transmission line loss. However, large-scale power systems are not considered. In^39^, a hybrid multi-objective algorithm based on Harris Hawks optimization (HHO) and DE was developed to solve the EELD problem with VPE. The concept of Pareto domination was integrated into HHO to deal with the EELD problem with two conflicting objectives. In^40^, the BBO algorithm was utilized to solve the ED problem considering equality and inequality constraints such as transmission line losses, RRL, and POZs. However, the emission effects and large-scale power systems are not considered. In^41^, a Chi-square mutated quantum PSO (QPSO-Chi2) was applied to solve the CEED problem with and without VPE considering transmission system losses.

In^42^, a multi-objective learning backtracking search algorithm (MOLBSA) was used to solve the EELD problem considering a leader-choosing strategy and a leader-guiding strategy as two novel learning strategies to improve the uniformity and diversity of obtained Pareto front. However, only small standard test systems are considered. In^43^, an innovative hybrid algorithm based on novel DE and PSO was applied to solve the CEED problem considering different constraints such as VPE, RRL, and POZs. The novel DE introduced an improved mutation and crossover approach, while the novel PSO introduced a new acceleration coefficient, inertia weight, and position improvement equation. However, large-scale power systems are not considered. In^44^, a data-driven look-ahead economic dispatch model with the full consideration of *N*−1outage contingency based on reinforcement learning and a deep deterministic policy gradient (DDPG) algorithm was employed to solve the ED problem by minimizing the total fuel cost. However, the emission effects are not considered. In^45^, a multi-layer distributed multi-objective consensus algorithm was used to solve the ED problem by determining the optimal power generation of each area of each layer through the network topology and then calculating the power of each unit in each area, in parallel according to the calculated optimal power generation. In^46^, the turbulent flow of water optimization (TFWO) algorithm was applied to solve the ED problem with transmission line losses, and the CEED problem with and without VPE. In^47^, the integration of the traditional sand cat optimization algorithm (SCOA) with the Levy flight (LF) concept was used to solve the CEED problem by minimizing fuel costs and the emission of generation units, while the equality constraints of the CEED problem were transformed into inequality constraints. However, large-scale power systems are not considered. In^48^, an enhanced moth-flame optimization (EMFO) algorithm was utilized to solve the non-convex ED problem with VPE and emissions by minimizing total fuel cost and emission. In^49^, the parallel hurricane optimization algorithm (PHOA) was used to solve the EELD problem in modern power systems by minimizing the total fuel cost and emission with and without considering the VPE.

In^50^, the BSA was used to solve the ED problem, considering the VPE in the generator cost function and the transmission network losses. However, the emission effects are not considered. In^51^, a data-driven surrogate-assisted approach was used to solve the multi-area CEED (MACEED) problem. First, a feature engineering-based support vector regression surrogate model was utilized to replace the traditional OFs in high-dimensional MACEED problems. Then, knowledge distillation was used as a freezing and fine-tuning mechanism for the improved support vector regression surrogate models. Finally, a non-dominated sorting GA was applied to obtain feasible solutions to the high-dimensional MACEED problem. In^52^, the colonial competitive DE (CCDE) that employed a different DE algorithm based on mathematical modeling of socio-political evolution was used to solve the ED problem considering different constraints and operational limitations such as VPE, RRL, and POZs. In^53,54^, a novel hybrid algorithm that combined the DEA and PSO was applied to solve the ED problem considering different constraints such as VPE, RRL, POZs, and spinning reserve. However, the emission effects are not considered. In^55^, a comprehensive review of the ED problem was introduced based on the mathematical formulation and the examination of commonly used problem formulation techniques, including single and multi-objective optimization. In^56,57^, a comprehensive review of the CEED problem was presented based on the comparative analysis of optimization approaches in^56^, and models, categorizing them according to the control of atmospheric pollutants in^57^. In^58–63^, different optimization techniques were used to solve the ED problem in power systems incorporating renewable energy sources (RES) in^58,59^, while the CEED problem was solved considering the integration of RES and plug‑in electrical vehicle (PEVs) in^60–63^.

Recently, Afshin Faramarzi et al.^64^. proposed the original version of the equilibrium optimization algorithm (EOA) as one of the new meta-heuristic optimization algorithms. The equilibrium optimizer (EO) is inspired by control volume mass balance to estimate dynamic and equilibrium states. In EO, search agents randomly update their concentration (position) concerning some talented particles called equilibrium candidates to reach an equilibrium state as optimal results. The EOA was applied to solve different optimization problems such as image segmentation^65^, optimal estimation of Schottky diode parameters^66^, optimal allocation of batteries in distribution systems^67,68^, network reconfiguration, distributed generation (DG) allocation^69^, and optimal power flow (OPF)^70^.

From the previous literature review, it can be concluded that,

- Different constraints such as VPE, RRL, and POZs were considered in a few papers. Therefore, this paper considers these constraints when solving the EELD problem.
- The transmission line loss was considered in a few papers. Therefore, this paper proposes the EOA to solve the EELD problem with and without considering transmission line losses.
- The application of optimization techniques on large-scale test systems was introduced in a few papers. Therefore, the proposed algorithm is evaluated and tested on small, and large-scale standard test systems.
- The statistical analysis was considered in a few papers. Therefore, this paper presents statistical analysis to show the superiority of the proposed algorithm for finding optimal solutions.
- The optimal values of control variables reported in some papers lead to infeasible solutions due to violations in some constraints. Therefore, this paper presents accurate results that lead to feasible solutions that achieve all constraints.

### Paper contribution

This paper presents a proposed methodology based on the EOA to solve the EELD problem in electrical power systems. The main contributions of this paper are enumerated as follows:

- Applying the EOA as one of the new meta-heuristic optimization techniques to solve the EELD problem considering single and multi-objective functions.
- Two OFs are minimized individually, which are the total fuel cost minimization with and without VPE, and total emission minimization.
- Applying the multi-objective EOA (MOEOA) to minimize the total fuel cost and total emission simultaneously.
- Applying the proposed EOA on different size standard test systems through various case studies.
- Applying the proposed EOA with Pareto front on the large-scale power system to solve the EELD problem with, and without considering different practical constraints such as VPE, RRL, and POZs of generating units.
- Proving the superiority of the proposed methodology for solving the EELD problem by comparing the optimal results with other techniques such as GWO, PSO, DEA, and other optimization methods in the literature.
- A comparative study based on statistical analysis and the Wilcoxon signed rank test is carried out between the proposed EOA and other techniques to show the effectiveness of the proposed EOA for solving the EELD problem.

### Paper organization

This paper is organized as follows. The next section presents the problem formulation of the EELD problem. After the problem is formulated, the followed two sections present the proposed EOA followed by several applications for solving the EELD problem. The last section presents the conclusion of this paper.

## Problem formulation

Two OFs are considered in this paper for solving the EELD problem. The first OF aims to minimize the non-linear generation cost function with and without VPE, while the second OF aims to reduce the total emission.

### Objective functions

The generation cost function can be modeled as a polynomial function, where it is generally described by a quadratic function for each generator. Therefore, the total generation production costs can be formulated as^49^:

1 $${f_1}=Min\left( {\sum\limits_{{i=1}}^{{{N_G}}} {{a_i}+{b_i}{P_{Gi}}+{c_i}P_{{Gi}}^{2}} } \right)$$

where, *P*_*Gi*_ is the real power generation for the generation unit *i*. *a*_*i*_, *b*_*i*_, and *c*_*i*_ are the cost function coefficients of *i*^*th*^ generator. *N*_*G*_is the total number of generation buses. Practically, generation units have multi-valve steam turbines. Each steam valve can be controlled to change the power production. Therefore, the fuel cost function considering the non-smooth VPE can be expressed as^48^:

2 $${f_1}=Min\left[ {\sum\limits_{{i=1}}^{{{N_G}}} {\left( {{a_i}+{b_i}{P_{Gi}}+{c_i}P_{{Gi}}^{2}} \right)+\left| {{d_i} \times \sin \left[ {{e_i}\left( {{P_{Gi}} - P_{{Gi}}^{{\hbox{min} }}} \right)} \right]} \right|} } \right]$$

where, *d*_*i*_ and *e*_*i*_ are the coefficients of the non-smooth operation of valves, and$P_{{Gi}}^{{\hbox{min} }}$is the minimum limit of power generation for the generation unit *i*.

The ecological emissions produced by fossil-fueled thermal units should be considered due to their effects on the environment. Therefore, the second OF aims to minimize the total emission by reducing atmospheric pollutants such as nitrogen and sulfur oxides. Hence, the total emission pollutants from thermal units can be formulated as^48^:

3 $${f_2}=Min\left[ {\sum\limits_{{i=1}}^{{{N_G}}} {\left( {{\alpha _i}+{\beta _i}{P_{Gi}}+{\gamma _i}P_{{Gi}}^{2}} \right)+\left| {{\zeta _i}\exp \left( {{\lambda _i}{P_{Gi}}} \right)} \right|} } \right]$$

where, *α*_*i*_, *β*_*i*_, *γ*_*i*_, *ζ*_*i*_ and *λ*_*i*_ are the emission coefficients of *i*^*th*^ generator.

The multi-OF can be performed by transforming different OFs into a single OF using weighting factors to make a balance between different objectives and avoid the dominance of one objective over another. Hence, it can be formulated as:

4 $$\begin{gathered} {F_t}=Min\left[ {{k_c}\,{F_1}+{k_E}\,{F_2}} \right] \hfill \\ \,\,\,\,\,\,=Min\left[ {{k_c}\,\left( {\frac{{{f_1}}}{{f_{1}^{{\hbox{max} }}}}} \right)+{k_E}\,\left( {\frac{{{f_2}}}{{f_{2}^{{\hbox{max} }}}}} \right)} \right] \hfill \\ \end{gathered}$$

where, *f*_1_ is the total fuel cost for each population (particle) in the optimization technique, and$f_{1}^{{\hbox{max} }}$is the maximum value of the total fuel cost among all populations (particles). Similarly, *f*_2_ is the total emission pollutants for each population (particle) in the optimization technique, and$f_{2}^{{\hbox{max} }}$is the maximum value of the total emission pollutants among all populations (particles), *k*_*c*_ and *k*_*E*_ are the weighting factors that are assumed to be 0.6 and 0.4, respectively. The value of the weighting factor shows the priority of the OF in solving the multi-OF.

### System constraints

The OFs in Eqs. (1)-(4) are subjected to the following equality and inequality constraints:

- *Power balance constraint*.

This constraint aims to check the balance between the total generated active power and the sum of the total load demand and the total system losses. Thus, this constraint can be defined as follows:

5 $$\sum\limits_{{i=1}}^{{{N_G}}} {{P_{Gi}}} =\sum\limits_{{j=1}}^{{{N_L}}} {{P_{Dj}}} +P_{{Loss}}^{{Total}}$$

where, *P*_*Dj*_ is the load demand at load bus *j*, $P_{{Loss}}^{{Total}}$is the total real power loss, and *N*_*L*_ is the total number of load buses. The total real power loss can be calculated based on the values of *B*-coefficients as follows^48^:

6 $$\begin{gathered} P_{{Loss}}^{{Total}}={\left[ {{P_G}} \right]^T}\left[ B \right]\left[ {{P_G}} \right]+{\left[ {{B_0}} \right]^T}\left[ {{P_G}} \right]+{B_{00}} \hfill \\ \,\,\,\,\,\,\,\,\,\,\,\,\,=\sum\limits_{{i=1}}^{{{N_G}}} {\sum\limits_{{j=1}}^{{{N_G}}} {{P_{Gi}}{B_{ij}}{P_{Gj}}} } +\sum\limits_{{i=1}}^{{{N_G}}} {{B_{0i}}{P_{Gi}}} +{B_{00}} \hfill \\ \end{gathered}$$

where, [*P*_*G*_] is the vector of all generator buses. [*B*], [*B*_0_], and *B*_00_ are the quadratic, linear, and constant parts of *B*-coefficients, respectively.

- *Power generation constraint*.

The active power supplied by each generating unit must be within their acceptable limits as follows:

7 $$P_{i}^{{\hbox{min} }} \leqslant {P_i} \leqslant P_{i}^{{\hbox{max} }}$$

where, $P_{i}^{{\hbox{min} }}$and $P_{i}^{{\hbox{max} }}$are the minimum and maximum limits of power generation from the generation unit *i* (MW), respectively.

- *Ramp rate limits*.

The power output of each generation unit increases or decreases over time according to the ramp rate limits to keep a suitable balance between power supply and demand and prevent undesirable effects in the power system. Therefore, the change in generation output power should be restricted by the ramp-up and ramp-down constraints, which can be written as follows:

8 $$\hbox{max} \left( {P_{i}^{{\hbox{min} }},P_{i}^{0} - D{R_i}} \right) \leqslant {P_i} \leqslant \hbox{min} \left( {P_{i}^{{\hbox{max} }},P_{i}^{0}+U{R_i}} \right)$$

where, *P*_*i*_ is the current real output power from the generation unit *i* (MW), *P*_*i*_^0^ is the previous real output power of the generation unit *i* (MW), *DR*_*i*_ and *UR*_*i*_ are the upper and lower ramp rate limits of the generation unit *i* (MW/period), respectively.

- *Prohibited operating zones constraint*.

Due to physical operation restrictions of some power generation plant components such as faults in power generation units or associated auxiliaries, these thermal generation units may have POZs between their minimum and maximum limits. Therefore, the fuel cost characteristics become discontinuous. To avoid the operation of generation units in the prohibited zones, the POZs constraint in Eq. (9) for such units should be considered.

9 $${P_i} \in \left\{ {\begin{array}{*{20}{c}} {P_{i}^{{\hbox{min} }} \leqslant {P_i} \leqslant P_{{i,1}}^{L}} \\ {P_{{i,k - 1}}^{U} \leqslant {P_i} \leqslant P_{{i,k}}^{L}} \\ {P_{{i,p{z_i}}}^{U} \leqslant {P_i} \leqslant {P_{i,{\rm max} }}} \end{array}} \right.\,;\,\,\,\,k=2,3,\,\cdots\cdots,p{z_i},\,\,\,\,i=1,2,\,\cdots\cdots,\,{n_{pz}}$$

where, $P_{{i,k}}^{L}$ and $P_{{i,k}}^{U}$ are the lower and upper limits of POZ of the generation unit *i* (MW), respectively, *pz*_*i*_ is the number of prohibited zones of the generation unit *i*, and *n*_*pz*_ is the number units which have POZs.

- *Power flow constraint*.

The power flow in each line (*PF*_*k*_) must be less than its maximum limit of power flow (*PF*_*k*_^*max*^) as:

10 $$P{F_k}<PF_{k}^{{\hbox{max} }}$$

## Proposed methodology based on eoa

### optimization algorithm

The EOA, which was first proposed in^64^, is one of the meta-heuristic optimization techniques that is inspired based on the physics laws. The mathematical model of EOA is illustrated based on the following three steps:

*Step 1: Initialization*.

Initially, a population matrix is created with random values, where each row refers to the particle that represents the concentration vector. The initial values of concentration vector ($~\overrightarrow {{X_i}}$) are generated randomly, as follows:

11 $$~\overrightarrow {{X_i}} ={X^{\hbox{min} }}+rand \times \left( {{X^{\hbox{max} }} - {X^{\hbox{min} }}} \right),\,\,\,\,\,\,~~i=1,2,3, \ldots \ldots ~,n~$$

where, *X*^*min*^ and *X*^*max*^ are the minimum and maximum limits of vector *x* in *d*-dimension, respectively.

*Step 2: Equilibrium pool and candidates* (${\overrightarrow X _{eq,pool}}$)

Each particle reaches the optimal solution by searching for the equilibrium state. Then, the best four particles found in the population are assigned as candidates, plus another one calculates the average value of the best four particles. After that, the equilibrium pool vector ($~{\overrightarrow X _{eq,pool}}$) is generated based on the five candidates of particles as follows^64^:

12 $$~{\overrightarrow X _{eq,pool}}=\left[ {{{\overrightarrow X }_{eq\left( 1 \right)}}+{{\overrightarrow X }_{eq\left( 2 \right)}}+{{\overrightarrow X }_{eq\left( 3 \right)}}+{{\overrightarrow X }_{eq\left( 4 \right)}}+{{\overrightarrow X }_{eq\left( {avg} \right)}}} \right]$$

where,$~{\overrightarrow X _{eq\left( {avg} \right)}}$ is the average value of candidates.

*Step 3: Updating the concentration*.

For each particle, the concentration is updated with random selection among candidates chosen with the same probability as follows^64^:

13 $$~{\overrightarrow X _{new}}={\overrightarrow X _{eq}}+\left( {\overrightarrow X - {{\overrightarrow X }_{eq}}} \right) \times \overrightarrow F +\frac{G}{{\overrightarrow \lambda \times V}} \times \left( {1 - \overrightarrow F } \right)$$

where, $\overrightarrow X$and $~{\overrightarrow X _{new}}$are the current and new concentration vectors, respectively. $~{\overrightarrow X _{eq}}$is an equilibrium pool vector.$~\overrightarrow \lambda$ is a random vector in the range [0,1]. *V* is considered a unit.$~\overrightarrow F$is an exponential term that is defined as^64^:

14 $$~\overrightarrow F ={a_1} \times \sin \left( {\overrightarrow {rand} - 0.5} \right) \times \left( {{e^{ - \overrightarrow {\lambda \,\,} \times \,t}} - 1} \right)$$

where, the time *t* is a function of iteration (*T*), which can be defined as:

15 $$~t={\left( {1 - ~\frac{T}{{{T^{\hbox{max} }}}}} \right)^{\left( {{a_2} \times \frac{T}{{{T^{\hbox{max} }}}}} \right)}}$$

where, *a*_*1*_ and *a*_*2*_ are constant values for control of the exploration and exploitation, respectively. The values of *a*_*1*_ and *a*_*2*_ are assumed to be 2 and 1, respectively. *T* and *T*^*max*^ are the current and the maximum number of iterations, respectively. The term $~sin\left( {\overrightarrow {rand} ~~ - ~0.5} \right)$ affects diversification and intensification progress.

The generation rate ($~\vec {G}$) is another term used to improve the intensification operator, which can be defined as:

16 $$~\vec {G}={\vec {G}_O} \times ~~{e^{ - \overrightarrow {\lambda ~~~} \left( {t - ~{t_o}} \right)}}$$

where,$~{\vec {G}_O}$is the initial value of the generation rate, which is formulated as^64^:

17 $$~{\vec {G}_O}=\overrightarrow {GCP} \times \left( {{{\overrightarrow X }_{eq}} - \overrightarrow {\lambda ~~~} \times \overrightarrow X ~} \right)$$

where, $~\overrightarrow {GCP}$ is the generation rate control parameter that can be updated based on a probability *GP* as follows:

18 $$~\overrightarrow {GCP} =\left\{ {\begin{array}{*{20}{c}} {0.5\,{r_1}\,\,\,\,\,\,\,\,\,\,for~~\,{r_2} \geqslant GP} \\ {0\,\,\,\,\,\,\,\,\,\,\,\,\,\,\,\,\,for~~\,{r_2}<GP} \end{array}} \right.$$

where, *r*_*1*_ and *r*_*2*_ are random values in the range [0,1]. *GP* is a generation probability that takes a specified value. The value of *GP* is assumed to be 0.5 for the best balance between exploration and exploitation.

### Solving the EELD problem using EOA

Two individual OFs are presented to reduce total fuel cost with and without valve loading effects and minimize the total emission. In addition, the multi-OF, which aims to reduce these objectives simultaneously, is also presented. The steps of the proposed EOA to solve the EELD problem are presented as follows:

*Step 1: Initialization*.

- Insert the control variables that represent the real generated power randomly, between their minimum and maximum limits, and construct the initial concentration vector *v*_*i*_ for each particle.
- Insert both cost and emission coefficients (*a*_*i*_, *b*_*i*_, *c*_*i*_, *d*_*i*_, *e*_*i*_, *α*_*i*_, *β*_*i*_, *γ*_*i*_, *ζ*_*i*_, and *λ*_*i*_) for each generation unit. Also, insert the *B*-coefficients.
- Define the EOA parameters, number of particles (*n*), *a*_*1*_, *a*_*2*_, and *GP*.
- Create the search space which contains the initial concentration vectors for all control variables.

*Step 2: Initial evaluation*.

The initial values of the OFs in Eqs. (1)-(4) are obtained based on the initial values of the control variables such as:

19 $$OF_{i}^{{init}}=\left[ {OF_{1}^{{init}},OF_{2}^{{init}},OF_{3}^{{init}},\,\cdots.,OF_{n}^{{init}}} \right]$$

*Step 3: Check the constraints*.

For each particle, check the constraints in Eqs. (5)-(10) to exclude the values of OFs that correspond to the index of violation constraints.

*Step 4: Initial global best solution*.

The initial global best solution of the OF ($OF_{{best}}^{{initial}}$) can be determined among the accepted solutions.

*Step 5: Extract the equilibrium pool and candidates*.

For each particle, determine the equilibrium pool and candidate vectors plus the average value of candidates using Eq. (12).

*Step 6: Form*$~\overrightarrow F ,\,\,\overrightarrow {GCP} ,\,\,{\overrightarrow G _O}$*and*$~\overrightarrow G$*vectors*.

For each particle, form the vectors $~\overrightarrow F ,\,\,\overrightarrow {GCP} ,\,\,{\overrightarrow G _O}$and $~\overrightarrow G$using Eqs. (14), (18), (17), and (16), respectively.

*Step 7: Update the concentrations*.

Update the concentration for each particle using Eq. (13).

*Step 8: Generate new solutions*.

After updating the search space, the values of the OFs in Eqs. (1)-(4) are obtained based on the updated values of the control variables as:

20 $$OF_{i}^{k}=\left[ {OF_{1}^{k},OF_{2}^{k},OF_{3}^{k},\cdots\cdots,OF_{n}^{k}} \right]$$

*Step 9: Check the constraints*.

For each particle, check the constraints in Eqs. (5)-(10) to exclude the values of OFs that correspond to the index of violation constraints.

*Step 10: Update the best global solution*.

The best global solution at iteration *k +* 1 can be determined as:

21 $$OF_{{global}}^{{k+1}}=\left\{ \begin{gathered} \,OF_{{best}}^{{k+1}}~~~~~~if\,OF_{{best}}^{{k+1}}<OF_{{best}}^{k} \hfill \\ \,OF_{{best}}^{k}~~~~~~\,otherwise{\text{ }} \hfill \\ \end{gathered} \right.$$

*Step 11: Check the stopping criterion*.

Repeat steps 5 to 10 until reaching the maximum number of iterations.

**Fig. 1:**
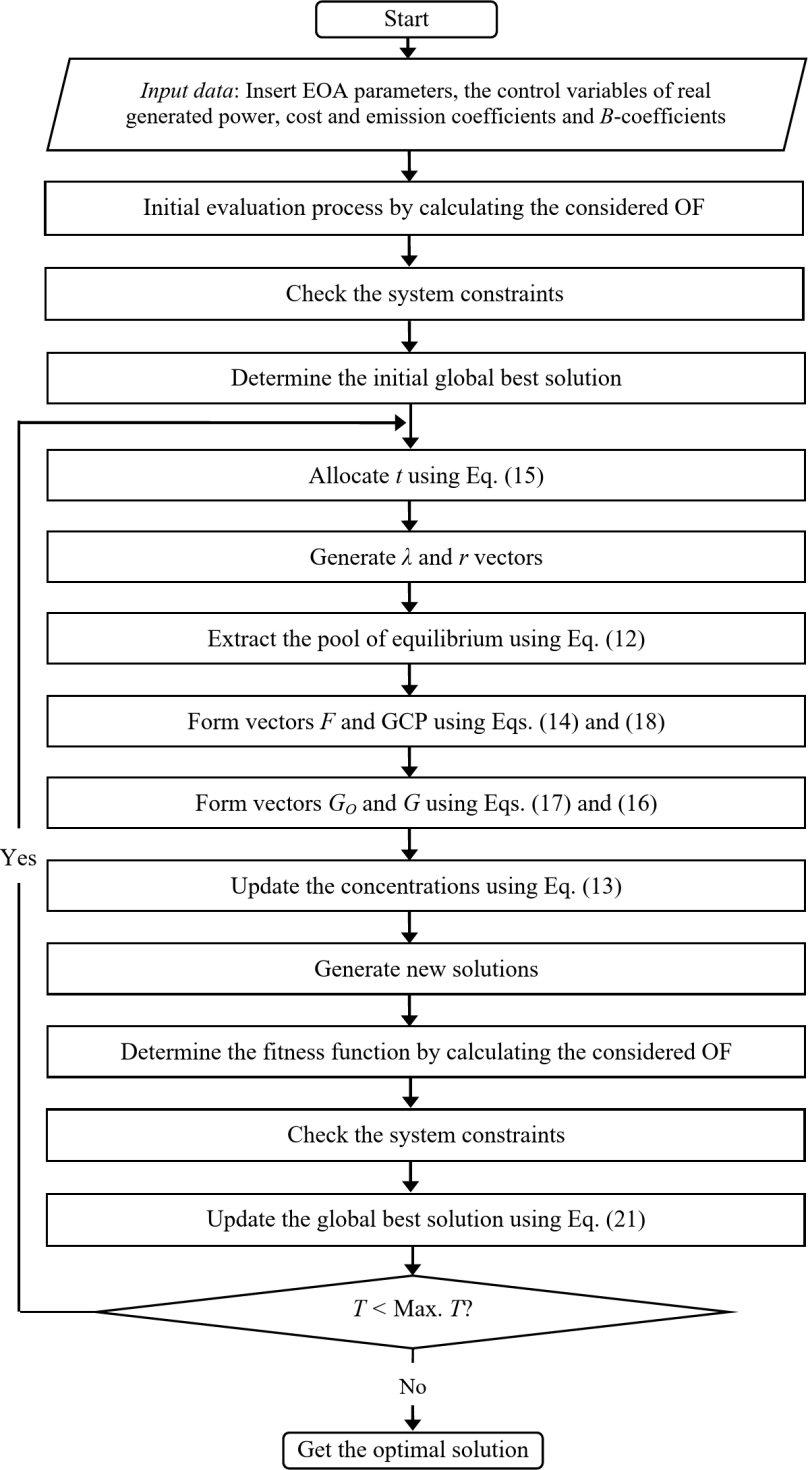
Flow chart of the proposed EOA to find the optimal solution.

The flow chart of the proposed EOA to solve the EELD problem is shown in Fig. 1.

## Applications

### Test systems

The proposed methodology is applied to small and large-scale test systems, having 10, 20, 40, and 80 generation units to solve the EELD problem. The results obtained by the proposed algorithm are compared with those obtained using other methods such as DEA, PSO, and GWO. The data of generation power limits and the coefficients of fuel cost and emission for test systems are taken from Refs^23,27,29,42,48–50,71^., and^22,72^for 10, 20, 40, 80, and 140 generation units, respectively. The data of VPE, RRL, and POZs for 140-unit test system is taken from^22,72^. The total power demands for 10, 20, 40, 80, and 140 generation unit systems are 2000 MW, 2500 MW, 10,500 MW, 21,000 MW, and 49,342 MW, respectively. The main parameters used in the proposed EOA, and other optimization methods are illustrated in the Table 1. Table 1 presents the parameters that are used in the proposed EOA, and other optimization techniques.

**Table 1:** Parameters used for the proposed EOA and other optimization techniques.

Algorithm	Parameter	Value
EOA^64^	Constant for control the exploration ability (*a*_1_)	2
	Constant for control the exploration ability (*a*_2_)	1
	Generation probability (*GP*)	0.5
	Initialization constant (*V*)	1
GWO^73^	Linearly vector ($~\overrightarrow {a~~~}$)	[0,2]
	Coefficient vector ($~\overrightarrow {C~~~}$)	[0,2]
PSO [20]	Minimum inertia weight (*w*_*min*_)	0.4
	Maximum inertia weight (*w*_*max*_)	0.9
	Cognitive constant (*C*_1_)	2
	Social constant (*C*_2_)	2
DEA^28^	Crossover constant (*CR*)	0.5
	Mutation constant (*F*)	0.6

### Studied cases

Ten cases are considered in this paper to study the capability of the proposed algorithm for solving the EELD problem. These cases are summarized in Table 2.

**Table 2:** Summary of the studied cases.

Test system	Objective function	Case#	Description
10-unit 20-unit 40-unit 80-unit	Single OF	Case 1	Minimization of generation fuel cost without VPE
		Case 2	Minimization of generation fuel cost with VPE
		Case 3	Minimization of total emission
	Multi-OF	Case 4	Minimization of generation fuel cost and emission
		Case 5	Minimization of generation fuel cost with VPE and emission
140-unit	Single OF	Case 6	Minimization of generation fuel cost without VPE, RRL, and POZs
		Case 7	Minimization of generation fuel cost with VPE, RRL, and POZs
		Case 8	Minimization of total emission
	Multi-OF	Case 9	Minimization of generation fuel cost and emission with Pareto optimal front
		Case 10	Minimization of generation fuel cost and emission considering VPE, RRL, and POZs with Pareto optimal front

### Results and comments

The proposed approach is carried out using MATLAB installed on a PC with an Intel Core i7 and 8 GB of RAM.

#### 10-unit system

Table 3shows the optimal results obtained using the proposed algorithm and other methods without considering power losses for Cases 1–5 for 10-unit system. For cases 1 and 2 that solve the single OF, it can be observed that the total cost without/with VPE using the proposed EOA is lower than that obtained using DEA, PSO, GWO, and PHOA^49^. The total fuel cost obtained using the proposed EOA is reduced by 0.1414%, and 0.0753% than the PSO base case with savings of 150 $/hr, and 80 $/hr for Cases 1 and 2, respectively. For Case 3, the total emission obtained using the proposed EOA is lower than that obtained using other optimization techniques with a maximum percentage reduction of 1.7483% than the base case. Moreover, the total emission obtained using PHOA reported in^49^ is incorrect. The exact values of the total emission are 235.9897 ton/hr and 120.1085 ton/hr for Cases 1 and 3, respectively. For Cases 4 and 5, which investigate the multi-OF, the proposed EOA gives better results than other methods for minimizing the total fuel cost and emission with savings in the total fuel cost by 70 $/hr, and 1700 $/hr than the base case for cases 4 and 5, respectively. In addition, the total emission is reduced by 4.4067%, and 1.7456% than the base case. Therefore, this comparison reflects the superiority of the proposed EOA in finding the optimal solutions by reducing the total cost and emission.

**Table 3:** Simulation results using different algorithms without considering power losses for 10-unit system.

Case#	Methods	PG_1_ (MW)	PG_2_ (MW)	PG_3_ (MW)	PG_4_ (MW)	PG_5_ (MW)	PG_6_ (MW)	PG_7_ (MW)	PG_8_ (MW)	PG_9_ (MW)	PG_10_ (MW)	Total cost ($/hr)	Emission (ton/hr)	Saving ($/hr)	Reduction in cost and emission (%)
**Case 1**	**PHOA** ^49^	55.0000	80.0000	98.2792	73.2943	70.2278	72.7025	270.4959	340.0000	470.0000	470.0000	1.0621 × 10^5^	4285.4729^a^	**−100**	**−0.0942**
	**DEA**	49.7027	75.5900	105.7511	52.0625	86.7307	85.9315	282.0520	326.7019	465.4775	470.0000	1.0626 × 10^5^	230.4016	**−150**	**−0.1414**
	**PSO**	55.0000	80.0000	81.5810	55.6544	77.8345	77.8024	300.0000	340.0000	462.1277	470.0000	1.0611 × 10^5^	231.4814	**Base case**	**Base case**
	**GWO**	55.0000	75.3325	97.0277	63.8216	77.1391	70.9138	296.5315	324.7287	469.5355	469.9696	1.0603 × 10^5^	235.9674	**80**	**0.0754**
	**Proposed EOA**	54.9934	79.9998	88.8660	79.8392	66.6021	70.0010	289.5393	330.1612	469.9995	469.9985	**1.0596 × 10** ^**5**^	236.4282	**150**	**0.1414**
**Case 2**	**DEA**	55.0000	74.4594	80.5467	81.8027	75.2403	80.4598	300.0000	340.0000	442.4911	470.0000	1.0634 × 10^5^	214.9090	**−90**	**−0.0847**
	**PSO**	55.0000	80.0000	80.2522	87.0732	65.2755	70.0000	300.0000	340.0000	452.3991	470.0000	1.0625 × 10^5^	223.1825	**Base case**	**Base case**
	**GWO**	55.0000	79.4336	85.1591	75.1395	63.9406	70.4830	300.0000	332.3898	468.4544	470.0000	1.0619 × 10^5^	236.6569	**60**	**0.0565**
	**Proposed EOA**	54.9991	79.9975	87.6977	78.9955	66.5905	70.0000	290.5318	331.1880	469.9999	470.0000	**1.0617 × 10** ^**5**^	236.7061	**80**	**0.0753**
**Case 3**	**PHOA** ^49^	55.0000	68.0479	73.4161	70.4446	160.0000	240.0000	275.2700	289.1154	371.9836	396.7219	1.1182 × 10^5^	3661.8815^b^	-	**−28.3128** ^**c**^
	**DEA**	47.1105	80.0000	120.0000	122.8291	160.0000	240.0000	290.9278	304.9213	314.4165	319.7948	1.1300 × 10^5^	93.8757	-	**−0.2881**
	**PSO**	55.0000	80.0000	120.0000	130.0000	160.0000	240.0000	300.0000	340.0000	287.5000	287.5000	1.1360 × 10^5^	93.6060	-	**Base case**
	**GWO**	55.0000	80.0000	120.0000	130.0000	160.0000	240.0000	290.0601	307.5769	310.7960	306.5669	1.1319 × 10^5^	91.9960	-	**1.72**
	**Proposed EOA**	55.0000	80.0000	120.0000	129.9999	160.0000	240.0000	287.3821	311.3577	308.0722	308.1881	1.1320 × 10^5^	**91.9695**	-	**1.7483**
**Case 4**	**DEA**	54.4965	79.1317	120.0000	104.5246	132.3285	180.9431	290.3285	336.7214	345.3231	356.2027	1.0967 × 10^5^	108.6897	**−170**	**−0.1553**,** 4.0548**
	**PSO**	46.7362	80.0000	116.3626	130.0000	94.7519	187.0420	298.1378	340.0000	350.3947	356.5748	1.0950 × 10^5^	113.2831	**Base case**	**Base case**
	**GWO**	54.7963	61.0399	103.9788	124.4775	125.1084	176.1284	298.8834	340.0000	354.6344	360.9529	1.0946 × 10^5^	114.0998	**40**	**0.0365**,**−0.7209**
	**Proposed EOA**	54.9866	79.9999	119.9968	128.4341	137.4033	154.4185	299.0185	331.1013	348.0107	346.6303	**1.0943 × 10** ^**5**^	**108.2911**	**70**	**0.0639**,**4.4067**
**Case 5**	**DEA**	54.2965	79.7724	116.2018	95.0192	150.7132	167.7959	293.7027	333.5432	353.3094	355.6457	1.0983 × 10^5^	109.6576	**−70**	**−0.0638**,** 0.7273**
	**PSO**	41.9993	80.0000	120.0000	124.7120	142.5894	158.8998	292.2151	332.7795	351.5694	355.2353	1.0976 × 10^5^	110.4610	**Base case**	**Base case**
	**GWO**	54.6239	77.2864	118.6727	130.0000	126.1082	167.6864	296.8275	327.1831	353.4531	348.1587	1.0964 × 10^5^	109.0389	**120**	**0.1093**,** 1.2874**
	**Proposed EOA**	54.9998	80.0000	119.9280	129.6212	136.2215	154.7365	300.0000	326.2905	349.3834	348.8191	**1.0959 × 10** ^**5**^	**108.5328**	**170**	**0.1549**,**1.7456**

^a^The exact value of total emission is 235.9897 ton/hr, which is lower than that reported in^49^.

^b^The exact value of total emission is 120.1085 ton/hr, which is lower than that reported in^49^.

^c^ The percentage reduction is determined based on the exact value of the total emission.

**Fig. 2:**
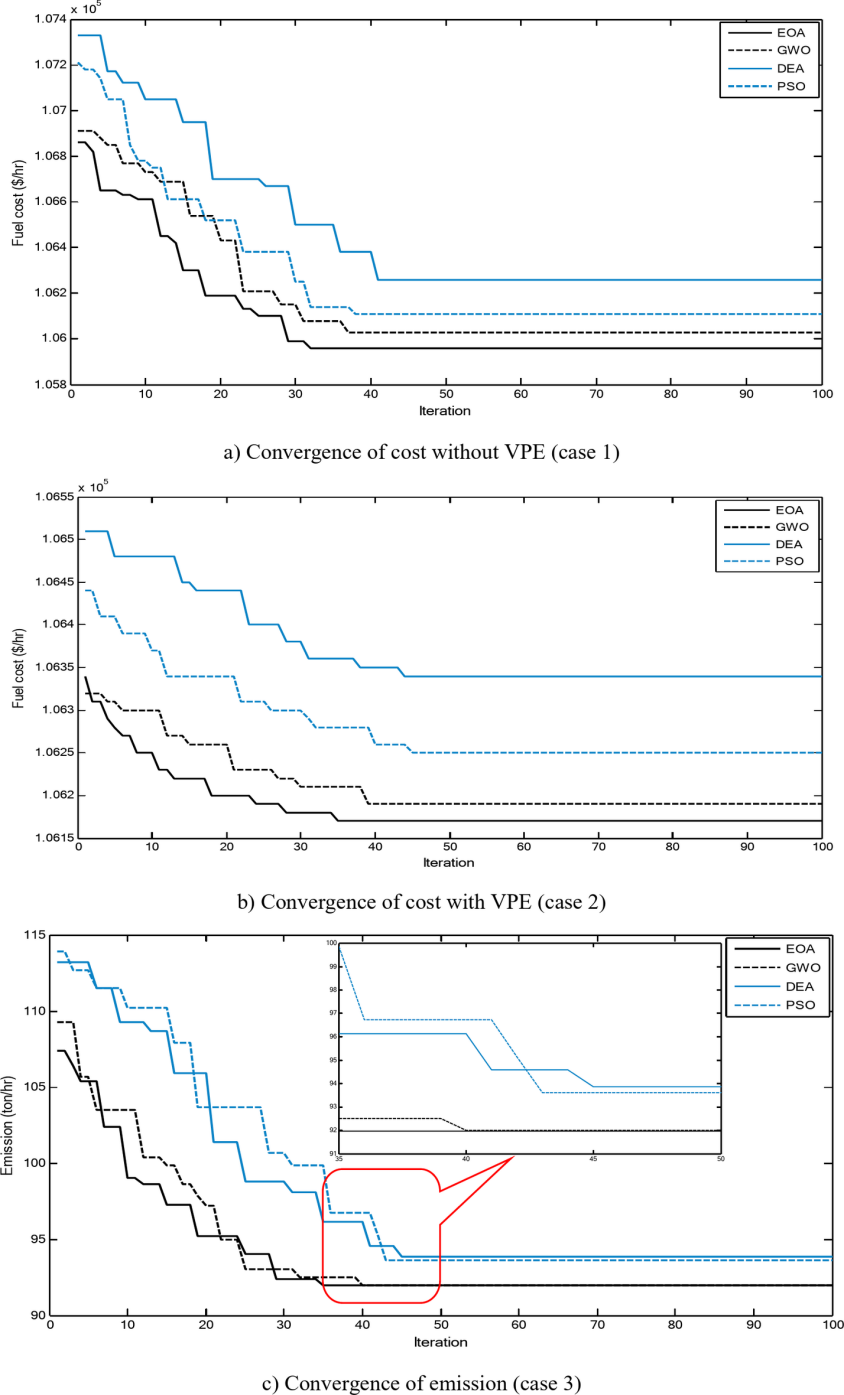
Convergence curves of the proposed EOA and other algorithms without considering power losses for 10-unit system.

Figure 2 shows a comparison between the convergence curves recorded by running the proposed EOA, GWO, DEA, and PSO without considering power losses for cases 1–3 for 10-unit system. The proposed EOA gives fast convergence curves to obtain optimal solutions with a minimum number of iterations.

Table 4shows a comparison between the proposed EOA and other methods for minimizing the total fuel cost with and without VPE in cases 1 and 2 and minimizing the total emission in case 3 for 10-unit system. It can be noticed that the proposed EOA gives minimum values of the OFs than obtained using other methods for all cases. Moreover, the results of total emission obtained using MOMSA^22^, OWP-based OMF^38^, TFWO^46^, and PHOA^49^ are incorrect. The exact values of the total emission are 136.5409 ton/hr, 134.7503 ton/hr, 238.6139 ton/hr, and 235.9897 ton/hr for these algorithms, respectively. Therefore, this comparison reflects the superiority of the proposed EOA for minimizing the total fuel cost and total emission individually as single OFs.

**Table 4:** Comparison between the single OFs using the proposed EOA and other methods for 10-unit system (Cases 1–3).

Method	Case 1	Case 2	Case 3
Proposed EOA	1.0596 × 10^5^	1.0617 × 10^5^	91.9695
GWO	1.0603 × 10^5^	1.0619 × 10^5^	91.9960
PSO	1.0611 × 10^5^	1.0625 × 10^5^	93.6060
DEA	1.0626 × 10^5^	1.0634 × 10^5^	93.8757
MPSO_SSM^20^	1.1321 × 10^5^	N/A	N/A
MOMSA^22^	1.1150 × 10^5^	N/A	3933.845^a^
FPA^22^	1.1181 × 10^5^	N/A	N/A
BA^23^	1.1247 × 10^5^	N/A	N/A
ELD^38^	1.11497 × 10^5^	N/A	N/A
OWP-based OMF^38^	N/A	1.11497 × 10^5^	3932.2538^b^
TFWO^46^	1.3300 × 10^5^	1.12148 × 10^5^	4516.249847^c^
PHOA^49^	1.0621 × 10^5^	N/A	3661.8815^d^

N/A: Not available.

^a^ The exact value of total emission is 136.5409 ton/hr, which is lower than that reported in^22^.

^b^ The exact value of total emission is 134.7503 ton/hr, which is lower than that reported in^38^.

^c^ The exact value of total emission is 238.6139 ton/hr, which is lower than that reported in^46^.

^d^ The exact value of total emission is 235.9897 ton/hr, which is lower than that reported in^49^.

Table 5shows a comparison between the proposed EOA and other methods for minimizing the total fuel cost and emission simultaneously with and without VPE for 10-unit system. The total fuel cost and emission obtained using the proposed EOA are lower than those obtained using other methods for all cases. In addition, the results of total emission obtained using MOMSA^22^, QOPO^24^, and OWP-based OMF^38^ are incorrect. The exact values of the total emission are 170.3037 ton/hr, 116.1860 ton/hr, and 136.1811 ton/hr for these algorithms, respectively. This comparison reflects the great capability of the proposed EOA to solve the multi-OF.

**Table 5:** Comparison between the multi-OF using the proposed EOA and other methods for 10-unit system (Cases 4,5).

Method	Case 4		Case 5	
	Total fuel cost ($/hr)	Total emission (ton/hr)	Total fuel cost ($/hr)	Total emission (ton/hr)
Proposed EOA	1.0943 × 10^5^	108.2911	1.0959 × 10^5^	108.5328
GWO	1.0946 × 10^5^	114.0998	1.0964 × 10^5^	109.0389
PSO	1.0950 × 10^5^	113.2831	1.0976 × 10^5^	110.4610
DEA	1.0967 × 10^5^	108.6897	1.0983 × 10^5^	109.6576
MOMSA^22^	1.1349 × 10^5^	4109.035^a^	N/A	N/A
FPA^23^	1.1564 × 10^5^	321.822	N/A	N/A
BA^23^	1.13795 × 10^5^	325.252	N/A	N/A
QOPO^24^	N/A	N/A	1.11892 × 10^5^	3653.34^b^
OWP-based OMF^38^	N/A	N/A	1.16391 × 10^5^	3932.4035^c^

#### 20-unit system

Table 6 shows the optimal results obtained using the proposed algorithm and other methods with and without considering power losses for case 1 for 20-unit system. It can be observed that the total cost obtained using the proposed EOA is lower than that obtained using other methods. The total fuel cost obtained using the proposed EOA is reduced by 0.1295%, and 0.2536% than the base case (results of PSO) with savings of 78 $/hr, and 158 $/hr. Moreover, the results of total power loss obtained using BSA^50^and BBA^40^are incorrect. The exact values of total power loss are 86.1602 MW and 85.6647 MW for BSA^50^and BBA^40^, respectively. Therefore, this comparison reflects the great capability of the proposed EOA to reduce the total fuel cost with a maximum saving and percentage reduction in the total fuel cost.

**Fig. 3:**
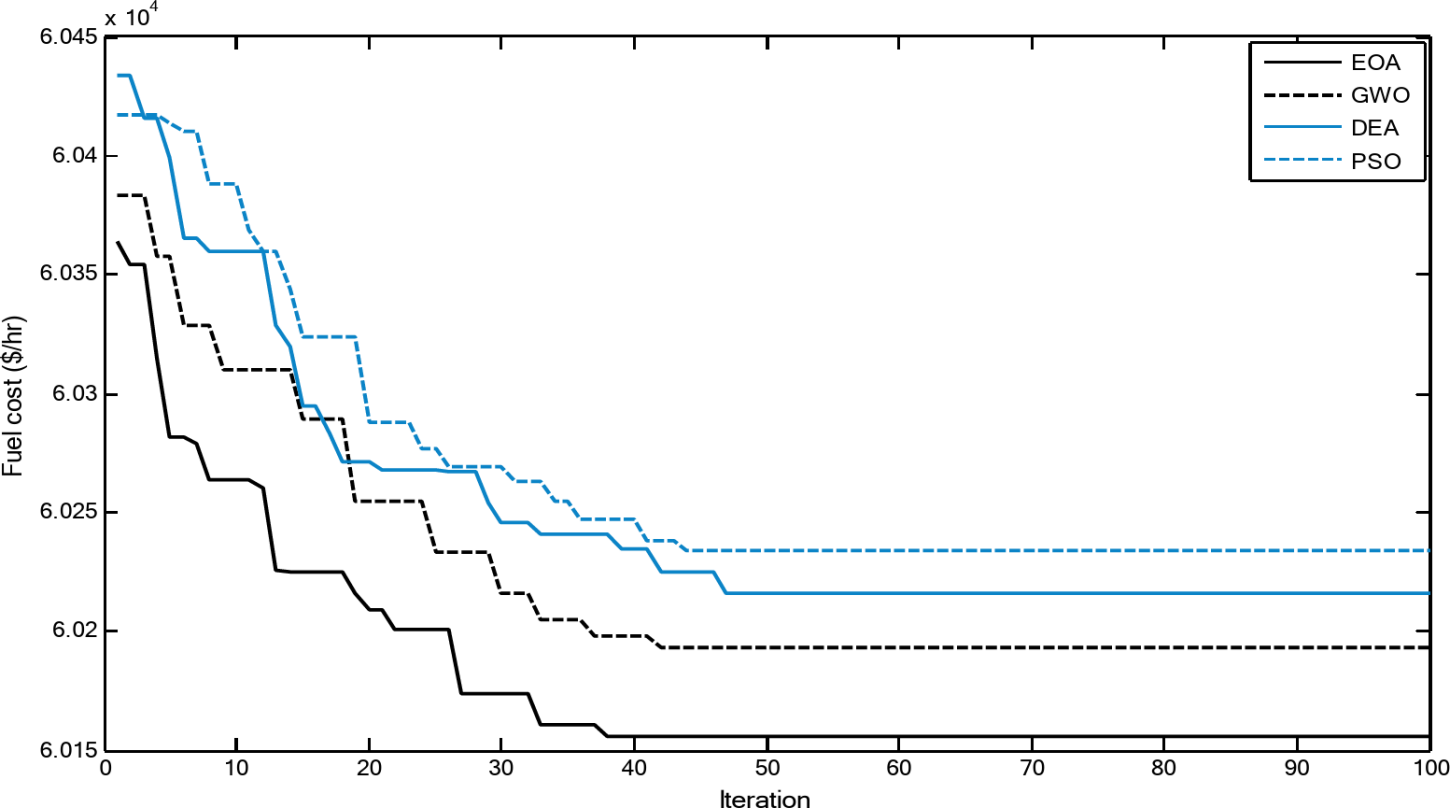
Convergence curves of the proposed EOA and other algorithms without considering power losses for 20-unit system (Case 1).

**Fig. 4:**
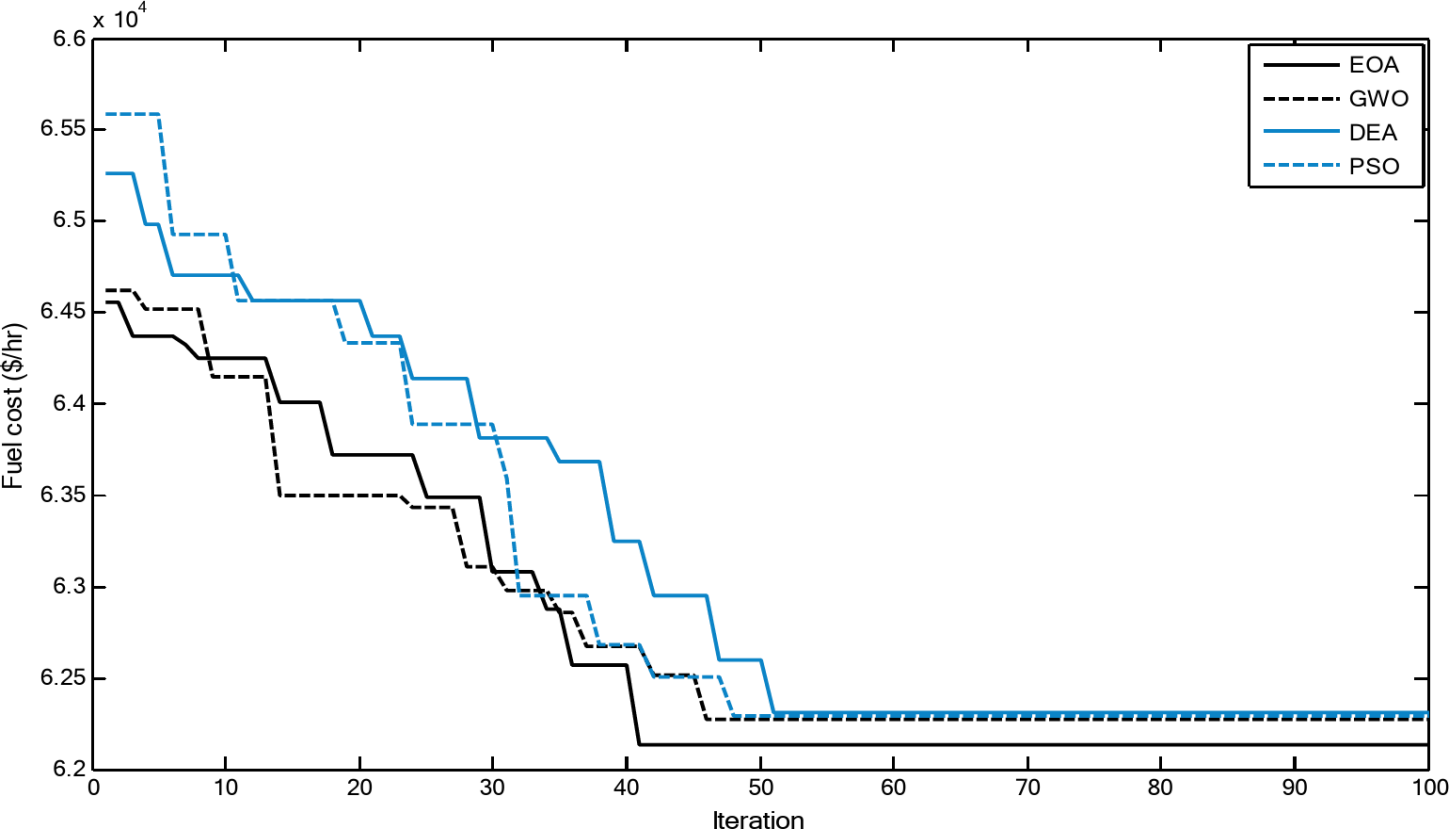
.Convergence curves of the proposed EOA and other algorithms considering power losses for 20-unit system (Case 1).

Figures 3 and 4 show comparisons between the convergence curves recorded by running the proposed EOA, GWO, DEA, and PSO with and without considering power losses for case 1 for 20-unit system. The proposed EOA has a great capability for reaching the optimal solution with a minimum number of iterations compared with other methods.

**Table 6:** Simulation results using different algorithms with and without considering power losses for 20-unit system (Case 1).

Unit (MW)	Without considering power losses				Considering power losses					
	DEA	PSO	GWO	Proposed EOA	BSA^50^	BBO^40^	DEA	PSO	GWO	Proposed EOA
**PG**_**1**_	590.1193	599.8953	585.6155	599.9904	510.4477	513.0892	507.3634	499.2181	526.1767	576.4824
**PG**_**2**_	119.4657	50.0000	94.5697	155.5490	168.3973	173.3533	184.5956	182.1609	151.2558	154.0055
**PG**_**3**_	50.0000	50.0000	56.9854	50.0004	125.9721	126.9231	116.8091	100.2173	131.5119	106.4809
**PG**_**4**_	75.4933	51.4731	58.0260	51.3003	103.5291	103.3292	108.9801	102.2124	105.6489	101.6510
**PG**_**5**_	96.7198	159.5090	100.6521	92.5731	113.8218	113.7741	110.2493	132.9044	103.9641	113.7186
**PG**_**6**_	33.6557	20.0000	23.8110	25.2951	73.7901	73.0669	62.9342	63.8970	56.7748	56.0358
**PG**_**7**_	96.4455	124.7846	125.0000	124.9982	115.0664	114.9843	100.4223	101.3374	106.1682	103.1087
**PG**_**8**_	63.6076	50.2154	56.1031	50.0209	116.3401	116.4238	101.1378	106.0637	121.9277	114.9353
**PG**_**9**_	111.3790	50.0000	95.5021	115.0173	100.7093	100.6948	122.1784	114.2827	106.7044	121.2489
**PG**_**10**_	45.2964	49.8183	52.5347	34.5347	107.1366	99.998	122.4164	102.4863	108.1397	93.3365
**PG**_**11**_	300.0000	299.0180	300.0000	288.8634	150.7060	148.9770	142.6052	139.8769	141.0712	140.8361
**PG**_**12**_	394.8572	443.2583	476.3256	433.8479	291.1304	294.0207	283.3382	280.2072	280.1421	295.4732
**PG**_**13**_	86.6005	119.5617	113.2458	120.3679	119.1528	119.5754	101.6246	118.9740	105.4251	121.3481
**PG**_**14**_	94.6171	130.0000	71.7165	59.1153	32.4521	30.5479	44.8411	49.0385	83.8805	66.3473
**PG**_**15**_	88.9110	78.4021	52.4695	93.0795	116.1479	116.4546	110.2168	126.2255	100.9735	99.8723
**PG**_**16**_	36.1381	36.0756	36.2869	36.0305	36.2816	36.2279	43.7435	55.3976	34.0279	29.0350
**PG**_**17**_	48.0895	37.8243	33.9607	30.0218	67.7355	66.8594	78.1829	56.8549	55.4891	54.7175
**PG**_**18**_	55.2177	41.9559	45.7686	33.4837	87.2547	88.5470	87.8959	84.1129	115.4591	76.2522
**PG**_**19**_	83.2787	78.1037	87.3999	75.9108	101.5359	100.9802	98.1346	112.0895	95.2552	105.4336
**PG**_**20**_	30.1079	30.1047	34.0269	30.0000	54.2861	54.2725	55.9538	56.2153	50.7753	50.4279
**Total PG (MW)**	2500	2500	2500	2500	2591.8930	2592.1011	2583.6	2583.8	2580.8	2580.7
**Total loss (MW)**	NC	NC	NC	NC	91.8930^a^	92.1011^b^	83.6232	83.7725	80.7712	80.7468
**Total cost ($/hr)**	6.0216 × 10^4^	6.0234 × 10^4^	6.0193 × 10^4^	**6.0156 × 10** ^**4**^	6.24566 × 10^4^	6.24567 × 10^4^	6.2311 × 10^4^	6.2294 × 10^4^	6.2271 × 10^4^	**6.2136 × 10** ^**4**^
**Saving ($/hr)**	**18**	**Base case**	**41**	**78**	**−162.6**	**−162.7**	**−17**	**Base case**	**23**	**158**
**Reduction (%)**	**0.0299**	**Base case**	**0.0681**	**0.1295**	**−0.2610**	**−0.2612**	**−0.0273**	**Base case**	**0.0369**	**0.2536**

#### 40-unit system

Table 7 shows the optimal results obtained using the proposed algorithm and other methods without considering power losses for cases 1 and 2, which aim to minimize the total fuel cost for 40-unit system. It can be observed that the total cost without and with the VPE obtained using the proposed EOA is lower than that obtained using other methods. The proposed EOA gives better solutions with maximum savings in the total fuel cost by 820 $/hr and 3610 $/hr with a percentage reduction of 0.6864%, and 2.8891% than the base case (results of PSO) for cases 1 and 2, respectively. Moreover, the total cost with VPE obtained using EMFO reported in^48^ is incorrect. The exact value of the total cost is 1.3038 × 10^5^ $/hr. Therefore, this comparison reflects the superiority of the proposed EOA for reducing the total cost without and with the VPE as a single OF.

Table 8shows the optimal results obtained using the proposed algorithm and other methods without considering power losses for case 3, which aims to minimize the total emission for 40-unit system. The total emission obtained using the proposed EOA is lower than that obtained using other methods. The total emission obtained using the proposed EOA is reduced by 12.8673% than the base case (results of PSO). Moreover, the total generation power (total PG) obtained using EMFO reported in^48^is lower than the total load demand, which violates the equality constraint between the total generation power and the total load. In addition, the total emission obtained using EMFO reported in^48^ is incorrect. The exact value of the total emission is 0.98519 × 10^5^ ton/hr. Therefore, this comparison reflects the great capability of the proposed EOA to find the optimal solution for reducing the total emission as a single OF.

**Table 7:** Simulation results using different algorithms without considering power losses for cases 1 and 2 for 40-unit system.

Unit (MW)	Case 1					Case 2				
	EMFO [48]	DEA	PSO	GWO	Proposed EOA	EMFO^48^	DEA	PSO	GWO	Proposed EOA
**PG**_**1**_	110.8000	113.7425	114.0000	112.7622	113.9939	72.4810	109.3649	103.5694	111.9383	111.313
**PG**_**2**_	110.8300	109.5840	114.0000	113.8601	113.9917	103.0314	113.7127	101.5144	112.1129	111.998
**PG**_**3**_	97.4000	103.1941	68.3115	116.5891	119.3923	83.2726	109.2182	102.7456	115.7113	97.401
**PG**_**4**_	179.7300	186.2181	189.4817	171.2819	189.8808	182.3106	180.0701	179.4893	181.8185	179.733
**PG**_**5**_	87.8100	52.8047	85.0929	89.2021	97.0000	76.16690	47.1511	88.3478	91.4447	88.286
**PG**_**6**_	140.0000	131.8428	137.0624	87.7816	139.9897	126.1346	68.1194	116.6377	139.7743	140.000
**PG**_**7**_	259.6000	298.3631	300.0000	298.6929	299.9906	258.8452	260.8024	260.9451	269.9102	259.686
**PG**_**8**_	284.6000	293.5636	300.0000	300.0000	299.9888	297.1636	289.8194	299.4184	287.8571	284.686
**PG**_**9**_	284.6000	291.6018	271.5001	297.7745	300.0000	290.8899	300.0000	294.4794	299.1560	284.648
**PG**_**10**_	130.0000	143.0376	131.1118	141.5428	130.4154	274.8232	130.0000	134.0914	208.2818	130.000
**PG**_**11**_	94.0000	185.9344	101.9670	99.4702	94.3739	356.9806	94.6843	239.5202	243.6188	168.800
**PG**_**12**_	94.0000	109.1644	99.6541	141.4999	94.0000	124.4054	94.9139	137.4244	94.2880	168.800
**PG**_**13**_	214.7600	205.2311	125.8488	149.0470	125.0363	493.3764	484.2977	231.0112	215.8268	214.760
**PG**_**14**_	394.2800	259.0914	378.9173	290.5184	263.9599	344.9029	483.8292	362.4239	304.6650	304.520
**PG**_**15**_	394.2800	374.1713	295.2477	275.5262	265.6368	372.3864	396.1995	401.2256	394.2111	394.279
**PG**_**16**_	394.2800	199.8125	300.8785	255.8366	275.1177	345.4624	307.4974	307.8325	308.0857	394.279
**PG**_**17**_	489.2800	491.5980	493.6305	496.5113	499.9912	422.6378	491.2989	500.0000	491.1036	489.280
**PG**_**18**_	489.2800	500.0000	494.3846	495.7260	499.9999	434.4065	492.7472	489.5268	490.6632	489.280
**PG**_**19**_	511.2800	537.4658	544.4749	546.8444	549.9537	461.3107	549.6755	529.5771	512.1506	511.280
**PG**_**20**_	511.2800	512.5663	550.0000	546.5685	549.9891	434.3828	511.6987	516.8540	520.6192	511.280
**PG**_**21**_	523.2800	547.8014	550.0000	549.3160	549.9991	545.2846	527.6592	533.3350	525.2025	523.280
**PG**_**22**_	523.2800	550.0000	548.9185	549.8513	549.9976	490.3572	530.0630	526.4301	525.4243	523.281
**PG**_**23**_	523.2800	549.8647	550.0000	549.2702	549.9957	506.0639	534.7742	538.9574	533.9092	523.292
**PG**_**24**_	523.2800	546.7106	550.0000	549.4001	549.9983	467.3109	529.4045	527.0739	544.2299	523.295
**PG**_**25**_	523.2800	547.3854	549.1253	547.8111	550.0000	488.1203	527.7690	537.2021	538.6999	523.286
**PG**_**26**_	523.2800	550.0000	550.0000	549.2038	549.9979	486.9091	526.6999	525.9084	548.3362	523.288
**PG**_**27**_	10.0000	22.6329	15.0288	20.4894	10.3777	16.8002	10.1911	29.4482	13.1744	10.001
**PG**_**28**_	10.0000	17.8608	10.0203	15.2850	10.0049	39.3475	10.0000	26.9607	10.2931	10.001
**PG**_**29**_	10.0000	16.6034	13.6060	13.5449	10.0007	23.6359	10.1030	10.1148	13.3658	10.030
**PG**_**30**_	87.9300	97.0000	49.6644	96.2780	97.0000	86.3295	87.6388	88.7939	49.9024	89.229
**PG**_**31**_	190.0000	184.2557	190.0000	189.4573	189.9968	165.9924	188.7021	188.3093	189.4597	190.000
**PG**_**32**_	190.0000	190.0000	189.0069	190.0000	190.0000	174.5707	188.0284	115.2628	190.0000	190.000
**PG**_**33**_	190.0000	190.0000	190.0000	189.7785	189.9999	184.0570	176.2269	168.3865	189.8296	190.000
**PG**_**34**_	164.8000	196.7967	195.9627	200.0000	200.0000	193.6668	139.2982	165.1321	173.4684	165.000
**PG**_**35**_	194.2200	194.1554	198.2829	200.0000	200.0000	191.6152	197.9548	166.0533	171.3421	165.426
**PG**_**36**_	200.0000	194.2074	197.9483	199.8346	199.9947	196.1763	97.8741	194.6182	200.0000	165.002
**PG**_**37**_	110.0000	81.7037	101.0525	109.8446	109.9542	90.0101	66.1189	100.4049	57.5882	110.000
**PG**_**38**_	110.0000	98.4409	109.9790	107.2475	109.9970	37.5421	94.6512	104.3700	89.2502	110.000
**PG**_**39**_	110.0000	103.2594	109.6439	109.7493	109.9854	89.4239	27.5218	34.4897	30.9007	110.000
**PG**_**40**_	511.2800	522.3341	114.0000	536.6027	549.9984	471.4405	514.2204	522.1145	512.3863	511.280
**Total cost ($/hr)**	1.2039 × 10^5^	1.1998 × 10^5^	1.1947 × 10^5^	1.1914 × 10^5^	**1.1865 × 10** ^**5**^	1.21074 × 10^5a^	1.2468 × 10^5^	1.2502 × 10^5^	1.2344 × 10^5^	**1.21408 × 10** ^**5**^
**Emission (ton/hr)**	3.5991 × 10^5^	4.1426 × 10^5^	4.6272 × 10^5^	4.5634 × 10^5^	4.8821 × 10^5^	1.9226 × 10^5^	3.4534 × 10^5^	3.2223 × 10^5^	3.3784 × 10^5^	2.8588 × 10^5^
**Saving ($/hr)**	**−920**	**−510**	**Base case**	**330**	**820**	**−5360**	**340**	**Base case**	**1580**	**3610**
**Reduction (%)**	**−0.7701**	**−0.4269**	**Base case**	**0.2762**	**0.6864**	**−4.2873**	**0.2720**	**Base case**	**1.2638**	**2.8891**

**Table 8:** Simulation results using different algorithms without considering power losses for case 3 for 40-unit system.

Unit (MW)	EMFO [48]	DEA	PSO	GWO	Proposed EOA
**PG**_**1**_	112.8970	80.0360	96.2703	108.1279	113.9989
**PG**_**2**_	114.0000	54.7755	113.2980	112.7034	113.9617
**PG**_**3**_	119.3100	116.9597	64.7910	117.5331	117.1639
**PG**_**4**_	170.0000	171.4494	167.1702	162.3247	158.5357
**PG**_**5**_	98.0000	88.7911	53.0096	95.7592	97.0000
**PG**_**6**_	127.0000	108.3477	119.2845	121.1412	115.8582
**PG**_**7**_	297.1050	287.7893	296.9227	273.2306	281.3628
**PG**_**8**_	297.1020	280.9017	287.3587	281.6929	281.2955
**PG**_**9**_	297.0000	286.5895	272.7832	287.7084	280.6144
**PG**_**10**_	135.0000	289.8043	292.7528	287.2052	279.4520
**PG**_**11**_	298.0010	294.0234	287.9340	284.2721	280.6241
**PG**_**12**_	296.0000	289.4270	292.8815	277.3760	280.9754
**PG**_**13**_	433.9870	424.8134	423.2242	420.9713	412.7630
**PG**_**14**_	421.0000	422.5442	413.6543	421.6184	413.6677
**PG**_**15**_	423.0230	431.8827	420.4433	414.0262	412.6818
**PG**_**16**_	421.6980	427.1605	406.2808	421.4475	412.7961
**PG**_**17**_	441.3980	432.1009	414.0222	414.5179	413.1261
**PG**_**18**_	436.7750	436.7498	420.8522	424.1612	413.2410
**PG**_**19**_	435.9980	423.7473	419.7882	418.8646	413.1523
**PG**_**20**_	439.3450	436.6625	422.2224	420.0762	412.6896
**PG**_**21**_	436.7950	423.7132	421.2336	424.6677	412.8341
**PG**_**22**_	441.8600	428.5236	427.2699	431.4046	412.7201
**PG**_**23**_	438.4250	433.8020	431.8115	421.7286	413.1669
**PG**_**24**_	438.2890	431.5250	400.7021	418.7168	413.4240
**PG**_**25**_	442.0000	429.4588	423.5722	416.3636	413.0637
**PG**_**26**_	441.5230	429.2610	420.5306	417.4766	413.0864
**PG**_**27**_	29.4587	149.7097	146.6846	58.3812	149.9992
**PG**_**28**_	27.8974	38.7376	148.6261	146.3222	150.0000
**PG**_**29**_	31.4587	145.4200	146.3781	148.0957	150.0000
**PG**_**30**_	98.0000	94.7900	97.0000	82.3113	96.9999
**PG**_**31**_	170.9780	151.0178	153.0211	162.1750	158.6735
**PG**_**32**_	173.6450	98.4941	153.9441	153.7356	157.8839
**PG**_**33**_	173.4820	164.7913	171.8004	159.5147	158.8297
**PG**_**34**_	200.0000	200.0000	175.9004	200.0000	200.0000
**PG**_**35**_	200.0000	200.0000	200.0000	199.8801	200.0000
**PG**_**36**_	200.0000	199.2145	189.6263	200.0000	199.9875
**PG**_**37**_	100.3390	94.1498	102.3508	96.6063	93.4460
**PG**_**38**_	100.3390	78.4659	104.5599	89.3317	94.5717
**PG**_**39**_	100.3390	96.4918	72.1938	91.9680	93.0526
**PG**_**40**_	438.4560	427.8780	427.8504	416.5623	413.3006
**Total PG (MW)**	10,498^a^	10,500	10,500	10,500	10,500
**Total cost ($/hr)**	1.2479 × 10^5^	1.4665 × 10^5^	1.5588 × 10^5^	1.4673 × 10^5^	1.5667 × 10^5^
**Emission (ton/hr)**	1.7648 × 10^5b^	0.82389 × 10^5^	0.76434 × 10^5^	0.72978 × 10^5^	**0.66599 × 10** ^**5**^
**Reduction (%)**	**−28.8942** ^c^	**−7.7910**	**Base case**	**4.5215**	**12.8673**

^a^ The total PG is smaller than the total load demand.

^b^ The exact value of total emission is 0.98519 × 10^5^ ton/hr, which is lower than that reported in [48].

^c^ The percentage reduction is determined based on the exact value of the total emission.

Table 9 presents the optimal results obtained using the proposed algorithm and other methods without considering power losses for cases 4 and 5, which aim to minimize the total fuel cost and emission as a multi-OF for 40-unit system. The proposed EOA gives better results than other methods with maximum savings in the total fuel cost by 1230 $/hr, and 4960 $/hr than the base case (results of PSO) for cases 4 and 5, respectively. In addition, the total emission is reduced by 10.7355%, and 9.2553% than the base case. Moreover, the total generation power (total PG) obtained using EMFO reported in^48^is higher than the total load demand in case 4 and lower than the total load demand in case 5, which violates the equality constraint between the total generation power and the total load. Also, in case 5, the total fuel cost and emission obtained using EMFO reported in^48^ are incorrect. The exact values of total fuel cost and total emission are 1.2855 × 10^5^ $/hr and 3.1999 × 10^5^ ton/hr, respectively. Finally, this comparison reflects the superiority of the proposed EOA for finding the optimal solutions for reducing the total fuel cost and emission simultaneously.

**Table 9:** Simulation results of multi-OF using different algorithms without considering power losses for cases 4 and 5 for 40-unit system.

Unit (MW)	Case 4					Case 5				
	EMFO^48^	DEA	PSO	GWO	Proposed EOA	EMFO^48^	DEA	PSO	GWO	Proposed EOA
**PG**_**1**_	108.0000	41.1089	101.9403	108.4218	113.8165	43.4050	94.3044	92.6034	112.1121	112.8313
**PG**_**2**_	109.0145	57.4596	113.2408	107.7630	113.9814	113.9500	98.1316	80.0318	81.6441	112.3304
**PG**_**3**_	109.0789	106.7802	115.5625	117.2959	94.2411	105.8600	73.8487	60.7702	118.1582	111.8325
**PG**_**4**_	181.0000	174.4763	171.5525	167.4912	170.1354	169.6500	136.3641	182.8740	178.4684	178.9728
**PG**_**5**_	89.0000	87.8401	76.3062	96.1259	93.9931	96.6590	95.4310	80.0220	92.2421	96.7222
**PG**_**6**_	135.0871	85.8669	70.9432	134.5866	101.8396	139.0200	138.7012	104.9378	124.0891	127.0025
**PG**_**7**_	274.0000	295.9179	294.9669	299.4016	295.9777	273.2800	269.0747	298.5290	299.9432	296.6678
**PG**_**8**_	288.0000	290.4694	296.5037	298.4704	296.8671	285.1700	290.5197	291.1272	292.4239	285.8221
**PG**_**9**_	290.0000	294.8703	298.3291	290.8588	295.9529	241.9600	288.1722	295.6427	296.0795	286.7920
**PG**_**10**_	130.0000	286.7670	291.1344	279.2657	284.5850	131.2600	288.2815	292.0252	294.7174	286.9035
**PG**_**11**_	244.2104	304.9215	291.3841	282.7984	284.7747	312.1300	276.9091	307.1969	309.2490	302.9688
**PG**_**12**_	204.0000	267.6270	288.3816	291.7689	278.8724	362.5800	305.7756	298.4633	311.6282	300.5150
**PG**_**13**_	304.0000	422.2810	427.0098	410.0047	422.2201	346.2400	411.6751	393.3583	435.9831	436.4965
**PG**_**14**_	395.0000	444.2181	422.4219	422.4736	424.3765	306.0600	455.5928	438.8936	445.7176	401.2188
**PG**_**15**_	388.0000	385.3036	429.2686	426.6645	421.3856	358.7800	441.9062	436.4506	412.8814	395.4341
**PG**_**16**_	395.1877	438.1469	424.5896	418.9103	421.6770	260.6800	470.4041	450.1466	400.9534	418.7474
**PG**_**17**_	489.0000	444.0532	437.9156	443.0224	437.3026	415.1900	465.4192	460.1062	447.6640	422.3470
**PG**_**18**_	487.2547	441.0517	451.2911	431.1426	430.8004	423.9400	470.6694	451.8917	464.8816	444.8420
**PG**_**19**_	423.9870	442.9408	439.1687	443.0742	434.8538	549.1200	448.8069	430.2045	425.2665	425.0665
**PG**_**20**_	514.0000	432.9765	440.8140	440.2586	437.4438	496.7000	446.4144	464.8289	428.3645	421.6866
**PG**_**21**_	523.0000	443.1943	441.9672	438.2320	436.3130	539.17000	437.0574	428.7801	434.1019	435.3535
**PG**_**22**_	527.0000	456.6090	442.2559	435.6977	435.3771	546.4600	465.5212	433.5187	437.1779	434.5871
**PG**_**23**_	527.0000	464.0147	445.8949	438.8814	437.2458	540.0600	436.7696	439.6049	434.6472	433.5281
**PG**_**24**_	430.0000	443.6726	444.6438	443.4871	439.7233	514.500	429.5835	434.9574	435.5299	434.7364
**PG**_**25**_	525.0000	447.1077	445.7547	441.5247	439.3128	453.4600	437.4342	440.6249	439.1480	433.4402
**PG**_**26**_	434.0000	453.4280	446.5984	443.5628	439.2050	517.3100	451.1207	439.8483	442.6089	433.6312
**PG**_**27**_	30.0000	38.9940	10.2068	37.9420	12.1021	14.8810	85.5891	51.2874	12.3338	28.6070
**PG**_**28**_	45.6587	63.6750	30.8219	21.0547	14.4010	18.7900	51.9567	60.9520	36.5322	14.7988
**PG**_**29**_	64.2548	13.6978	48.5746	42.4593	34.9041	26.6110	50.0013	56.0187	10.5438	12.2109
**PG**_**30**_	88.0987	87.4898	96.5429	96.7595	96.7013	59.5810	60.3872	54.9748	96.3602	94.9067
**PG**_**31**_	162.0870	181.9984	179.6086	178.5164	175.6386	183.4800	152.2627	189.3629	171.1175	186.1796
**PG**_**32**_	183.5470	173.3939	180.9897	173.2484	173.2062	183.3900	163.2983	161.0145	181.4982	175.3195
**PG**_**33**_	183.5000	172.2331	161.7181	179.1242	171.2746	189.0200	165.5693	156.0151	73.0769	173.8934
**PG**_**34**_	172.0000	172.1836	199.7339	199.5589	199.9957	198.7300	97.5757	194.1622	200.0000	200.0000
**PG**_**35**_	162.6012	186.4367	91.5210	200.0000	199.9843	198.7700	187.6552	184.1930	199.6075	200.0000
**PG**_**36**_	175.0000	198.1587	199.7021	199.9830	199.8101	182.2300	184.4160	193.3303	199.9010	199.9262
**PG**_**37**_	94.0000	78.6118	107.3538	49.1035	103.2469	39.6730	43.3665	107.0288	90.3929	105.8799
**PG**_**38**_	112.4580	109.0885	106.5723	99.5663	99.0938	81.5960	106.1806	56.5950	98.6884	109.0521
**PG**_**39**_	97.0000	109.6541	94.4586	35.3264	103.0315	42.9600	97.1878	84.8067	107.0686	105.4785
**PG**_**40**_	422.0000	461.2814	442.3562	436.1726	434.3361	537.1700	430.6651	422.8204	427.1979	423.2711
**Total PG (MW)**	10,516^a^	10,500	10,500	10,500	10,500	10,499^b^	10,500	10,500	10,500	10,500
**Total cost ($/hr)**	1.2510 × 10^5^	1.2719 × 10^5^	1.2615 × 10^5^	1.2598 × 10^5^	**1.2492 × 10** ^**5**^	1.2317 × 10^5c^	1.3529 × 10^5^	1.3334 × 10^5^	1.2989 × 10^5^	**1.2838 × 10** ^**5**^
**Emission (ton/hr)**	2.5026 × 10^5^	1.0991 × 10^5^	1.0456 × 10^5^	0.97028 × 10^5^	**0.93335 × 10** ^**5**^	2.0846 × 10^5 d^	1.1230 × 10^5^	1.0501 × 10^5^	1.0305 × 10^5^	**0.95291 × 10** ^**5**^
**Saving ($/hr)**	**1050**	**−1040**	**Base case**	**170**	**1230**	**4790** ^e^	**−1950**	**Base case**	**3450**	**4960**
**Reduction in cost**,** and emission (%)**	**0.8323**, **−139.3458**	**−0.8244**, **−5.1224**	**Base case**	**0.1348**,** 7.2035**	**0.975**,** 10.7355**	**3.5923**, **−98.5144**	**−1.4624**, **−6.9422**	**Base case**	**2.5874**, **1.8665**	**3.7198**,** 9.2553**

Table 10shows a comparison between the proposed EOA and other methods when considering different single OFs for 40-unit system. It can be observed that the proposed EOA gives minimum values of the OFs than those obtained using other methods for all cases. Moreover, the total fuel cost considering VPE obtained using EMFO reported in^48^ is incorrect. The exact value of total fuel cost is 1.3038 × 10^5^ $/hr. Also, the results of total emission obtained using QPSO-Chi2^41^, and EMFO^48^ are incorrect. The exact values of the total emission are 1.2360 × 10^5^ ton/hr, and 0.98519 × 10^5^ ton/hr for these algorithms, respectively. Therefore, this comparison reflects the superiority of the proposed EOA for minimizing the total fuel cost and emission individually as single OFs.

**Table 10:** Comparison between the single OFs using the proposed EOA and other methods for 40-unit system (Cases 1–3).

Method	Case 1	Case 2	Case 3
Proposed EOA	1.1865 × 10^5^	1.21408 × 10^5^	0.66599 × 10^5^
GWO	1.1914 × 10^5^	1.2344 × 10^5^	0.72978 × 10^5^
PSO	1.1947 × 10^5^	1.2502 × 10^5^	0.76434 × 10^5^
DEA	1.1998 × 10^5^	1.2468 × 10^5^	0.82389 × 10^5^
CCDE^52^	N/A	1.21412 × 10^5^	N/A
DPD^53,54^	N/A	1.21411 × 10^5^	N/A
MGAIPSO^19^	1.2466 × 10^5^	N/A	N/A
QOPO^24^	1.2179 × 10^5^	N/A	N/A
OCcGSA^27^	1.2141 × 10^5^	N/A	N/A
NPF + NPRS^29^	1.2141 × 10^5^	N/A	N/A
SSGO^31^	1.2141 × 10^5^	N/A	N/A
CLDE^36^	1.2159 × 10^5^	N/A	N/A
OWP-based OMF^38^	1.2131 × 10^5^	N/A	1.76682 × 10^5^
BBO^40^	1.2148 × 10^5^	N/A	-
QPSO-Chi2^41^	1.2126 × 10^5^	N/A	2.0141 × 10^5a^
ihPSODE^43^	1.2142 × 10^5^	N/A	N/A
EMFO^48^	1.2039 × 10^5^	1.21074 × 10^5b^	1.7648 × 10^5c^

N/A: Not available

^a^ The exact value of total emission is 1.2360 × 10^5^ ton/hr, which is lower than that reported in^41^.

^b^ The exact value of total fuel cost is 1.3038 × 10^5^ $/hr, which is higher than that reported in^48^.

^c^ The exact value of total emission is 0.98519 × 10^5^ ton/hr, which is lower than that reported in^48^.

Table 11 presents a comparison between the proposed EOA and other methods for minimizing the considered OFs simultaneously with and without VPE for 40-unit system. It can be observed that the total fuel cost and total emission obtained using the proposed EOA are lower than those obtained using other methods. This comparison reflects the great capability of the proposed EOA to solve the multi-OF.

**Fig. 5:**
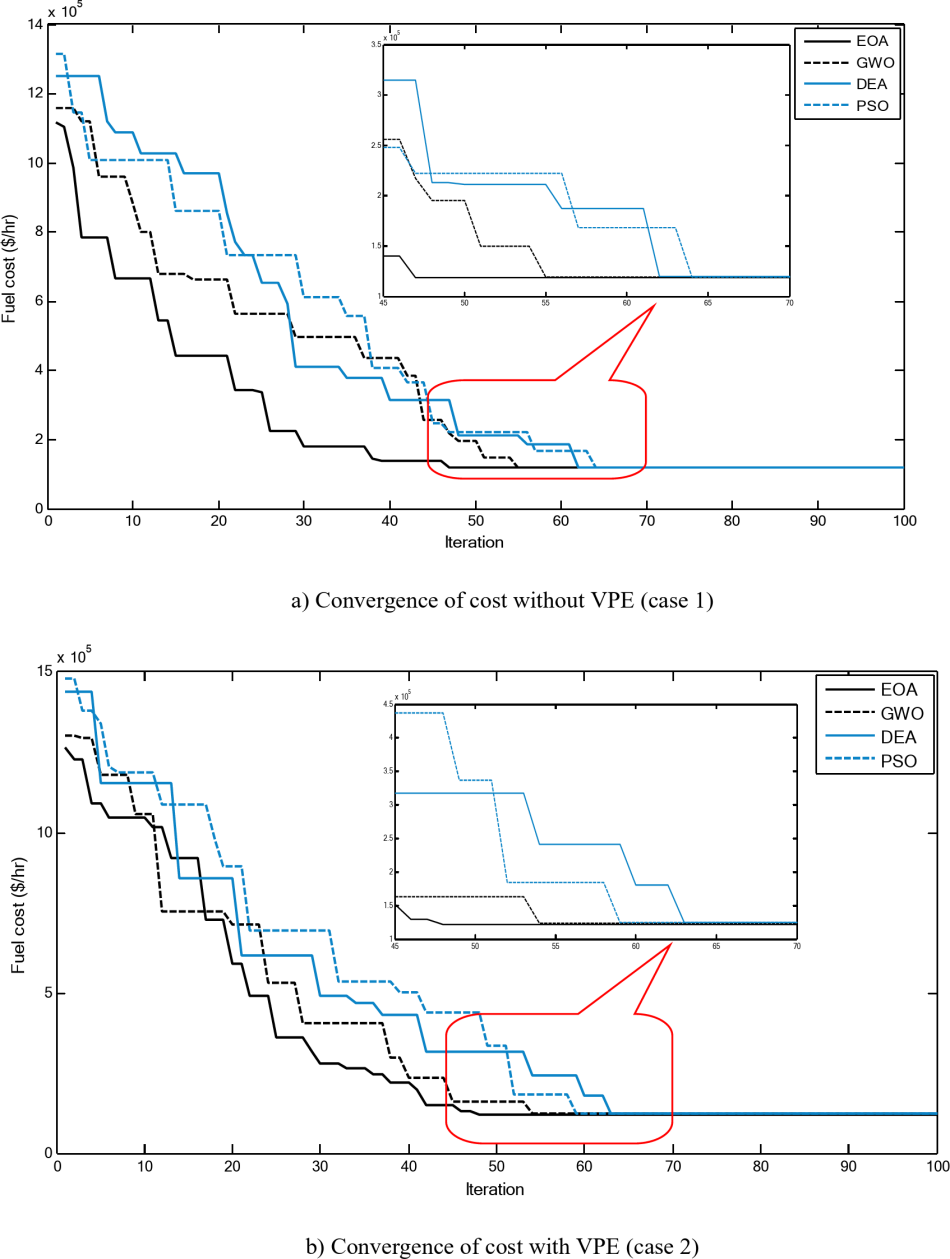

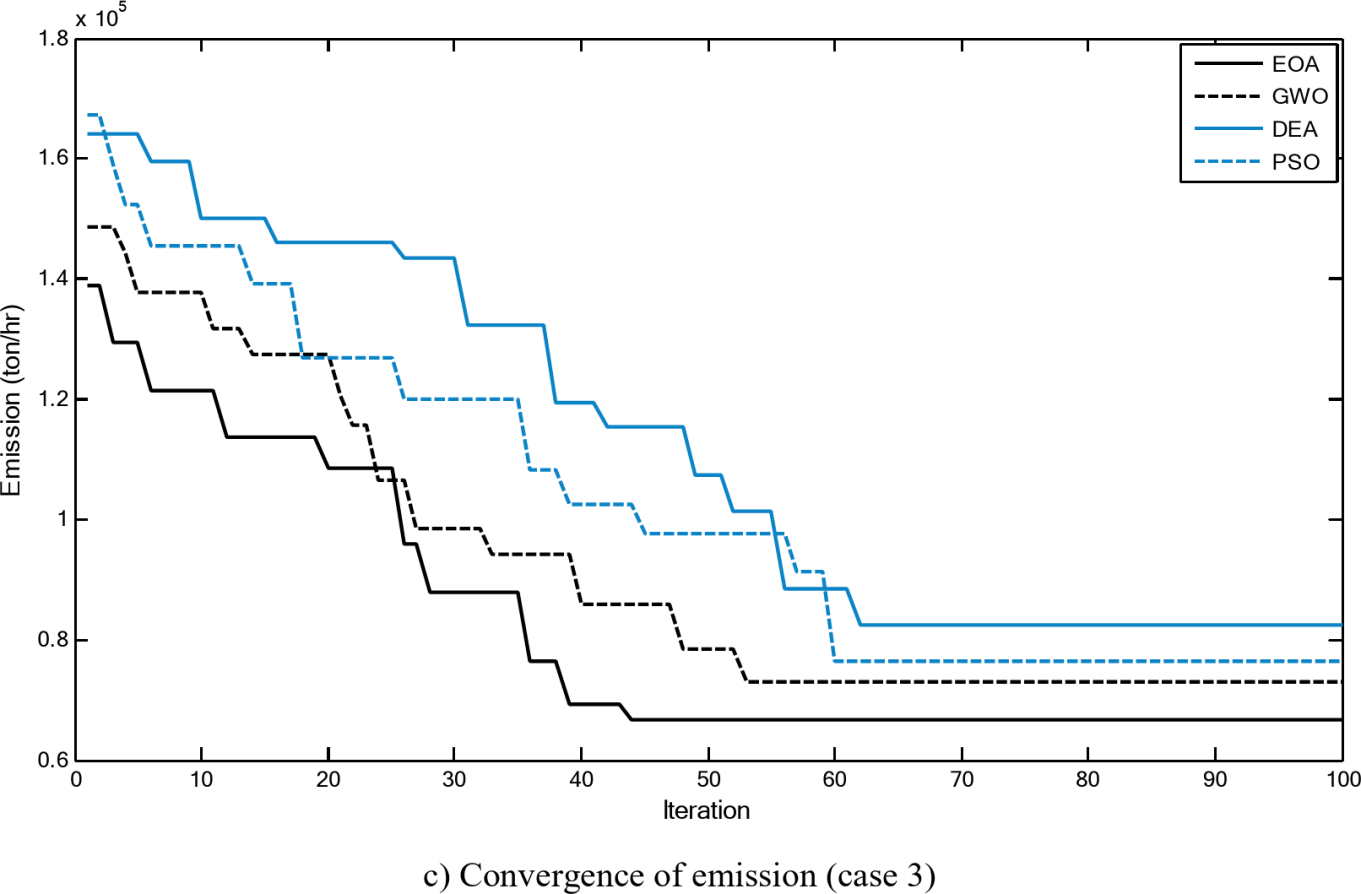
Convergence curves of the proposed EOA and other algorithms without considering power losses for 40-unit system

Figure 5 compares the convergence curves recorded by running the proposed EOA, GWO, DEA, and PSO without considering power losses for cases 1–3 for 40-unit system. From these figures, it can be observed that the proposed EOA reaches the optimal solution with a minimum number of iterations.

**Table 11:** Comparison between the multi-OF using the proposed EOA and other methods for 40-unit system (Cases 4,5).

Method	Case 4		Case 5	
	Total fuel cost ($/hr)	Total emission (ton/hr)	Total fuel cost ($/hr)	Total emission (ton/hr)
Proposed EOA	1.2492 × 10^5^	0.93335 × 10^5^	1.2838 × 10^5^	0.95291 × 10^5^
GWO	1.2598 × 10^5^	0.97028 × 10^5^	1.2989 × 10^5^	1.0305 × 10^5^
PSO	1.2615 × 10^5^	1.0456 × 10^5^	1.3334 × 10^5^	1.0501 × 10^5^
DEA	1.2719 × 10^5^	1.0991 × 10^5^	1.3529 × 10^5^	1.1230 × 10^5^
QOPO^24^	N/A	N/A	1.2954 × 10^5^	1.76886 × 10^5^
OWP-based OMF^38^	1.28595 × 10^5^	1.78557 × 10^5^	N/A	N/A
ihPSODE^43^	1.2225 × 10^5^	2.0985 × 10^5^	N/A	N/A
EMFO^48^	1.2492 × 10^5^	0.93335 × 10^5^	1.2838 × 10^5^	0.95291 × 10^5^

#### 80-unit system

Tables 12 and 13 show the optimal results obtained using the proposed EOA and other methods without considering power losses for cases 1 and 2, which aim to minimize the total fuel cost with and without considering the VPE for 80-unit system. The total cost obtained using the proposed EOA is lower than that obtained using other methods. The proposed EOA gives better solutions with maximum savings in the total fuel cost by 14,730 $/hr and 9230 $/hr with a percentage reduction of 5.8441%, and 3.6186% than the base case (results of PSO) for cases 1 and 2, respectively. Moreover, the total generation power (total PG) obtained using EMFO^48^in case 2 is higher than the total load demand, which violates the equality constraint between the total generation power and the total load. In addition, the total fuel cost obtained using EMFO^48^ in case 2 is incorrect. The exact value of total fuel cost is 2.5558 × 10^5^ $/hr. Therefore, this comparison reflects the superiority of the proposed EOA for reducing the total fuel cost with and without considering the VPE as a single OF.

Table 14presents the optimal results obtained using the proposed algorithm and other methods without considering power losses for case 3, which aims to minimize the total emission for 80-unit system. The total emission obtained using the proposed EOA is lower than that obtained using other methods. The total emission obtained using the proposed EOA is reduced by 7.5948% than the base case (results of PSO). Moreover, the total generation power (total PG) obtained using EMFO reported in^48^ is lower than the total load demand, which violates the equality constraint between the total generation power and the total load. This comparison reflects the great capability of the proposed EOA to reduce the total emission as a single OF.

**Table 12:** Simulation results using different algorithms without considering power losses for 80-unit system (Case 1).

Unit (MW)	EMFO^48^	DEA	PSO	GWO	Proposed EOA	Unit (MW)	EMFO^48^	DEA	PSO	GWO	Proposed EOA
**PG**_**1**_	114.0000	114.0000	96.8125	101.9660	113.9999	**PG**_**44**_	185.8800	142.6444	90.2758	154.4198	189.9815
**PG**_**2**_	113.2900	43.3896	99.4539	92.4340	113.9517	**PG**_**45**_	55.0000	91.9878	69.2869	91.5438	96.9997
**PG**_**3**_	109.3500	111.0642	65.1480	118.1647	119.9243	**PG**_**46**_	97.5580	72.2611	82.6704	122.1170	139.9235
**PG**_**4**_	189.1800	155.6961	170.3453	178.1466	189.4065	**PG**_**47**_	272.6500	294.1458	298.8832	293.8632	299.9995
**PG**_**5**_	90.8380	97.0000	76.2143	97.0000	97.0000	**PG**_**48**_	299.5800	243.6702	243.2733	291.3174	299.9942
**PG**_**6**_	132.1700	79.5429	140.0000	110.6830	139.9761	**PG**_**49**_	299.9200	293.8696	299.8140	298.5502	299.9998
**PG**_**7**_	299.9600	260.6922	298.8929	298.2767	299.9976	**PG**_**50**_	190.4500	212.4926	250.9279	131.5665	130.0802
**PG**_**8**_	295.8700	223.5185	254.5944	295.1637	299.9109	**PG**_**51**_	146.7500	139.1646	239.8126	126.7527	94.0106
**PG**_**9**_	300.0000	299.7529	271.6826	297.9216	299.9963	**PG**_**52**_	141.9200	210.3094	158.6992	157.3312	94.0194
**PG**_**10**_	200.6400	132.7297	212.6796	139.6435	130.0077	**PG**_**53**_	125.0400	481.4632	473.2048	149.7481	125.0001
**PG**_**11**_	103.0400	273.0569	165.4518	100.1926	94.0438	**PG**_**54**_	309.0900	385.4573	407.9992	254.1883	248.2456
**PG**_**12**_	154.5200	191.8132	319.5829	184.9170	94.0288	**PG**_**55**_	277.5700	448.5935	499.6039	363.7120	270.1128
**PG**_**13**_	199.9000	262.3375	387.0170	213.7687	126.8778	**PG**_**56**_	325.0400	360.4044	423.6766	289.5072	267.7985
**PG**_**14**_	291.1700	267.9271	483.5692	233.7271	279.0214	**PG**_**57**_	500.0000	485.4145	461.5894	483.8692	499.5468
**PG**_**15**_	190.4300	355.1339	384.6046	348.5614	250.5830	**PG**_**58**_	499.2900	420.2477	301.6736	485.1689	499.9962
**PG**_**16**_	330.0000	354.1818	463.5760	305.8937	294.6593	**PG**_**59**_	549.9900	508.7896	450.0719	528.8364	549.9793
**PG**_**17**_	499.9600	484.0204	500.0000	492.8023	499.9999	**PG**_**60**_	549.9800	550.0000	520.6422	525.7851	549.9825
**PG**_**18**_	452.6200	493.7118	499.9261	499.3153	500.0000	**PG**_**61**_	550.0000	543.6099	550.0000	545.6508	550.0000
**PG**_**19**_	549.9900	548.0229	360.1751	534.9289	549.9267	**PG**_**62**_	550.0000	525.0138	417.2986	547.2944	549.9892
**PG**_**20**_	543.8900	534.6733	549.3262	539.3469	549.9827	**PG**_**63**_	550.0000	531.0868	549.5890	548.3968	549.9965
**PG**_**21**_	550.0000	535.3074	546.4561	548.9342	549.9967	**PG**_**64**_	550.0000	550.0000	516.7856	548.0493	550.0000
**PG**_**22**_	550.0000	534.6745	547.8913	547.5282	550.0000	**PG**_**65**_	550.0000	549.7875	549.2777	546.5453	549.9972
**PG**_**23**_	550.0000	544.0075	440.2246	550.0000	549.9913	**PG**_**66**_	549.99	534.9044	525.8593	546.1994	549.9901
**PG**_**24**_	550.0000	550.0000	254.5402	544.5965	550.0000	**PG**_**67**_	10.9140	10.3231	32.3869	19.8561	10.0519
**PG**_**25**_	549.9800	547.8841	513.1178	550.0000	549.9944	**PG**_**68**_	10.4250	10.2241	18.1594	10.3814	10.2239
**PG**_**26**_	550.0000	548.5072	549.9541	550.0000	549.9934	**PG**_**69**_	12.5020	10.3247	41.7725	11.9698	10.2549
**PG**_**27**_	10.6050	10.7164	20.3977	11.8191	10.0419	**PG**_**70**_	96.3790	52.6813	97.0000	96.4156	96.9933
**PG**_**28**_	10.0000	10.6319	51.5707	11.2969	10.0061	**PG**_**71**_	190.0000	189.8871	94.6183	189.0532	189.9999
**PG**_**29**_	10.1190	11.7506	22.9361	10.8830	10.1785	**PG**_**72**_	190.0000	142.2474	190.0000	189.0217	189.9917
**PG**_**30**_	60.7560	87.8887	86.5792	88.2649	96.9829	**PG**_**73**_	189.9300	88.1918	72.7829	189.9458	189.9937
**PG**_**31**_	190.0000	183.1313	181.6685	188.8252	189.9996	**PG**_**74**_	197.8700	125.7622	195.2636	189.0541	199.9971
**PG**_**32**_	190.0000	186.0829	149.2146	189.5687	189.9990	**PG**_**75**_	200.0000	159.2427	175.5479	198.6953	199.9990
**PG**_**33**_	190.0000	119.4154	146.2689	190.0000	189.9991	**PG**_**76**_	200.0000	115.4807	200.0000	194.5141	199.9986
**PG**_**34**_	137.1700	177.2106	130.4096	197.9287	199.9997	**PG**_**77**_	108.1000	109.6512	37.2913	105.6737	109.9271
**PG**_**35**_	170.0600	161.4160	108.2394	199.6865	199.9663	**PG**_**78**_	93.3730	35.3481	85.5080	76.0142	109.9401
**PG**_**36**_	199.9900	199.4960	182.0734	199.6980	199.9700	**PG**_**79**_	63.8150	106.7374	55.4254	106.5094	109.9853
**PG**_**37**_	96.0490	109.9203	85.2623	94.8032	109.9474	**PG**_**80**_	549.9300	493.0541	533.7239	535.2555	549.9429
**PG**_**38**_	79.8770	55.1866	106.8002	102.5829	109.9984	**Total PG**	21,000	21,000	21,000	21,000	21,000
**PG**_**39**_	109.8300	90.7019	108.2583	104.3216	109.9476	**Cost ($/hr)**	2.3978 × 10^5^	2.4540 × 10^5^	2.5205 × 10^5^	2.3943 × 10^5^	**2.3732 × 10** ^**5**^
**PG**_**40**_	545.3400	492.4271	476.6022	536.9131	549.9994	**Emission (ton/hr)**	9.3960 × 10^5^	7.3599 × 10^5^	6.0421 × 10^5^	8.4005 × 10^5^	9.7608 × 10^5^
**PG**_**41**_	105.9600	114.0000	79.0482	112.2572	114.0000	**Saving ($/hr)**	**12,270**	**6650**	**Base case**	**12,620**	**14,730**
**PG**_**42**_	112.1800	106.6531	96.0556	49.4749	113.9897						
**PG**_**43**_	82.3740	116.2516	106.9834	94.9906	118.7616	**Reduction (%)**	**4.8681**	**2.6384**	**Base case**	**5.0069**	**5.8441**

**Table 13:** Simulation results using different algorithms without considering power losses for 80-unit system (Case 2).

Unit (MW)	EMFO^48^		DEA		PSO		GWO		Proposed EOA		Unit (MW)		EMFO^48^		DEA		PSO		GWO		Proposed EOA	
**PG**_**1**_	112.4040		89.3659		105.4398		112.8277		113.2405		**PG**_**44**_		179.6450		158.6995		131.3212		82.6104		179.7251	
**PG**_**2**_	113.5470		106.1395		110.8598		36.8185		113.6320		**PG**_**45**_		88.2450		92.9849		94.5424		95.2467		96.2600	
**PG**_**3**_	97.4540		70.9244		100.4905		101.5329		117.3534		**PG**_**46**_		140.0000		112.0955		135.9816		111.3025		126.3536	
**PG**_**4**_	179.7350		82.9767		175.8308		102.0443		180.9481		**PG**_**47**_		260.0000		300.0000		293.8280		293.7982		295.0925	
**PG**_**5**_	95.0010		82.5430		91.0567		94.2688		89.3905		**PG**_**48**_		285.1240		285.1009		280.2700		297.2886		286.2727	
**PG**_**6**_	140.0000		123.0285		70.8108		138.1473		139.9999		**PG**_**49**_		184.6540		216.9584		286.4417		295.4732		284.9750	
**PG**_**7**_	260.0000		247.4203		275.3933		278.0357		297.0023		**PG**_**50**_		130.0000		258.9830		238.2898		262.1847		165.0361	
**PG**_**8**_	284.6230		279.8924		265.1147		293.5406		290.2204		**PG**_**51**_		168.0010		156.2050		299.2779		315.4814		94.0069	
**PG**_**9**_	285.1750		256.6108		269.3019		292.1016		288.5580		**PG**_**52**_		94.0000		317.4918		264.7834		262.0040		98.7673	
**PG**_**10**_	130.0000		278.7514		207.7485		202.0039		130.4360		**PG**_**53**_		125.0000		390.2028		296.6578		236.1408		125.0742	
**PG**_**11**_	94.0000		320.7968		277.4434		177.0853		94.2226		**PG**_**54**_		304.5007		370.3429		485.6418		306.4326		394.3146	
**PG**_**12**_	168.5010		286.0342		94.2235		159.9513		172.0694		**PG**_**55**_		394.3250		454.0652		484.0698		394.9238		304.6167	
**PG**_**13**_	214.8450		478.1661		376.4644		303.2894		127.6572		**PG**_**56**_		394.3250		200.6246		286.1282		304.6006		394.3045	
**PG**_**14**_	394.1480		471.4515		394.6591		395.7031		394.6735		**PG**_**57**_		488.4000		483.8196		499.8185		487.7389		493.7946	
**PG**_**15**_	393.5710		473.8898		399.3584		331.2232		304.5064		**PG**_**58**_		489.0000		484.2142		233.4522		489.7039		498.5902	
**PG**_**16**_	393.6980		442.9755		481.0431		395.3663		485.1419		**PG**_**59**_		511.4570		512.7713		508.6927		513.4261		511.4442	
**PG**_**17**_	489.4424		489.2945		479.9293		499.9669		489.3442		**PG**_**60**_		511.5450		320.1484		513.4123		512.1937		511.8761	
**PG**_**18**_	489.2856		484.8351		481.0066		489.4870		489.4535		**PG**_**61**_		523.2037		527.0937		527.2740		529.6159		549.1368	
**PG**_**19**_	511.2839		513.1632		505.6326		516.7826		511.2594		**PG**_**62**_		523.4000		439.6251		436.8953		525.7344		523.2810	
**PG**_**20**_	511.3010		515.9635		512.2458		514.9375		511.4014		**PG**_**63**_		523.3120		523.6483		525.0315		523.9331		549.7494	
**PG**_**21**_	523.2835		539.2342		513.2941		534.7622		524.9422		**PG**_**64**_		523.2965		507.0438		526.9781		527.1024		549.9407	
**PG**_**22**_	523.2828		509.1249		533.2573		525.7621		523.4803		**PG**_**65**_		523.4120		523.1870		529.7070		528.1863		523.5807	
**PG**_**23**_	523.2793		545.0101		522.9027		524.2651		546.9243		**PG**_**66**_		523.3497		479.0421		525.3622		527.8549		544.5363	
**PG**_**24**_	523.2968		346.2088		527.2468		523.7527		528.7207		**PG**_**67**_		10.0000		48.6984		10.0484		30.3707		10.2043	
**PG**_**25**_	523.2236		519.3506		516.7344		548.5895		524.8906		**PG**_**68**_		110.0000		29.3739		22.6986		30.0540		10.1046	
**PG**_**26**_	523.2925		532.6313		524.6226		529.2068		524.3518		**PG**_**69**_		110.0000		31.7640		16.4543		22.9459		10.1657	
**PG**_**27**_	10.0000		42.3015		51.5443		25.1756		10.1383		**PG**_**70**_		90.0000		78.5924		83.0670		47.6394		47.0471	
**PG**_**28**_	10.0000		20.4702		14.4170		10.7804		11.0875		**PG**_**71**_		190.0000		84.3276		155.1280		172.7776		189.9438	
**PG**_**29**_	10.0000		38.2444		12.5584		17.5508		10.7987		**PG**_**72**_		190.0000		164.2021		175.6718		160.6749		187.5153	
**PG**_**30**_	88.1227		88.3966		66.9321		88.5045		47.5566		**PG**_**73**_		190.0000		119.2173		77.6981		164.8486		188.8537	
**PG**_**31**_	190.0000		187.0516		156.8056		181.5694		178.8103		**PG**_**74**_		165.3210		143.7529		197.7047		181.9391		199.8714	
**PG**_**32**_	190.0000		178.7657		91.8098		170.4224		189.8402		**PG**_**75**_		200.0000		122.6443		198.9916		157.5548		199.9746	
**PG**_**33**_	190.0000		169.7875		181.5587		161.7743		189.8025		**PG**_**76**_		200.0000		171.8222		90.0600		191.1549		168.8301	
**PG**_**34**_	165.2210		178.7120		187.7985		196.8499		199.5468		**PG**_**77**_		110.0000		76.4822		108.0456		51.1919		100.6512	
**PG**_**35**_	200.0000		108.5198		91.6313		168.3030		199.9756		**PG**_**78**_		110.0000		51.3769		99.3187		70.4360		108.9560	
**PG**_**36**_	200.0000		104.1936		95.5380		127.9498		199.9862		**PG**_**79**_		110.0000		61.9204		30.4431		50.7340		57.6241	
**PG**_**37**_	110.0000		56.2800		78.3856		87.6518		109.9995		**PG**_**80**_		512.0000		511.4786		513.8113		514.8271		511.3592	
**PG**_**38**_	110.0000		106.8644		27.6208		100.1022		105.8548		**Total PG**		21,100^a^		21,000		21,000		21,000		21,000	
**PG**_**39**_	110.0000		71.0193		98.9435		31.4686		106.7964		**Cost ($/hr)**		2.4290 × 10^5b^		2.6017 × 10^5^		2.5507 × 10^5^		2.5132 × 10^5^		**2.4584 × 10** ^**5**^	
**PG**_**40**_	511.3210		511.1673		526.4975		512.3710		549.7587		**Emission (ton/hr)**		5.7274 × 10^5^		5.1196 × 10^5^		5.5321 × 10^5^		5.9572 × 10^5^		7.0120 × 10^5^	
**PG**_**41**_	114.0000		104.1299		112.9712		72.0998		110.7939		**Saving ($/hr)**		**−510** ^c^		**−5100**		**Base case**		**3750**		**9230**	
**PG**_**42**_	110.6470		62.1963		104.8138		53.5703		113.4035													
**PG**_**43**_	97.5000		76.1157		105.0644		102.2779		60.1997		**Reduction (%)**		**−0.1999**		**−1.9995**		**Base case**		**1.4702**		**3.6186**	

**Table 14:** Simulation results using different algorithms without considering power losses for 80-unit system (Case 3).

Unit (MW)	EMFO [48]	DEA	PSO	GWO	Proposed EOA	Unit (MW)	EMFO [48]	DEA	PSO	GWO	Proposed EOA
**PG**_**1**_	113.9997	110.2970	112.1485	113.1385	113.5706	**PG**_**44**_	159.3282	159.1969	155.5548	159.3568	159.0287
**PG**_**2**_	113.7379	51.8145	57.0270	113.8443	112.7485	**PG**_**45**_	73.3021	96.9449	96.9454	96.9219	96.8908
**PG**_**3**_	81.8947	113.9193	110.1596	109.3393	115.1716	**PG**_**46**_	114.3446	116.1541	118.6395	118.8667	117.4027
**PG**_**4**_	158.8768	165.7169	166.3235	163.1179	157.8143	**PG**_**47**_	286.6427	289.6340	288.0365	281.7306	282.5537
**PG**_**5**_	71.6054	96.8707	93.2085	96.9835	96.9779	**PG**_**48**_	272.2790	283.1730	280.4214	285.6532	282.8328
**PG**_**6**_	120.5910	115.7009	116.0652	118.7030	116.9402	**PG**_**49**_	287.0221	286.2560	288.9237	279.5051	280.6837
**PG**_**7**_	280.9490	283.9009	287.1061	281.5728	279.9768	**PG**_**50**_	290.4878	278.8000	283.2435	280.4507	278.6537
**PG**_**8**_	276.3689	288.7859	283.2251	281.8725	280.7595	**PG**_**51**_	285.6921	286.2908	285.6123	285.5782	281.7676
**PG**_**9**_	284.1128	286.8839	287.1293	278.1073	282.5433	**PG**_**52**_	287.0221	291.1168	287.2562	281.7206	281.5702
**PG**_**10**_	279.9949	286.1772	285.9724	282.4611	281.8382	**PG**_**53**_	419.6174	424.5270	417.0075	415.5022	409.8640
**PG**_**11**_	282.6289	291.9360	286.9668	282.7989	277.7188	**PG**_**54**_	415.6176	417.6484	420.6554	414.9795	412.2371
**PG**_**12**_	285.6582	282.3164	286.7357	282.1513	281.6427	**PG**_**55**_	415.6001	422.0331	421.4560	414.7654	409.5663
**PG**_**13**_	415.0397	428.5065	420.4635	414.6502	408.9334	**PG**_**56**_	417.7571	424.8659	416.8518	418.2616	413.1109
**PG**_**14**_	417.7272	427.7946	419.6376	420.6800	414.7790	**PG**_**57**_	418.5315	427.2277	419.9998	412.5427	413.1169
**PG**_**15**_	414.7869	421.7760	415.4620	413.7775	411.9540	**PG**_**58**_	415.5713	428.0526	417.7303	417.2261	413.4947
**PG**_**16**_	418.9748	423.5266	421.8994	416.8381	411.0786	**PG**_**59**_	419.5352	426.2538	418.6094	416.3873	411.0216
**PG**_**17**_	412.8375	426.1694	414.5125	417.0664	413.7513	**PG**_**60**_	415.9485	422.2463	418.1872	417.4766	414.6536
**PG**_**18**_	414.8997	419.9010	419.1983	414.9546	411.6530	**PG**_**61**_	418.0022	425.3447	419.2741	415.6722	412.1630
**PG**_**19**_	413.9892	425.0554	416.0978	416.4911	414.3970	**PG**_**62**_	417.7792	418.4665	417.5276	421.9410	415.1245
**PG**_**20**_	412.0937	423.8134	417.2706	417.3928	413.5686	**PG**_**63**_	414.5960	426.2831	418.9424	416.8983	412.4575
**PG**_**21**_	418.8459	424.7448	413.5340	422.4156	410.4951	**PG**_**64**_	413.3588	421.4370	423.0159	420.5178	415.9058
**PG**_**22**_	415.5026	422.8152	417.0406	413.9385	413.8680	**PG**_**65**_	418.5347	421.8019	421.3292	413.9466	412.9376
**PG**_**23**_	414.3629	423.7007	415.7098	414.2604	415.0477	**PG**_**66**_	420.4356	425.6717	417.4492	417.0259	414.3344
**PG**_**24**_	417.0097	419.3052	420.6775	419.8311	412.6926	**PG**_**67**_	150.0000	150.0000	150.0000	150.0000	149.9995
**PG**_**25**_	415.9926	428.5574	416.9730	414.3525	412.2540	**PG**_**68**_	150.0000	149.8047	149.9538	149.8996	149.9803
**PG**_**26**_	418.4022	426.5862	419.0847	413.1672	411.9117	**PG**_**69**_	150.0000	149.9914	149.5355	110.0543	149.9532
**PG**_**27**_	149.9999	11.0613	149.9431	149.9985	149.9996	**PG**_**70**_	93.38114	56.2314	97.0000	67.7998	96.9520
**PG**_**28**_	149.9999	149.8726	149.9506	149.9993	149.9967	**PG**_**71**_	153.7112	162.0672	161.9116	158.4876	158.9887
**PG**_**29**_	149.9999	149.7503	18.0051	150.0000	149.9929	**PG**_**72**_	161.6624	152.1103	161.0759	165.2014	157.8164
**PG**_**30**_	66.9288	96.8775	96.8676	96.7726	96.9976	**PG**_**73**_	158.2236	165.7228	160.5131	159.0977	161.0183
**PG**_**31**_	158.3165	169.6049	157.5837	159.9210	159.0326	**PG**_**74**_	200.0000	200.0000	200.0000	200.0000	199.9584
**PG**_**32**_	160.0855	156.1794	161.1795	159.4450	158.4369	**PG**_**75**_	199.9988	199.8843	199.8842	199.9558	199.9998
**PG**_**33**_	157.9581	165.3769	160.5010	161.4917	158.9920	**PG**_**76**_	199.9999	199.9615	200.0000	199.8908	199.9895
**PG**_**34**_	199.9999	199.8516	199.8789	199.8660	199.9978	**PG**_**77**_	82.1612	92.4157	96.1611	45.3161	95.2093
**PG**_**35**_	199.9999	200.0000	199.9381	199.9677	199.9994	**PG**_**78**_	97.2357	47.3985	94.9579	90.6902	94.3910
**PG**_**36**_	200.0000	199.9255	199.9901	199.9987	199.9782	**PG**_**79**_	93.2120	95.4706	68.6682	96.1562	92.4730
**PG**_**37**_	96.9403	99.2325	96.3267	99.0918	96.3593	**PG**_**80**_	418.7578	425.1603	415.2624	415.5193	414.0448
**PG**_**38**_	91.1856	45.5257	91.3748	78.7325	94.3693	**Total PG**	20,999^a^	21,000	21,000	21,000	21,000
**PG**_**39**_	90.3385	96.9430	82.9594	99.0058	91.1194	**Cost ($/hr)**	3.1386 × 10^5^	3.0370 × 10^5^	3.0315 × 10^5^	3.0853 × 10^5^	3.1326 × 10^5^
**PG**_**40**_	415.4988	430.6617	419.1389	418.3397	415.5158	**Emission (ton/hr)**	1.3977 × 10^5^	1.5562 × 10^5^	1.4431 × 10^5^	1.3957 × 10^5^	**1.3335 × 10** ^**5**^
**PG**_**41**_	113.9784	84.3031	113.9191	112.6050	113.6767	**Reduction (%)**	**3.1460**	**−7.8373**	**Base case**	**3.2846**	**7.5948**
**PG**_**42**_	113.7000	55.3192	112.5472	105.1433	113.1337						
**PG**_**43**_	108.0896	107.2979	112.6744	114.7192	116.1697						

Tables 15 and 16 show the optimal results obtained using the proposed EOA and other methods without considering power losses for cases 4 and 5 to minimize the total fuel cost and total emission for 80-unit system. The proposed EOA gives better results than other methods by reducing the total cost and total emission by 6.7355% and 15.4641% for case 4, and 5.4103% and 14.2918% for case 5 than the base case (results of PSO). In addition, the proposed EOA gives maximum savings in the total fuel cost by 18,470 $/hr and 15,270 $/hr than the base case (results of PSO) for cases 4 and 5, respectively. Therefore, this comparison reflects the superiority of the proposed EOA for minimizing total cost and emission as a multi-OF.

**Table 15:** Simulation results of multi-OF using different algorithms without considering power losses for 80-unit system (Case 4).

Unit (MW)	EMFO^48^	DEA	PSO	GWO	Proposed EOA	Unit (MW)	EMFO^48^	DEA	PSO	GWO	Proposed EOA
**PG**_**1**_	113.9999	67.4591	100.8674	108.7224	113.8998	**PG**_**44**_	170.3741	169.8984	176.8705	168.1865	164.3946
**PG**_**2**_	113.6499	113.5963	39.8535	50.5658	113.9978	**PG**_**45**_	74.9192	79.4191	68.0666	80.9908	96.8997
**PG**_**3**_	112.3172	118.3032	114.9513	107.1558	119.9869	**PG**_**46**_	120.8659	129.3672	84.3359	122.4044	121.0678
**PG**_**4**_	150.6439	171.1722	161.0815	172.3984	164.3464	**PG**_**47**_	279.0513	297.7686	293.4455	293.1210	291.2999
**PG**_**5**_	76.3727	96.6180	96.6965	66.7150	96.8976	**PG**_**48**_	279.5596	292.2101	296.8428	296.7112	291.3208
**PG**_**6**_	122.4982	125.5646	124.8154	124.9644	120.9732	**PG**_**49**_	292.2765	297.8519	286.0777	292.4923	291.2624
**PG**_**7**_	292.1958	290.0891	295.6006	296.9416	291.2397	**PG**_**50**_	277.0594	293.0281	281.2081	285.7705	285.1517
**PG**_**8**_	279.3376	290.5592	292.7275	291.7096	291.3177	**PG**_**51**_	287.2516	293.9235	295.4411	294.1225	286.6919
**PG**_**9**_	293.1292	299.6611	293.0605	295.7079	291.2442	**PG**_**52**_	286.3244	285.4356	282.9650	301.4271	286.6612
**PG**_**10**_	290.9995	294.4940	291.7477	284.2077	285.2137	**PG**_**53**_	426.3932	447.9563	441.3068	413.8745	420.8847
**PG**_**11**_	289.8689	283.9739	295.0747	300.8135	286.8473	**PG**_**54**_	424.7795	433.9709	436.6181	427.5390	421.8540
**PG**_**12**_	291.0160	296.9648	314.8349	287.9074	286.5372	**PG**_**55**_	429.9879	438.5726	441.6750	431.4321	421.8821
**PG**_**13**_	428.0288	432.8556	440.0791	426.6614	420.9685	**PG**_**56**_	424.3595	418.6438	433.1353	429.7174	421.9317
**PG**_**14**_	422.0941	410.3082	445.9897	430.9075	421.8101	**PG**_**57**_	433.7129	420.8476	435.3932	432.5731	426.8693
**PG**_**15**_	428.8010	421.2428	420.0022	428.5070	421.9403	**PG**_**58**_	424.5211	439.2636	453.2683	434.0192	426.8114
**PG**_**16**_	427.8846	436.4318	427.1392	428.6908	421.8143	**PG**_**59**_	429.1863	446.3557	435.8464	434.3317	426.7803
**PG**_**17**_	430.5259	435.9814	431.3254	433.1684	426.8103	**PG**_**60**_	430.5255	421.1622	431.2466	433.9153	426.7326
**PG**_**18**_	421.2732	430.2636	448.9249	432.5117	426.8044	**PG**_**61**_	431.6077	431.6574	431.2759	428.2463	428.2240
**PG**_**19**_	426.7053	427.8717	436.8352	430.2692	426.8382	**PG**_**62**_	425.7248	426.9738	442.7410	441.2845	428.3135
**PG**_**20**_	430.6994	440.7731	442.7985	433.2114	426.7518	**PG**_**63**_	433.9700	434.6412	456.4590	428.0524	428.3120
**PG**_**21**_	433.7768	447.5018	431.9755	424.9403	428.1365	**PG**_**64**_	431.9074	412.2893	441.9907	424.4331	428.2925
**PG**_**22**_	434.7359	430.8631	445.1384	431.8403	428.2807	**PG**_**65**_	435.7249	436.0869	441.4996	426.8888	428.6066
**PG**_**23**_	436.9140	443.3633	446.7755	428.9394	428.4194	**PG**_**66**_	436.1900	435.7069	445.6163	423.7348	427.9432
**PG**_**24**_	435.2508	436.1451	457.9882	433.9120	428.3475	**PG**_**67**_	112.9281	83.4224	72.4968	25.4354	51.9788
**PG**_**25**_	429.6569	433.8865	449.4263	440.2708	427.9570	**PG**_**68**_	86.4045	124.1792	79.0028	92.6000	49.8483
**PG**_**26**_	435.1648	415.7085	447.9381	436.8173	427.9670	**PG**_**69**_	68.2295	88.6566	113.9679	68.2413	53.7828
**PG**_**27**_	75.8447	86.2716	105.3368	67.6055	52.6826	**PG**_**70**_	75.2533	61.1235	80.5304	96.8466	96.9879
**PG**_**28**_	101.9397	125.8613	58.3110	51.9695	50.1040	**PG**_**71**_	153.4069	160.5272	63.5344	172.2997	166.2617
**PG**_**29**_	67.4971	101.5711	105.2896	99.8852	53.8155	**PG**_**72**_	171.7399	179.1179	157.2035	166.4096	166.3498
**PG**_**30**_	95.9052	96.4674	73.8388	94.7035	96.7968	**PG**_**73**_	167.3577	177.9908	153.0595	173.5308	166.3663
**PG**_**31**_	166.6852	114.3654	163.9341	170.7891	166.2837	**PG**_**74**_	200.0000	93.8752	198.6686	199.9228	199.9998
**PG**_**32**_	167.9954	177.8766	90.6899	169.9657	166.3019	**PG**_**75**_	199.9999	197.3421	198.9910	199.8925	199.9996
**PG**_**33**_	172.2518	69.6378	138.7257	146.1738	166.3313	**PG**_**76**_	199.9999	199.8823	151.8669	199.5288	199.8879
**PG**_**34**_	143.3759	198.4553	180.5342	199.7957	199.9997	**PG**_**77**_	102.8862	33.5933	43.5937	102.9930	98.5592
**PG**_**35**_	199.9999	199.6723	199.1748	199.4157	199.9989	**PG**_**78**_	57.4447	102.2698	70.7375	29.8546	98.5204
**PG**_**36**_	199.9989	198.7544	179.8596	199.8013	199.9999	**PG**_**79**_	81.7053	49.8814	92.3148	106.5989	98.5196
**PG**_**37**_	81.0314	100.8227	83.8946	46.4022	98.4766	**PG**_**80**_	431.4677	437.8965	433.1062	429.7675	426.8193
**PG**_**38**_	101.4899	83.9416	52.8861	100.7592	98.5184	**Total PG**	21,000	21,000	21,000	21,000	21,000
**PG**_**39**_	98.5705	99.9607	103.7420	88.7644	98.5499	**Cost ($/hr)**	2.7037 × 10^5^	2.8023 × 10^5^	2.7422 × 10^5^	2.6392 × 10^5^	**2.5575 × 10** ^**5**^
**PG**_**40**_	429.5575	425.6464	432.4394	428.8428	426.7259	**Emission (ton/hr)**	1.6997 × 10^5^	1.8364 × 10^5^	1.9639 × 10^5^	1.7903 × 10^5^	**1.6602 × 10** ^**5**^
**PG**_**41**_	92.6124	113.6355	112.8789	71.9106	113.9998	**Saving ($/hr)**	**3850**	**−6010**	**Base case**	**10,300**	**18,470**
**PG**_**42**_	73.5692	50.3912	78.0743	111.6158	113.9988						
**PG**_**43**_	89.0342	92.2278	104.3431	113.9530	119.9995	**Reduction in cost and emission (%)**	**1.4040**,** 13.4528**	**−2.1917**,** 6.4922**	**Base case**	**3.7561**, **8.8396**	**6.7355**, **15.4641**

**Table 16:** Simulation results of multi-OF using different algorithms without considering power losses for 80-unit system (Case 5).

Unit (MW)	EMFO^48^	DEA	PSO	GWO	Proposed EOA	Unit (MW)	EMFO^48^	DEA	PSO	GWO	Proposed EOA
**PG**_**1**_	113.9998	111.9122	112.3477	114.0000	113.9240	**PG**_**44**_	171.8888	169.0788	162.5775	167.1338	165.6596
**PG**_**2**_	112.9843	38.2656	57.9626	113.9973	113.9999	**PG**_**45**_	92.3995	96.9366	95.4932	96.8141	96.9997
**PG**_**3**_	119.8879	115.9177	60.2918	107.0631	119.8826	**PG**_**46**_	122.1456	122.0774	127.9529	122.5810	120.0716
**PG**_**4**_	166.4089	87.0294	168.9971	169.8160	166.2238	**PG**_**47**_	286.2974	295.0995	300.0000	296.7667	291.5360
**PG**_**5**_	92.0297	49.3522	96.3804	96.0334	96.9998	**PG**_**48**_	288.7699	296.1310	297.6912	292.1747	286.2114
**PG**_**6**_	116.3944	130.9601	118.4543	124.2109	119.9342	**PG**_**49**_	295.0196	289.4068	295.7636	286.6322	285.6110
**PG**_**7**_	285.1152	285.5421	294.4729	294.5134	293.9495	**PG**_**50**_	281.45	285.4961	284.4760	284.5349	280.9938
**PG**_**8**_	293.4157	290.4423	298.1156	284.6660	289.3677	**PG**_**51**_	284.1616	302.8287	296.7632	290.1057	289.0418
**PG**_**9**_	285.6267	295.9597	290.8963	291.2391	287.7185	**PG**_**52**_	287.2793	300.8277	295.1168	292.8005	290.0762
**PG**_**10**_	286.4371	288.2347	293.9833	282.4503	280.6118	**PG**_**53**_	418.8579	413.7348	437.8185	413.9026	415.1819
**PG**_**11**_	293.1005	96.9993	286.3448	291.2224	288.7392	**PG**_**54**_	426.7212	431.0948	430.4956	415.1524	416.8607
**PG**_**12**_	289.5353	294.7395	300.0137	292.5213	290.3888	**PG**_**55**_	418.5805	415.1755	430.3179	424.4697	414.4936
**PG**_**13**_	420.9094	435.3847	430.3493	414.8215	414.3233	**PG**_**56**_	421.8241	437.3575	438.2787	424.4321	415.4646
**PG**_**14**_	421.3507	420.5524	442.4081	422.6416	415.2647	**PG**_**57**_	418.7913	444.4473	430.2158	422.7036	420.3379
**PG**_**15**_	417.9764	421.6697	433.7745	417.4171	418.4216	**PG**_**58**_	420.9101	427.2335	437.5927	428.4985	419.7959
**PG**_**16**_	417.9396	432.0765	422.3698	419.0320	415.4530	**PG**_**59**_	424.1026	428.1520	437.5816	423.7496	421.5196
**PG**_**17**_	420.9704	433.7910	433.2018	422.6201	419.2550	**PG**_**60**_	422.9145	429.3454	434.8650	421.7366	421.5196
**PG**_**18**_	427.1639	432.9530	437.6341	429.5626	419.6631	**PG**_**61**_	432.4987	439.9712	432.8475	433.5189	433.5193
**PG**_**19**_	422.3246	430.0507	429.0041	422.8057	421.5196	**PG**_**62**_	433.4056	434.5863	428.8547	433.5542	433.5196
**PG**_**20**_	421.5259	430.5149	428.3668	422.0895	421.5196	**PG**_**63**_	432.9913	436.6250	434.7761	433.5192	433.5195
**PG**_**21**_	433.5191	434.3612	437.0418	433.5267	433.5194	**PG**_**64**_	433.3517	430.5871	435.3815	433.4409	433.5195
**PG**_**22**_	431.0021	435.1671	339.3776	433.0268	433.5194	**PG**_**65**_	433.4732	435.4915	436.7599	433.4187	433.5261
**PG**_**23**_	432.6235	432.0027	435.3182	433.5026	433.5193	**PG**_**66**_	431.6136	430.9100	435.8338	433.3664	433.5175
**PG**_**24**_	433.3824	434.4944	433.4555	433.1360	433.5191	**PG**_**67**_	105.5689	74.7850	70.6575	103.4766	56.6788
**PG**_**25**_	433.5187	432.7619	441.7734	433.4201	433.5190	**PG**_**68**_	77.1213	96.4898	99.5014	77.3357	69.3123
**PG**_**26**_	431.5518	436.1086	435.5314	433.5020	433.5193	**PG**_**69**_	96.0653	131.8596	127.7946	57.1346	67.0721
**PG**_**27**_	52.5079	93.2986	91.7207	38.8370	57.8760	**PG**_**70**_	68.5411	61.7063	96.8830	97.0000	96.9989
**PG**_**28**_	94.8193	87.8200	10.3601	56.7025	69.9622	**PG**_**71**_	162.3134	168.7560	178.5991	167.7753	162.5699
**PG**_**29**_	79.2247	124.0403	93.7759	108.5122	71.5699	**PG**_**72**_	160.3917	178.1747	174.4735	161.8283	162.6904
**PG**_**30**_	96.9895	80.2409	97.0000	96.9626	96.9999	**PG**_**73**_	167.1915	166.2438	176.2157	169.2512	163.5822
**PG**_**31**_	164.6412	156.9477	174.5576	164.7804	162.6714	**PG**_**74**_	198.3389	200.0000	199.9462	199.9974	199.9987
**PG**_**32**_	160.9021	160.3148	63.5673	164.5616	162.8752	**PG**_**75**_	199.9999	200.0000	90.1725	199.9866	199.9996
**PG**_**33**_	167.4437	172.6740	167.9796	166.1761	162.1577	**PG**_**76**_	172.2766	199.9918	200.0000	199.9985	199.9988
**PG**_**34**_	199.9999	199.7784	199.8879	199.9986	199.9988	**PG**_**77**_	99.7431	66.9177	105.6932	100.8089	97.7018
**PG**_**35**_	199.9999	199.9441	200.0000	200.0000	199.9999	**PG**_**78**_	69.2547	63.8045	105.8148	101.5720	97.4880
**PG**_**36**_	199.9999	198.7641	198.6478	199.9933	199.9996	**PG**_**79**_	101.5341	98.9975	103.6147	26.5719	96.6474
**PG**_**37**_	55.4923	105.8028	101.8867	43.8752	98.9311	**PG**_**80**_	422.6939	431.1776	435.7589	421.6219	421.5196
**PG**_**38**_	98.1265	99.0309	70.1459	78.2611	95.7300	**Total PG**	21,000	21,000	21,000	21,000	21,000
**PG**_**39**_	69.8698	96.5002	66.0015	103.4558	98.7873	**Cost ($/hr)**	2.7665 × 10^5^	2.8713 × 10^5^	2.8224 × 10^5^	2.7309 × 10^5^	**2.6697 × 10** ^**5**^
**PG**_**40**_	421.5194	432.9539	437.9079	422.4914	421.5196	**Emission (ton/hr)**	1.6293 × 10^5^	1.7961 × 10^5^	1.8864 × 10^5^	1.6662 × 10^5^	**1.6168 × 10** ^**5**^
**PG**_**41**_	111.6184	101.1462	109.9011	113.8148	113.9991	**Saving ($/hr)**	**5590**	**−4890**	**Base case**	**9150**	**15,270**
**PG**_**42**_	113.9912	112.5090	36.2265	113.1075	113.9997						
**PG**_**43**_	111.4607	119.4917	119.9878	106.2665	119.8925	**Reduction in cost and emission (%)**	**1.9806**,** 13.6291**	**−1.7326**,** 4.7869**	**Base case**	**3.2419**, **11.6730**	**5.4103**,** 14.2918**

Table 17shows a comparison between the proposed EOA and other methods when the considered OFs are minimized individually for 80-unit system. It can be observed that the proposed EOA gives minimum values of the OFs than obtained using other methods for all cases. Moreover, the total fuel cost considering VPE obtained using EMFO reported in^48^ is incorrect. The exact value of total fuel cost is 2.5558 × 10^5^ $/hr. Therefore, this comparison reflects the superiority of the proposed EOA for minimizing the total fuel cost and emission individually as single OFs.

**Table 17:** Comparison between different single OFs using the proposed EOA and other methods for 80-unit system (Cases 1–3).

Method	Case 1	Case 2	Case 3
Proposed EOA	2.3732 × 10^5^	2.4584 × 10^5^	1.3335 × 10^5^
GWO	2.3943 × 10^5^	2.5132 × 10^5^	1.3957 × 10^5^
PSO	2.5205 × 10^5^	2.5507 × 10^5^	1.4431 × 10^5^
DEA	2.4540 × 10^5^	2.6017 × 10^5^	1.5562 × 10^5^
MPSO_SSM^20^	2.4286 × 10^5^	N/A	N/A
SSGO^31^	2.4279 × 10^5^	N/A	N/A
EMFO^48^	2.3978 × 10^5^	2.4290 × 10^5a^	1.3977 × 10^5^

N/A: Not available.

^a^ The exact value of total fuel cost is 2.5558 × 10^5^ $/hr, which is higher than that reported in^48^.

Table 18 presents a comparison between the proposed EOA and other methods for minimizing the OFs simultaneously with and without VPE for 80-unit system. It can be noticed that the total fuel cost and total emission obtained using the proposed EOA are lower than those obtained using other methods for all cases. This comparison reflects the great capability of the proposed EOA to solve the multi-OF.

**Table 18:** Comparison between the multi-OF using the proposed EOA and other methods for 80-unit system (Cases 4,5).

Method	Case 4		Case 5	
	Total fuel cost ($/hr)	Total emission (ton/hr)	Total fuel cost ($/hr)	Total emission (ton/hr)
Proposed EOA	2.5575 × 10^5^	1.6602 × 10^5^	2.6697 × 10^5^	1.6168 × 10^5^
GWO	2.6392 × 10^5^	1.7903 × 10^5^	2.7309 × 10^5^	1.6662 × 10^5^
PSO	2.7422 × 10^5^	1.9639 × 10^5^	2.8224 × 10^5^	1.8864 × 10^5^
DEA	2.8023 × 10^5^	1.8364 × 10^5^	2.8713 × 10^5^	1.7961 × 10^5^
EMFO^48^	2.7037 × 10^5^	1.6997 × 10^5^	2.7665 × 10^5^	1.6293 × 10^5^

Figure 5 compares the convergence curves recorded by running the proposed EOA, GWO, DEA, and PSO without considering power losses for cases 1–3 for 80-unit system. The proposed EOA reaches the optimal solution with a minimum number of iterations compared with other methods.

#### Large-scale power system: 140-unit system

Tables 19 and 20 show the optimal results obtained using the proposed EOA and other methods without considering power losses for cases 6 and 7, which aim to minimize the total fuel cost with and without considering the VPE, RRL, and POZs for 140-unit system. The total cost obtained using the proposed EOA is lower than that obtained using other methods. The proposed EOA gives better solutions with maximum savings in the total fuel cost by 107,200 $/hr and 126,400 $/hr with a percentage reduction of 6.4203%, and 7.2394% than the base case (results of PSO) for cases 6 and 7, respectively. This comparison reflects the superiority of the proposed EOA for reducing the total fuel cost with and without considering the VPE, RRL, and POZs for large-scale power systems.

Table 21 presents the optimal results obtained using the proposed algorithm and other methods without considering power losses for case 8, which aims to minimize the total emission for 140-unit system. The total emission obtained using the proposed EOA is lower than that obtained using other methods. The total emission obtained using the proposed EOA is reduced by 2.5688% than the base case (results of PSO). This comparison reflects the great capability of the proposed EOA to reduce the total emission for large-scale power systems.

**Table 19:** Simulation results using different algorithms without considering power losses for 140-unit system (Case 6).

Unit (MW)	DEA	PSO	GWO	Proposed EOA	Unit (MW)	DEA	PSO	GWO	Proposed EOA	Unit (MW)	DEA	PSO	GWO	Proposed EOA
P_G1_	79.495	117.623	76.192	110.211	P_G51_	278.571	167.936	369.578	165.521	P_G101_	951.465	958.000	952.276	958.000
P_G2_	144.515	135.412	133.541	188.989	P_G52_	295.763	240.953	363.819	202.323	P_G102_	989.960	1007.000	1006.149	1007.000
P_G3_	156.484	187.239	127.627	189.989	P_G53_	271.066	176.309	433.181	243.607	P_G103_	994.615	1006.000	1005.085	1005.985
P_G4_	183.010	143.357	183.507	189.843	P_G54_	175.351	186.524	191.580	231.741	P_G104_	991.721	990.668	1013.000	1012.989
P_G5_	184.400	168.099	110.935	145.871	P_G55_	312.078	266.387	322.287	181.294	P_G105_	1018.500	1016.997	1013.831	1019.981
P_G6_	106.286	155.202	100.685	188.288	P_G56_	184.457	199.665	266.273	181.240	P_G106_	952.532	946.889	954.000	953.959
P_G7_	484.866	485.470	484.074	490.000	P_G57_	173.950	337.875	247.162	104.761	P_G107_	940.564	870.939	952.000	951.987
P_G8_	438.753	490.000	478.260	489.972	P_G58_	372.479	222.226	200.166	198.416	P_G108_	1003.070	996.871	998.299	1005.999
P_G9_	453.698	471.190	493.966	495.985	P_G59_	164.366	100.910	119.733	311.988	P_G109_	1003.022	1013.000	1010.038	1012.995
P_G10_	380.978	486.693	496.000	495.948	P_G60_	320.745	437.311	168.586	285.772	P_G110_	1010.917	1011.824	1020.133	1020.989
P_G11_	496.000	485.975	485.503	495.996	P_G61_	212.234	176.399	249.693	163.557	P_G111_	1005.830	1015.000	1011.936	1014.995
P_G12_	470.914	492.902	461.634	496.000	P_G62_	144.596	162.849	228.390	95.638	P_G112_	130.266	193.723	123.658	95.336
P_G13_	498.652	451.435	502.014	505.971	P_G63_	471.308	234.506	338.641	217.852	P_G113_	106.603	119.259	114.792	95.272
P_G14_	349.637	314.625	477.471	509.000	P_G64_	186.215	163.778	160.927	163.143	P_G114_	100.602	158.185	100.840	94.000
P_G15_	451.558	481.016	431.097	506.000	P_G65_	261.409	196.570	220.020	207.297	P_G115_	270.247	249.872	278.466	244.879
P_G16_	404.556	488.763	465.157	504.975	P_G66_	247.621	285.348	199.671	196.390	P_G116_	244.552	263.214	245.697	245.624
P_G17_	487.343	395.477	503.453	505.999	P_G67_	315.102	481.670	355.980	489.831	P_G117_	249.743	244.000	290.452	244.002
P_G18_	399.539	502.566	504.617	506.000	P_G68_	309.250	443.510	392.665	478.244	P_G118_	162.226	151.710	122.490	95.999
P_G19_	488.777	406.690	463.079	504.996	P_G69_	327.425	390.808	139.937	131.215	P_G119_	130.163	104.300	101.152	95.012
P_G20_	463.902	489.789	470.700	504.987	P_G70_	134.465	130.173	291.094	296.692	P_G120_	121.684	155.923	126.421	116.018
P_G21_	478.492	501.645	505.000	504.983	P_G71_	252.550	236.303	183.505	149.731	P_G121_	184.268	175.223	259.413	182.810
P_G22_	405.197	492.116	501.935	504.987	P_G72_	280.241	150.896	215.946	428.160	P_G122_	10.707	17.921	17.494	2.042
P_G23_	496.570	499.735	410.502	505.000	P_G73_	261.483	201.931	441.644	233.885	P_G123_	4.229	7.601	10.325	4.084
P_G24_	458.785	500.538	497.525	505.000	P_G74_	325.852	517.240	258.091	175.007	P_G124_	16.199	39.943	36.643	15.242
P_G25_	495.279	523.068	471.537	536.937	P_G75_	450.389	231.595	190.554	175.546	P_G125_	35.207	14.722	31.146	9.078
P_G26_	515.562	507.675	521.144	537.000	P_G76_	233.310	175.104	210.376	219.141	P_G126_	18.686	36.191	15.402	19.192
P_G27_	529.260	549.000	414.792	548.955	P_G77_	381.425	288.047	285.643	208.730	P_G127_	32.392	23.582	19.052	10.001
P_G28_	448.082	524.537	534.105	549.000	P_G78_	335.223	339.432	358.015	368.213	P_G128_	241.675	188.196	153.243	112.037
P_G29_	484.241	464.905	500.322	500.987	P_G79_	492.299	318.525	471.789	530.980	P_G129_	12.083	4.037	6.589	5.306
P_G30_	449.909	471.376	498.385	501.000	P_G80_	512.803	488.537	387.874	530.873	P_G130_	21.039	12.199	10.987	5.047
P_G31_	500.025	495.763	500.677	505.899	P_G81_	397.815	320.526	451.438	262.456	P_G131_	5.781	5.221	14.180	7.191
P_G32_	497.129	501.185	491.490	505.995	P_G82_	66.344	57.895	56.451	56.174	P_G132_	57.824	83.986	77.010	52.886
P_G33_	445.166	487.522	491.298	505.999	P_G83_	187.939	125.173	124.495	115.519	P_G133_	7.608	10.000	8.603	5.062
P_G34_	478.056	470.205	504.631	506.000	P_G84_	124.414	118.169	159.861	115.134	P_G134_	52.575	42.405	48.801	44.458
P_G35_	486.647	484.589	492.486	499.957	P_G85_	116.986	130.223	117.105	116.339	P_G135_	47.944	45.451	43.982	42.403
P_G36_	496.617	462.150	490.373	499.718	P_G86_	258.428	208.383	214.232	207.351	P_G136_	42.849	41.412	43.882	41.003
P_G37_	195.424	239.275	225.850	241.000	P_G87_	207.455	273.324	216.804	207.000	P_G137_	32.010	31.392	18.365	17.000
P_G38_	226.421	201.826	213.919	240.999	P_G88_	253.325	246.720	175.340	186.613	P_G138_	10.958	18.697	14.868	7.179
P_G39_	737.220	763.631	748.177	774.000	P_G89_	195.922	338.824	199.633	175.130	P_G139_	14.018	12.714	12.646	8.572
P_G40_	769.000	765.617	762.852	768.965	P_G90_	210.924	247.713	187.070	199.148	P_G140_	29.638	39.212	27.855	26.575
P_G41_	5.621	14.905	3.966	18.945	P_G91_	207.904	298.918	196.444	177.498	**Total PG**	49,342	49,342	49,342	49,342
P_G42_	9.182	14.331	15.200	3.178	P_G92_	547.309	576.750	572.288	580.000					
P_G43_	170.380	224.522	214.979	243.789	P_G93_	637.991	640.468	645.000	645.000	**Cost ($/hr)**	**1.6823 × 10** ^**6**^	**1.6697 × 10** ^**6**^	**1.6384 × 10** ^**6**^	**1.5625 × 10** ^**6**^
P_G44_	214.076	165.035	184.536	183.576	P_G94_	984.000	976.974	983.453	983.989	**Emission (ton/hr)**	792.0269	839.5413	800.2984	867.0233
P_G45_	192.610	162.820	160.789	247.581	P_G95_	940.390	978.000	975.910	978.000					
P_G46_	181.708	162.473	195.876	244.081	P_G96_	669.480	669.848	676.349	682.000	**Saving** **($/hr)**	**−12,600**	**Base case**	**31,300**	**107,200**
P_G47_	190.602	201.290	206.790	160.000	P_G97_	699.414	672.562	718.020	719.986					
P_G48_	212.235	160.942	166.519	249.685	P_G99_	718.000	684.476	714.587	718.000	**Reduction (%)**	**−0.7546**	**Base case**	**1.8746**	**6.4203**
P_G49_	174.394	214.287	162.908	246.280	P_G99_	714.962	720.000	716.232	720.000					
P_G50_	237.773	248.116	165.185	193.186	P_G100_	957.967	959.660	964.000	963.985					

**Table 20:** Simulation results using different algorithms considering VPE, RRL, and POZs for 140-unit system (Case 7).

Unit (MW)	DEA	PSO	GWO	Proposed EOA	Unit (MW)	DEA	PSO	GWO	Proposed EOA	Unit (MW)	DEA	PSO	GWO	Proposed EOA
P_G1_	116.417	101.111	90.123	85.453	P_G51_	415.094	383.724	244.299	338.915	P_G101_	881.744	853.155	927.839	956.368
P_G2_	125.491	140.213	156.468	171.600	P_G52_	433.307	297.216	381.397	239.397	P_G102_	969.827	1000.304	1007.000	1007.000
P_G3_	149.015	128.043	155.486	186.231	P_G53_	346.501	297.854	165.000	242.645	P_G103_	987.830	998.250	982.686	1002.207
P_G4_	157.192	141.022	139.536	150.304	P_G54_	263.651	384.837	274.607	174.449	P_G104_	1013.000	927.752	951.090	1013.000
P_G5_	172.478	133.801	119.019	116.637	P_G55_	249.419	297.745	191.133	180.000	P_G105_	950.928	1015.302	837.130	1020.000
P_G6_	137.171	129.590	141.190	168.878	P_G56_	201.770	224.124	195.836	205.625	P_G106_	834.089	940.727	949.201	924.214
P_G7_	433.785	445.844	489.329	488.757	P_G57_	261.681	248.310	177.893	183.248	P_G107_	910.987	942.444	854.376	951.843
P_G8_	456.052	484.752	476.634	481.533	P_G58_	425.135	314.250	294.729	274.875	P_G108_	1003.326	900.825	998.929	997.726
P_G9_	489.140	493.306	495.343	468.144	P_G59_	250.787	221.763	169.833	258.043	P_G109_	945.943	869.509	986.306	1013.000
P_G10_	422.155	484.852	491.021	496.000	P_G60_	247.907	255.086	254.892	203.102	P_G110_	1013.575	1010.996	1017.538	1017.401
P_G11_	492.524	272.765	494.793	475.492	P_G61_	368.308	197.204	171.313	186.464	P_G111_	1007.397	811.927	894.363	1015.000
P_G12_	494.136	478.009	456.212	496.000	P_G62_	176.284	113.080	188.151	181.673	P_G112_	104.754	134.833	158.102	96.067
P_G13_	479.941	503.291	499.634	497.843	P_G63_	247.575	434.367	302.045	219.602	P_G113_	124.902	153.040	138.071	105.250
P_G14_	509.000	406.186	494.847	508.242	P_G64_	359.155	278.715	190.265	198.933	P_G114_	147.762	123.021	117.875	107.498
P_G15_	348.710	506.000	489.751	506.000	P_G65_	273.513	317.582	294.700	210.360	P_G115_	315.633	374.845	274.747	253.541
P_G16_	380.410	470.869	492.405	502.170	P_G66_	269.718	305.101	289.402	296.093	P_G116_	314.528	276.744	316.760	301.451
P_G17_	379.723	497.767	430.936	502.900	P_G67_	332.515	328.018	363.741	379.717	P_G117_	289.703	270.367	303.366	256.440
P_G18_	437.822	495.091	477.999	504.716	P_G68_	267.758	373.161	306.240	196.766	P_G118_	143.419	126.868	103.787	115.055
P_G19_	443.973	494.203	475.897	495.483	P_G69_	144.616	283.280	211.286	171.224	P_G119_	145.635	136.561	99.755	113.764
P_G20_	395.615	494.576	388.265	489.071	P_G70_	287.790	266.955	233.384	235.985	P_G120_	144.900	163.139	116.366	136.788
P_G21_	453.510	453.000	479.822	504.310	P_G71_	211.075	390.287	232.443	143.018	P_G121_	280.875	175.805	226.417	218.052
P_G22_	420.882	505.000	494.390	505.000	P_G72_	141.039	244.164	355.200	258.443	P_G122_	10.087	10.833	14.134	15.327
P_G23_	502.482	463.579	505.000	503.539	P_G73_	386.321	199.123	317.981	197.309	P_G123_	33.901	38.042	30.138	22.589
P_G24_	454.918	483.385	490.045	503.765	P_G74_	346.599	177.565	288.943	298.376	P_G124_	32.192	23.801	50.720	37.473
P_G25_	471.575	480.318	523.004	531.576	P_G75_	185.799	182.188	183.398	179.676	P_G125_	22.213	11.201	45.494	38.468
P_G26_	466.012	497.968	498.916	533.023	P_G76_	367.465	452.816	300.191	216.321	P_G126_	25.042	23.579	23.420	19.919
P_G27_	515.071	545.393	542.024	543.191	P_G77_	374.264	228.909	176.202	280.180	P_G127_	25.357	20.835	21.227	14.333
P_G28_	547.536	497.121	530.580	542.977	P_G78_	479.106	403.984	336.892	395.843	P_G128_	248.624	169.060	205.950	112.220
P_G29_	483.413	436.006	489.345	491.695	P_G79_	499.493	509.145	425.615	520.443	P_G129_	16.868	13.914	8.118	11.176
P_G30_	447.398	473.112	493.625	500.499	P_G80_	345.458	484.076	496.760	523.028	P_G130_	27.503	22.018	22.989	18.903
P_G31_	475.763	500.053	499.993	452.749	P_G81_	208.711	412.312	379.827	271.717	P_G131_	5.000	5.661	12.460	17.573
P_G32_	488.128	506.000	490.313	499.724	P_G82_	86.433	56.000	80.931	58.809	P_G132_	76.878	80.132	82.195	69.766
P_G33_	438.883	506.000	494.155	499.021	P_G83_	115.996	191.531	123.789	141.410	P_G133_	8.687	7.749	9.260	7.670
P_G34_	382.486	505.359	502.702	496.460	P_G84_	139.414	175.950	138.816	115.397	P_G134_	51.288	57.538	52.142	63.248
P_G35_	468.777	374.372	493.815	470.002	P_G85_	171.546	128.428	142.087	139.725	P_G135_	51.488	69.201	61.523	56.767
P_G36_	422.086	333.785	478.348	500.000	P_G86_	244.693	207.000	242.146	207.380	P_G136_	47.826	74.221	46.580	42.715
P_G37_	219.894	170.696	206.764	224.121	P_G87_	253.080	233.178	238.187	238.495	P_G137_	22.449	20.031	26.074	29.269
P_G38_	238.563	185.522	223.766	219.679	P_G88_	234.620	245.337	309.659	308.048	P_G138_	17.592	12.124	14.790	17.274
P_G39_	708.967	771.274	712.251	774.000	P_G89_	228.915	230.842	265.152	216.437	P_G139_	16.509	17.545	13.391	12.702
P_G40_	741.244	748.106	719.561	741.035	P_G90_	236.055	275.526	269.416	252.069	P_G140_	35.854	35.211	32.262	32.901
P_G41_	17.572	11.193	10.154	7.127	P_G91_	247.799	180.368	231.848	239.063	**Total PG**	49,342	49,342	49,342	49,342
P_G42_	13.873	17.976	13.455	10.240	P_G92_	567.845	567.595	580.000	580.000					
P_G43_	198.246	172.170	220.780	187.816	P_G93_	536.072	541.199	573.960	565.248	**Cost ($/hr)**	**1.7544 × 10** ^**6**^	**1.7460 × 10** ^**6**^	**1.6871 × 10** ^**6**^	**1.6196 × 10** ^**6**^
P_G44_	189.826	195.993	240.613	230.147	P_G94_	895.124	896.201	955.078	975.251	**Emission (ton/hr)**	760.4216	722.5878	772.8414	808.8675
P_G45_	203.341	186.102	215.482	210.502	P_G95_	978.000	912.617	978.000	961.640					
P_G46_	173.832	224.430	196.251	218.953	P_G96_	681.185	609.666	681.737	678.253	**Saving** **($/hr)**	**−8400**	**Base case**	**58,900**	**126,400**
P_G47_	182.390	180.170	226.800	184.792	P_G97_	715.783	716.436	718.900	686.529					
P_G48_	193.273	235.152	170.538	232.939	P_G99_	717.295	711.415	709.407	718.000	**Reduction (%)**	**−0.4811**	**Base case**	**3.3734**	**7.2394**
P_G49_	232.137	231.342	180.622	208.496	P_G99_	706.853	704.450	715.781	719.009					
P_G50_	185.778	176.488	220.201	160.000	P_G100_	945.761	963.848	940.732	938.973					

**Table 21:** Simulation results using different algorithms for total emission reduction for 140-unit system (Case 8).

Unit (MW)	DEA	PSO	GWO	Proposed EOA	Unit (MW)	DEA	PSO	GWO	Proposed EOA	Unit (MW)	DEA	PSO	GWO	Proposed EOA
P_G1_	116.846	99.869	102.939	114.739	P_G51_	504.000	474.505	271.716	467.439	P_G101_	880.237	821.805	852.425	946.431
P_G2_	140.388	133.356	175.728	147.442	P_G52_	395.644	382.764	414.022	499.751	P_G102_	937.067	915.001	1001.289	767.727
P_G3_	185.024	145.711	181.155	185.700	P_G53_	470.988	234.054	490.959	493.390	P_G103_	901.456	922.307	886.614	861.466
P_G4_	140.006	172.290	179.272	127.017	P_G54_	400.872	263.105	487.377	318.464	P_G104_	999.350	926.782	887.390	987.737
P_G5_	182.157	173.860	184.261	172.512	P_G55_	449.245	391.618	471.000	259.439	P_G105_	1016.986	896.116	1014.316	736.221
P_G6_	98.606	173.310	169.259	91.330	P_G56_	231.680	549.177	475.497	268.965	P_G106_	823.321	931.787	809.541	935.842
P_G7_	443.904	490.000	480.644	406.061	P_G57_	335.172	253.561	223.705	336.546	P_G107_	786.000	786.000	786.000	786.000
P_G8_	439.446	337.091	463.263	292.753	P_G58_	612.198	602.038	560.724	608.213	P_G108_	795.000	795.000	795.000	795.000
P_G9_	348.935	488.993	313.433	486.511	P_G59_	100.000	299.219	207.919	304.365	P_G109_	795.000	795.000	795.000	795.000
P_G10_	280.334	263.019	263.255	260.001	P_G60_	400.859	274.858	195.934	454.680	P_G110_	795.700	795.985	795.855	795.073
P_G11_	440.277	492.865	487.563	336.071	P_G61_	315.190	445.773	495.672	483.454	P_G111_	847.337	959.517	991.288	1007.637
P_G12_	335.056	458.380	291.389	324.632	P_G62_	108.556	279.455	291.661	286.041	P_G112_	194.944	154.257	111.029	198.025
P_G13_	477.675	485.256	496.361	500.697	P_G63_	438.583	254.799	510.386	499.334	P_G113_	182.606	100.258	130.795	193.335
P_G14_	268.402	378.064	492.489	293.185	P_G64_	496.685	348.683	252.117	509.548	P_G114_	128.579	157.226	128.113	128.895
P_G15_	473.606	308.177	438.818	404.088	P_G65_	476.526	434.813	405.272	488.817	P_G115_	364.118	329.393	366.665	244.818
P_G16_	298.226	405.859	360.451	261.229	P_G66_	308.762	255.905	488.619	482.531	P_G116_	379.000	300.702	354.809	372.905
P_G17_	495.023	480.422	396.450	438.381	P_G67_	196.423	196.000	196.000	196.000	P_G117_	253.718	255.476	333.367	244.958
P_G18_	297.808	486.344	503.647	473.963	P_G68_	198.956	196.062	196.000	196.010	P_G118_	117.870	190.000	151.989	185.467
P_G19_	395.780	416.557	286.641	504.341	P_G69_	130.000	130.791	130.000	130.056	P_G119_	101.966	145.098	153.251	109.698
P_G20_	439.015	375.580	497.034	502.131	P_G70_	394.298	285.892	206.674	174.958	P_G120_	137.726	132.399	145.957	192.308
P_G21_	482.186	386.826	489.452	499.817	P_G71_	280.123	221.929	223.693	454.576	P_G121_	269.137	211.679	293.009	201.659
P_G22_	505.000	403.143	484.525	504.572	P_G72_	453.895	362.091	312.022	445.289	P_G122_	14.685	3.017	3.343	3.408
P_G23_	499.427	436.569	496.076	499.454	P_G73_	487.330	531.449	509.080	534.834	P_G123_	20.454	28.911	23.396	11.321
P_G24_	498.720	496.410	472.132	503.153	P_G74_	355.043	527.587	467.329	527.914	P_G124_	62.122	65.721	40.372	77.989
P_G25_	537.000	536.628	530.579	487.872	P_G75_	492.108	337.255	516.453	537.746	P_G125_	16.984	13.058	40.000	9.005
P_G26_	501.529	531.233	524.708	528.783	P_G76_	175.741	489.231	502.308	527.730	P_G126_	35.620	21.440	32.739	12.537
P_G27_	280.000	280.000	280.000	280.000	P_G77_	241.177	517.163	347.482	177.014	P_G127_	21.934	12.929	15.149	27.021
P_G28_	280.000	280.000	280.000	280.000	P_G78_	426.249	423.791	344.020	330.131	P_G128_	288.895	284.456	305.214	362.343
P_G29_	260.000	260.000	260.000	260.000	P_G79_	375.966	380.999	300.624	176.171	P_G129_	11.238	4.675	5.293	14.890
P_G30_	361.254	368.402	309.417	276.962	P_G80_	523.355	452.963	397.814	527.884	P_G130_	28.191	18.805	34.467	37.976
P_G31_	443.193	470.617	478.965	506.000	P_G81_	512.649	496.807	387.047	224.934	P_G131_	5.021	12.125	16.874	10.444
P_G32_	506.000	502.197	492.880	446.174	P_G82_	87.725	65.357	116.598	129.211	P_G132_	89.144	62.810	84.387	97.324
P_G33_	506.000	437.936	366.937	505.497	P_G83_	236.224	221.054	167.102	235.823	P_G133_	8.384	8.112	5.581	5.432
P_G34_	444.327	506.000	418.512	504.562	P_G84_	215.939	212.368	241.822	241.346	P_G134_	62.603	70.884	43.363	54.995
P_G35_	497.958	494.676	361.236	494.929	P_G85_	230.112	150.542	174.259	180.803	P_G135_	57.717	55.057	44.080	73.506
P_G36_	319.099	487.031	496.429	484.332	P_G86_	307.000	302.642	232.758	210.596	P_G136_	41.568	76.840	62.947	103.534
P_G37_	235.301	189.455	120.836	154.028	P_G87_	303.693	268.563	234.765	300.895	P_G137_	42.257	50.215	30.810	48.170
P_G38_	200.639	131.032	183.960	131.571	P_G88_	254.997	295.626	247.028	343.473	P_G138_	11.074	11.479	7.738	15.515
P_G39_	713.447	562.762	554.129	423.615	P_G89_	187.383	301.743	329.403	297.547	P_G139_	8.350	13.473	14.326	13.364
P_G40_	619.272	759.939	760.651	769.000	P_G90_	212.403	263.138	199.432	175.172	P_G140_	39.105	26.422	36.607	35.634
P_G41_	10.324	7.541	4.986	3.0780	P_G91_	334.976	266.236	314.050	339.396	**Total PG**	49,342	49,342	49,342	49,342
P_G42_	10.273	12.824	7.463	11.385	P_G92_	506.104	407.414	540.011	527.882					
P_G43_	229.290	239.134	228.305	168.781	P_G93_	616.751	579.460	629.126	510.583	**Cost ($/hr)**	1.9292 × 10^6^	1.9184 × 10^6^	1.9353 × 10^6^	1.9886 × 10^6^
P_G44_	208.922	211.169	215.657	218.980	P_G94_	838.310	900.411	802.511	795.009	**Emission (ton/hr)**	**468.3059**	**469.0625**	**464.9570**	**457.0131**
P_G45_	203.330	165.321	190.337	186.724	P_G95_	804.861	910.180	809.497	795.000					
P_G46_	160.000	191.234	238.515	161.458	P_G96_	605.033	636.483	599.498	578.001	**Reduction (%)**	**0.1613**	**Base case**	**0.8753**	**2.5688**
P_G47_	232.570	178.362	207.372	245.928	P_G97_	715.237	698.599	665.361	617.660					
P_G48_	245.784	209.783	241.159	246.974	P_G99_	713.258	718.000	659.451	622.899					
P_G49_	233.153	235.407	187.940	163.637	P_G99_	657.337	677.844	687.111	715.604					
P_G50_	184.808	194.384	190.140	163.215	P_G100_	948.595	878.766	945.352	868.997					

Table 22shows a comparison between the proposed EOA and other methods when the considered OFs are minimized individually for 140-unit system as a large-scale power system. The proposed EOA gives minimum values of the OFs than other methods for all cases. Moreover, the results of total fuel cost without considering VPE, RRL, and POZs obtained using MPSO_SSM^20^, and MOMSA^22^ are incorrect. The exact values of total fuel cost are 2.0717 × 10^6^ $/hr and 1.8740 × 10^6^ $/hr for these algorithms, respectively. Therefore, this comparison reflects the superiority of the proposed EOA for minimizing the total fuel cost and total emission individually as single OFs for large-scale power systems.

**Table 22:** Comparison between different single OFs using the proposed EOA and other methods for 140-unit system (Cases 6, 7, 8).

Method	Case 6	Case 7	Case 8
Proposed EOA	1.5625 × 10^6^	1.6196 × 10^6^	457.0131
GWO	1.6384 × 10^6^	1.6871 × 10^6^	464.9570
PSO	1.6697 × 10^6^	1.7460 × 10^6^	469.0625
DEA	1.6823 × 10^6^	1.7544 × 10^6^	468.3059
MPSO_SSM^20^	1.5598 × 10^6a^	N/A	N/A
MOMSA^22^	1.629093 × 10^6b^	N/A	55970.185 lb/hr
NPF + NPRS^29^	N/A	1.55971 × 10^6^	N/A
CLDE^36^	1.65796 × 10^6^	N/A	N/A
IPSO [72]	1.657962 × 10^6^	N/A	N/A
CCDE^52^	N/A	1.657963 × 10^6^	N/A

N/A: Not available

^a^ The exact value of total fuel cost is 2.0717 × 10^6^ $/hr, which is higher than that reported in [20].

^b^ The exact value of total fuel cost is 1.8740 × 10^6^ $/hr, which is higher than that reported in [20].

**Fig. 6:**
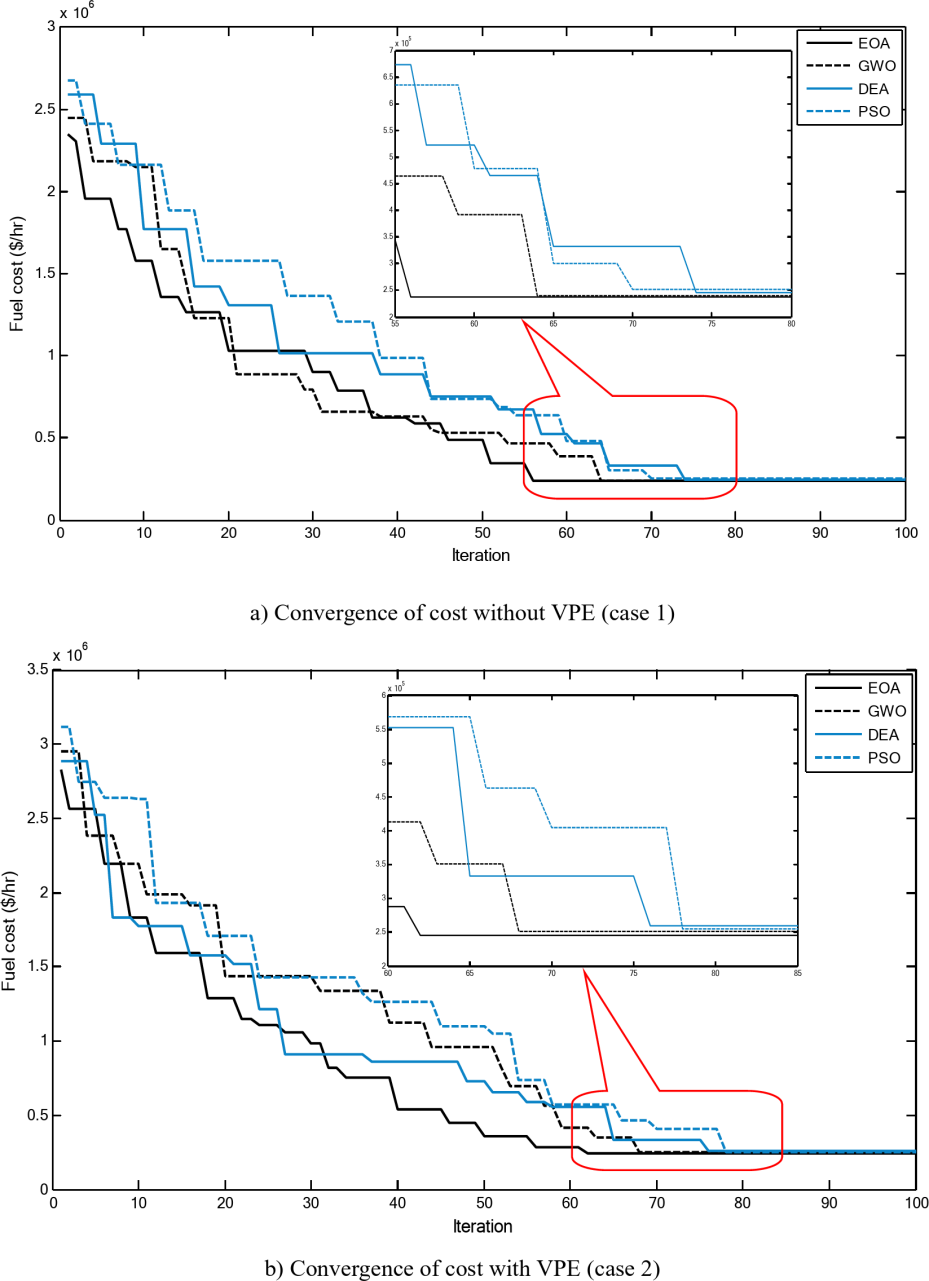

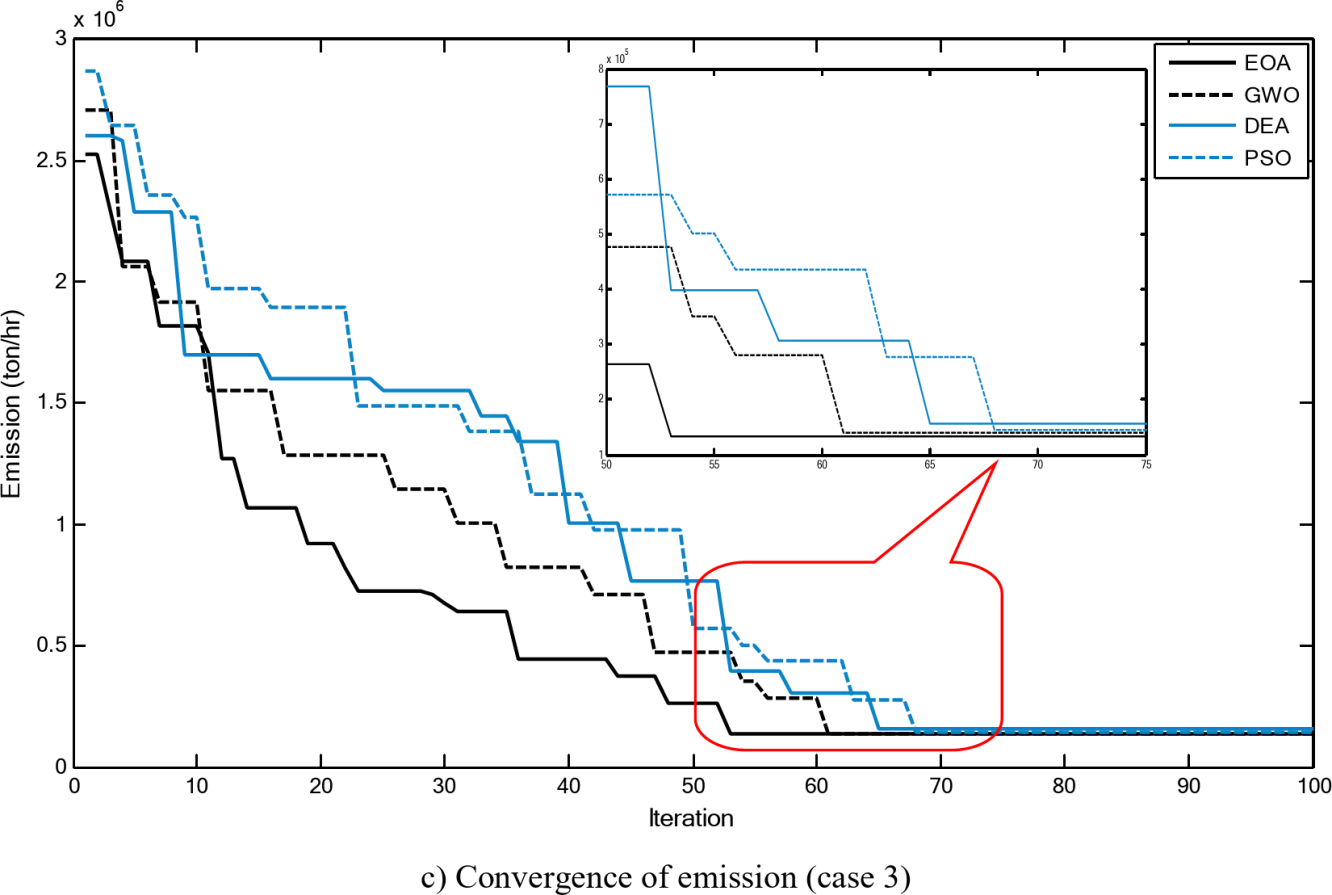
Convergence curves of the proposed EOA and other algorithms without considering power losses for 80-unit system

**Fig. 7:**
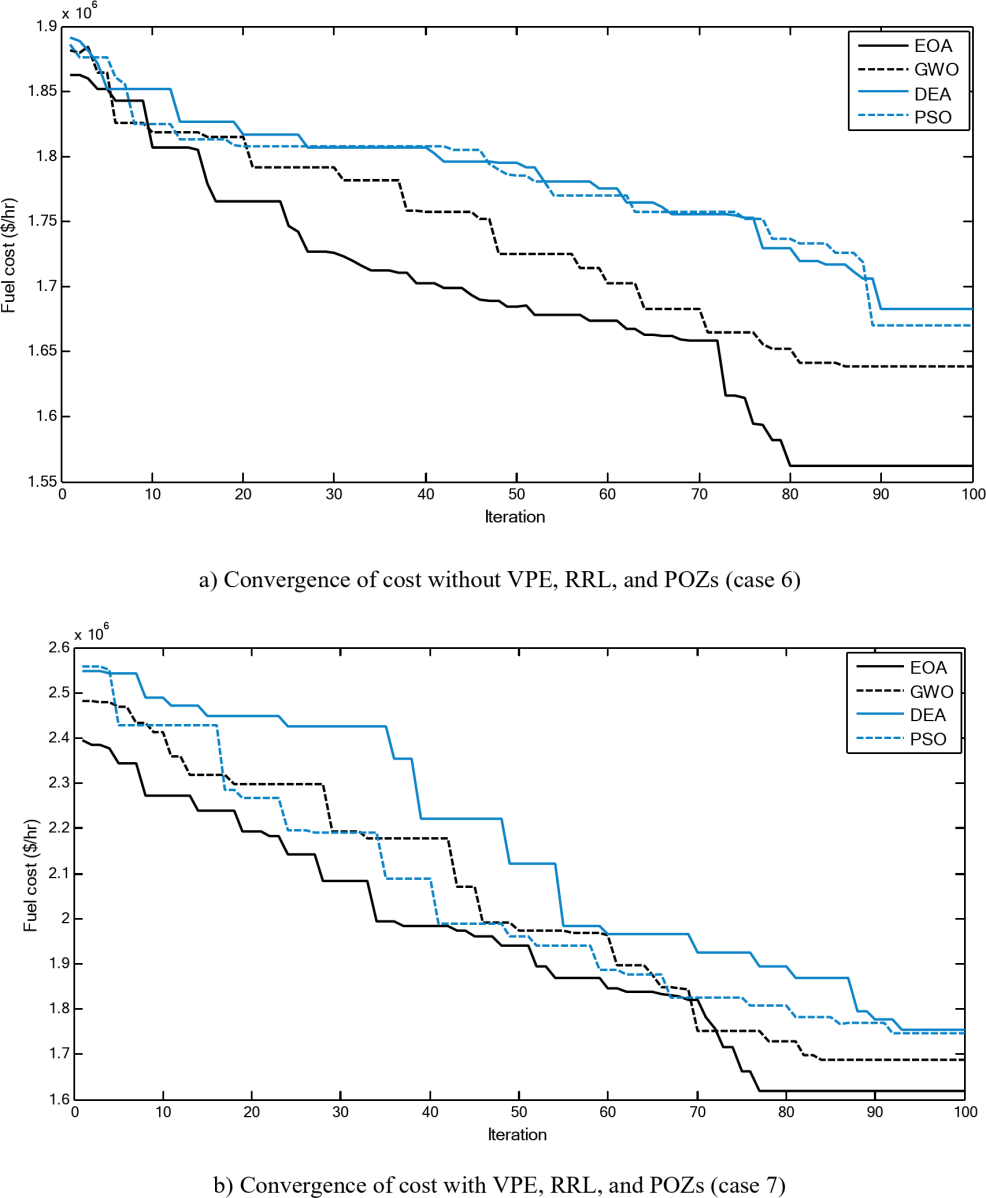

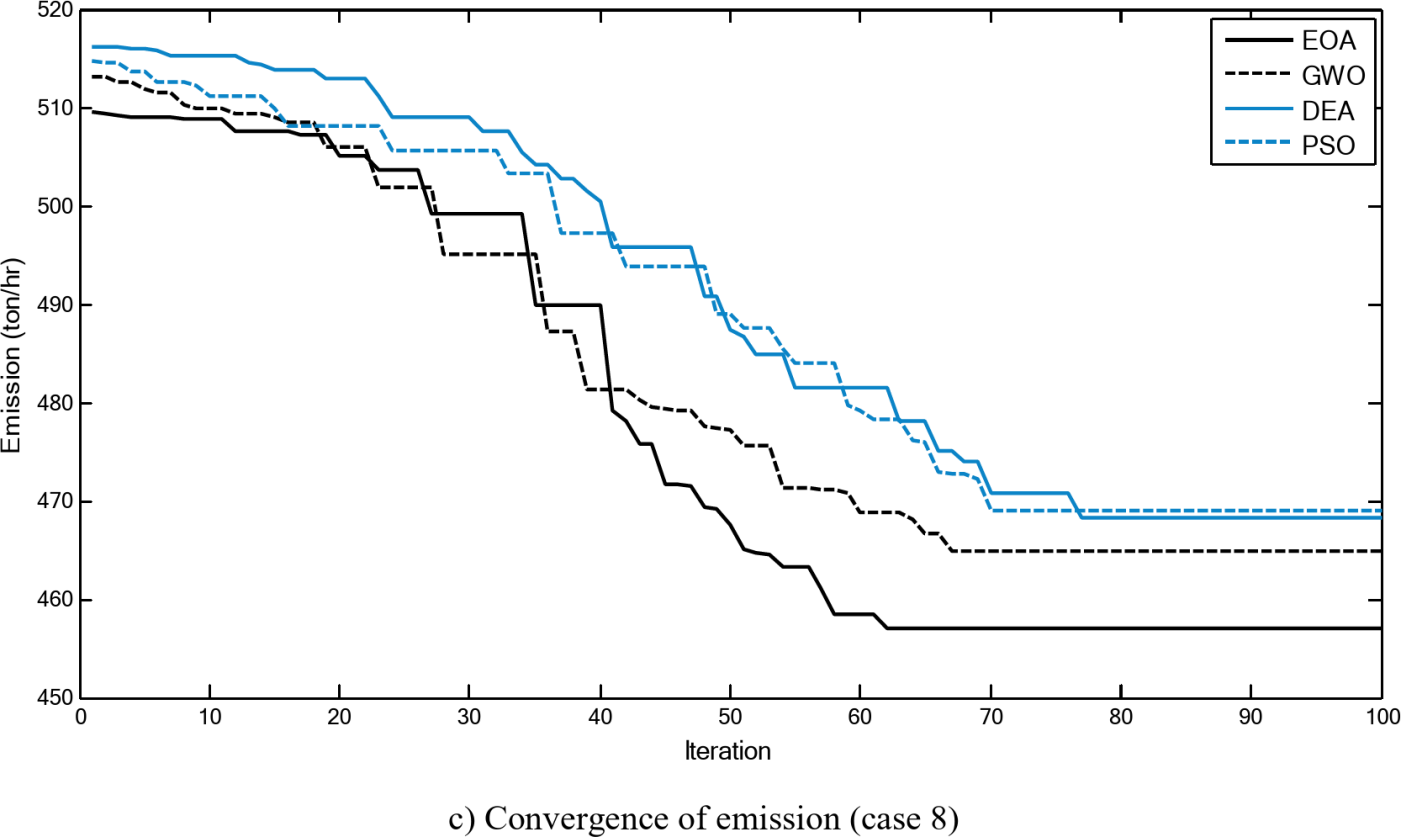
Convergence curves of the proposed EOA and other algorithms without considering power losses for 140-unit system

Figures 6 and 7 compares the convergence curves recorded by running the proposed EOA, GWO, DEA, and PSO without considering power losses for cases 1–3 for 80-unit system and for cases 6–8 for 140-unit system, respectively. The proposed EOA reaches the optimal solution with a minimum number of iterations compared with other methods.

**Fig. 8:**
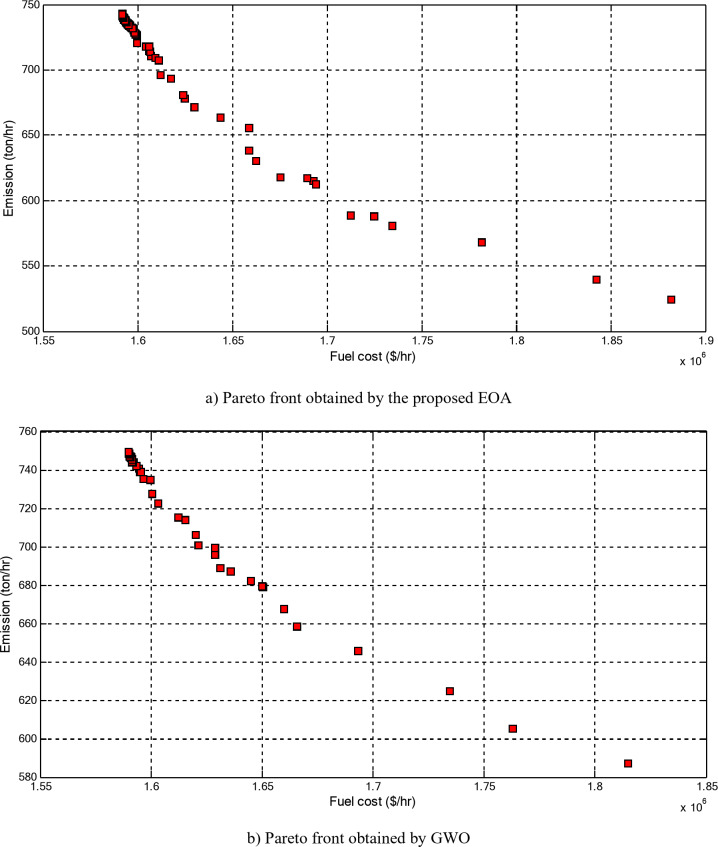

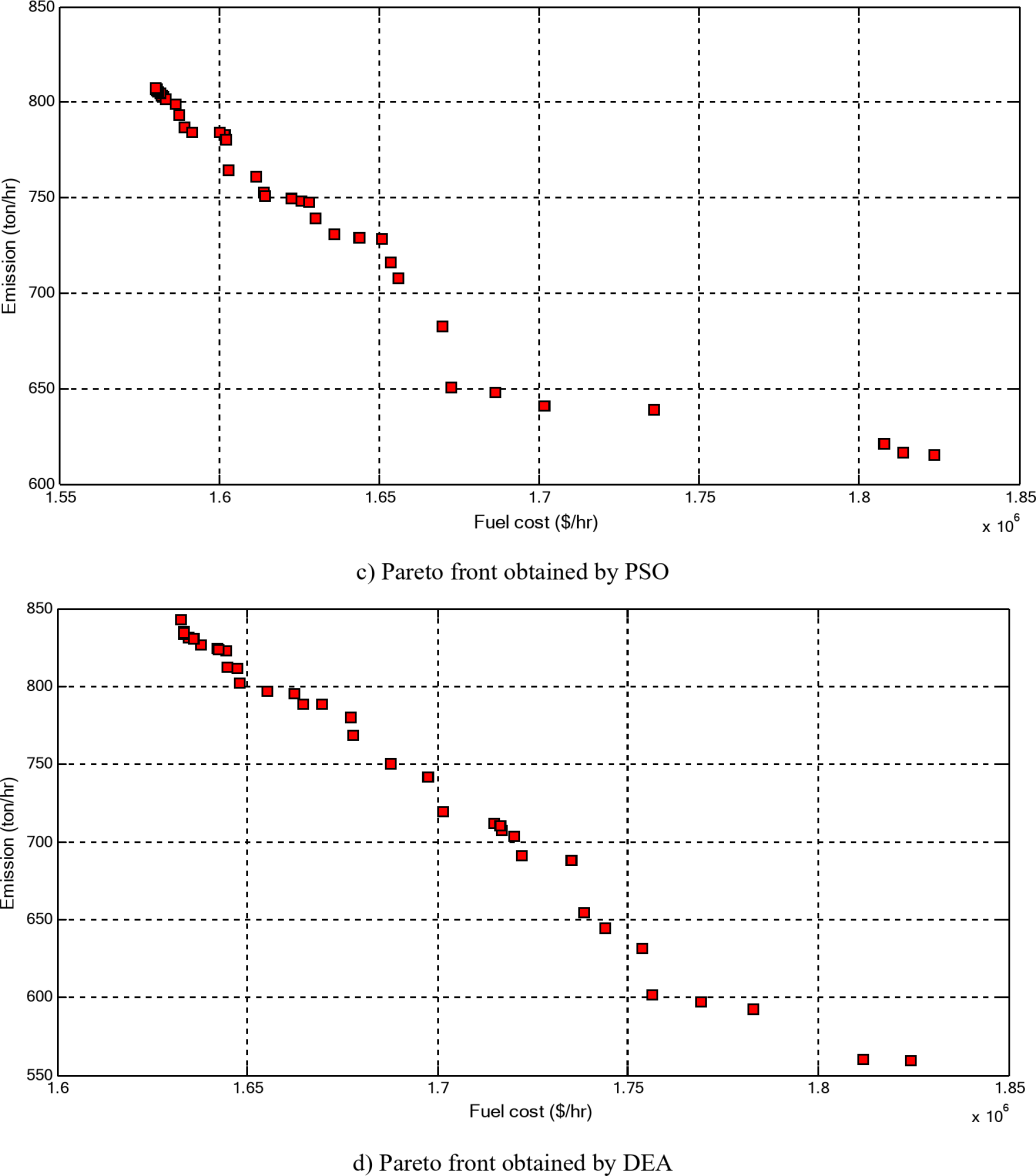
Pareto front obtained by the proposed EOA, GWO, PSO, and DEA for 140-unit system (Case 9).

Table 23 shows the multi-objective optimization results obtained using the proposed EOA and other methods to minimize the total fuel cost and total emission without considering VPE, RRL, and POZs for 140-unit system. In addition, the Pareto front is obtained to find the best compromise between the total fuel cost and emission. The proposed EOA gives better results than other methods by finding the best compromise solution for reducing the total fuel cost and total emission. Figure 8 shows the results of the Pareto front obtained by the proposed EOA and other methods for 140-unit system. From Table 23; Fig. 8, the optimal values of total fuel cost and emission using the proposed EOA are 1.6063 × 10^6^ $/hr and 714.4009 ton/hr, respectively. Therefore, this comparison reflects the superiority of the proposed EOA with the Pareto front for minimizing total cost and emission as a multi-OF.

**Table 23:** Simulation results of multi-OF using different algorithms with Pareto front without considering VPE, RRL, and POZs for 140-unit system (Case 9).

Unit (MW)	DEA	PSO	GWO	Proposed EOA	Unit (MW)	DEA	PSO	GWO	Proposed EOA	Unit (MW)	DEA	PSO	GWO	Proposed EOA
P_G1_	71.000	114.692	106.355	73.448	P_G51_	504.000	247.138	358.211	299.235	P_G101_	958.000	953.158	943.252	957.932
P_G2_	134.787	150.508	164.770	120.172	P_G52_	178.609	165.000	244.171	165.010	P_G102_	890.885	1007.000	1006.901	1005.884
P_G3_	126.981	125.058	167.456	158.794	P_G53_	184.377	167.559	284.364	184.039	P_G103_	1006.000	1005.954	965.038	1005.514
P_G4_	165.797	163.419	152.409	174.447	P_G54_	348.963	167.439	165.450	190.253	P_G104_	1013.000	1013.000	1001.728	1013.000
P_G5_	190.000	99.864	115.580	90.298	P_G55_	180.000	184.963	188.973	180.071	P_G105_	1020.000	1009.961	1017.866	1019.372
P_G6_	90.000	190.000	103.206	90.645	P_G56_	180.000	180.000	184.252	192.785	P_G106_	954.000	954.000	954.000	954.000
P_G7_	461.581	490.000	461.624	483.194	P_G57_	103.000	103.000	119.009	188.984	P_G107_	952.000	840.221	940.427	951.610
P_G8_	490.000	490.000	467.641	490.000	P_G58_	227.811	311.360	198.118	220.362	P_G108_	822.692	834.629	908.657	803.799
P_G9_	496.000	490.124	451.376	495.695	P_G59_	300.350	204.091	312.000	302.892	P_G109_	795.000	795.000	801.010	983.926
P_G10_	495.494	496.000	488.444	496.000	P_G60_	334.940	305.674	173.103	394.398	P_G110_	978.661	1018.566	996.950	1001.793
P_G11_	339.640	496.000	495.606	494.798	P_G61_	500.000	173.614	317.778	213.079	P_G111_	1015.000	1001.318	1003.491	1014.656
P_G12_	482.982	477.786	496.000	488.898	P_G62_	269.880	302.000	95.000	183.670	P_G112_	146.934	95.856	201.027	94.001
P_G13_	506.000	506.000	504.849	506.000	P_G63_	350.122	173.314	486.493	511.000	P_G113_	200.736	203.000	155.889	94.329
P_G14_	496.194	509.000	491.401	506.557	P_G64_	160.000	193.377	198.619	207.020	P_G114_	94.000	94.000	94.000	94.727
P_G15_	506.000	506.000	493.977	505.926	P_G65_	349.894	420.460	477.232	223.690	P_G115_	377.707	248.790	260.479	246.423
P_G16_	316.727	502.983	499.237	496.909	P_G66_	428.262	490.000	196.000	198.807	P_G116_	379.000	244.000	379.000	244.000
P_G17_	506.000	506.000	501.215	505.854	P_G67_	245.285	490.000	220.406	198.671	P_G117_	244.000	249.053	285.633	259.858
P_G18_	506.000	480.202	462.153	503.558	P_G68_	196.000	256.085	309.029	198.073	P_G118_	121.166	105.729	168.220	104.351
P_G19_	450.024	457.083	479.529	504.903	P_G69_	130.000	169.078	130.000	146.471	P_G119_	95.000	95.000	129.559	128.390
P_G20_	505.000	260.615	447.055	501.109	P_G70_	403.283	130.000	330.013	355.098	P_G120_	194.000	124.212	127.934	116.000
P_G21_	274.872	503.689	502.038	498.622	P_G71_	164.094	171.070	246.223	143.845	P_G121_	192.078	183.698	176.032	189.047
P_G22_	365.942	505.000	448.553	503.787	P_G72_	420.605	137.000	334.966	167.343	P_G122_	8.919	2.000	16.444	2.576
P_G23_	505.000	505.000	474.592	485.144	P_G73_	271.592	369.769	195.215	535.769	P_G123_	17.016	37.895	4.000	6.031
P_G24_	505.000	504.953	485.436	504.480	P_G74_	416.010	457.478	406.429	240.624	P_G124_	15.000	22.930	81.810	15.522
P_G25_	518.685	537.000	529.367	536.461	P_G75_	540.000	265.507	175.116	213.030	P_G125_	9.000	22.010	12.702	44.498
P_G26_	537.000	536.142	537.000	523.188	P_G76_	267.695	245.175	424.386	175.096	P_G126_	16.233	22.974	12.772	12.442
P_G27_	549.000	493.382	510.537	543.373	P_G77_	540.000	403.279	175.299	438.916	P_G127_	20.501	34.000	25.551	15.262
P_G28_	359.544	498.650	434.332	546.286	P_G78_	573.224	330.000	358.175	338.457	P_G128_	112.000	112.213	127.371	112.078
P_G29_	291.850	478.401	501.000	486.201	P_G79_	494.915	509.993	531.000	529.258	P_G129_	4.000	8.698	5.482	5.186
P_G30_	501.000	501.000	480.134	499.212	P_G80_	456.229	439.077	531.000	527.757	P_G130_	5.000	11.790	6.412	5.148
P_G31_	506.000	484.596	500.513	504.827	P_G81_	200.000	289.130	200.000	235.868	P_G131_	18.020	5.328	18.870	8.212
P_G32_	486.749	468.010	506.000	505.392	P_G82_	56.007	120.313	57.643	56.012	P_G132_	98.000	84.284	62.255	55.246
P_G33_	506.000	501.846	501.023	505.760	P_G83_	161.264	195.737	115.465	115.000	P_G133_	10.000	5.005	10.000	8.595
P_G34_	473.744	433.156	496.545	503.766	P_G84_	143.804	143.478	162.512	129.825	P_G134_	49.596	42.226	42.165	48.491
P_G35_	397.116	489.899	483.579	498.795	P_G85_	160.401	153.099	118.055	138.778	P_G135_	72.369	46.765	42.507	49.125
P_G36_	365.189	497.432	500.000	499.818	P_G86_	245.323	221.532	231.254	219.486	P_G136_	44.762	41.000	55.502	41.899
P_G37_	172.348	185.074	241.000	241.000	P_G87_	207.000	210.044	217.846	207.010	P_G137_	17.000	18.419	43.128	17.000
P_G38_	133.554	216.937	240.798	234.604	P_G88_	182.476	188.021	175.000	344.090	P_G138_	8.717	7.000	11.285	7.000
P_G39_	772.298	772.599	759.725	774.000	P_G89_	175.000	345.000	344.980	279.457	P_G139_	16.029	8.151	11.699	18.403
P_G40_	766.836	768.730	758.670	768.281	P_G90_	175.000	345.000	175.000	175.000	P_G140_	33.490	30.195	26.160	29.270
P_G41_	19.000	4.057	4.956	7.655	P_G91_	345.000	210.645	175.152	175.117	**Total PG**	49,342	49,342	49,342	49,342
P_G42_	3.000	15.458	3.460	21.564	P_G92_	533.145	580.000	579.692	571.723					
P_G43_	160.000	204.646	211.441	160.023	P_G93_	569.635	637.698	645.000	641.941	**Cost ($/hr)**	**1.7563 × 10** ^**6**^	**1.6725 × 10** ^**6**^	**1.6599 × 10** ^**6**^	**1.6063 × 10** ^**6**^
P_G44_	250.000	200.873	162.449	233.537	P_G94_	795.000	983.832	984.000	982.987	**Emission (ton/hr)**	**601.7356**	**650.3219**	**667.6225**	**714.4009**
P_G45_	229.338	245.191	163.225	160.037	P_G95_	978.000	933.538	952.907	973.525					
P_G46_	215.709	226.852	175.274	160.000	P_G96_	657.069	651.717	583.231	682.000					
P_G47_	171.392	214.257	246.208	160.500	P_G97_	684.806	720.000	720.000	717.126					
P_G48_	250.000	228.784	191.578	218.873	P_G99_	718.000	707.731	711.956	718.000					
P_G49_	165.375	249.703	182.989	248.477	P_G99_	670.763	707.305	697.704	720.000					
P_G50_	202.564	250.000	250.000	232.965	P_G100_	949.345	964.000	963.061	956.922					

**Fig. 9:**
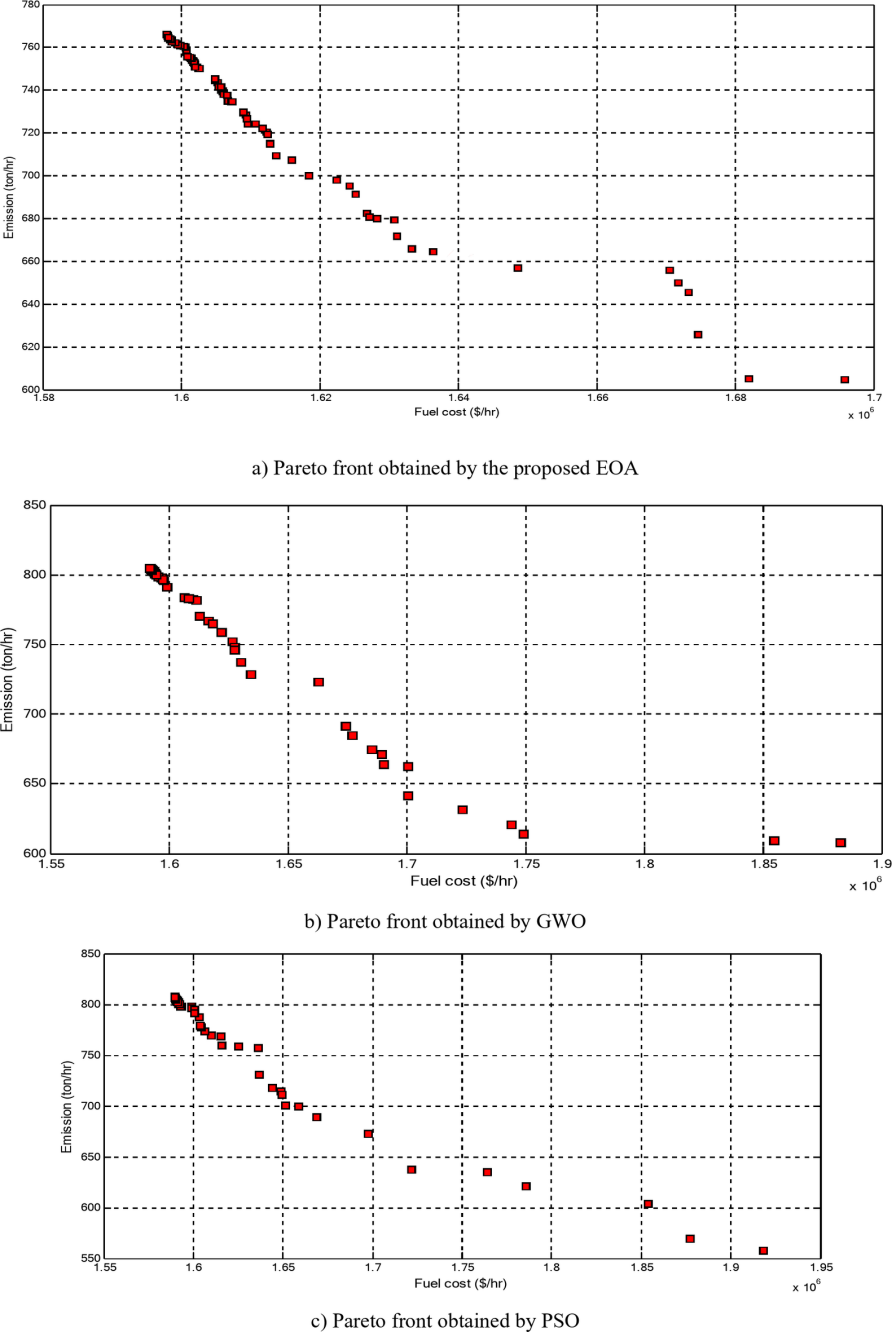

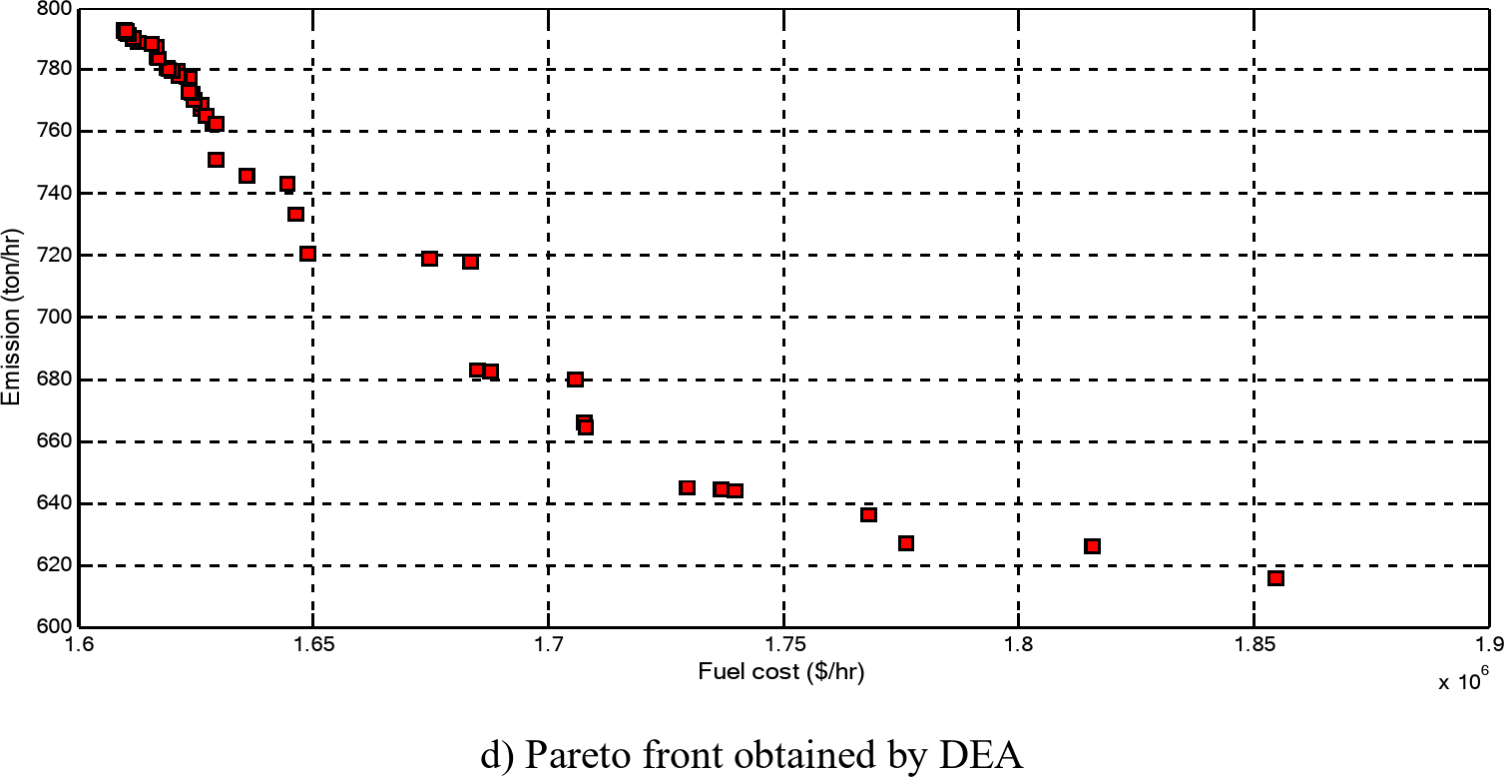
Pareto front obtained by the proposed EOA, GWO, PSO, and DEA for 140-unit system (Case 10).

Table 24 shows the multi-objective optimization results obtained using the proposed EOA and other methods to minimize the total fuel cost and emission considering VPE, RRL, and POZs for 140-unit system. Moreover, the Pareto front is obtained to find the best compromise between the total fuel cost and emission. The best compromise solution between the total fuel cost and total emission is obtained using the proposed EOA. Figure 9 shows the results of the Pareto front obtained by the proposed EOA and other methods for the 140-unit system. From Table 24; Fig. 9, the optimal values of total fuel cost and emission using the proposed EOA are 1.6417 × 10^6^ $/hr and 663.6264 ton/hr, respectively. Therefore, this comparison reflects the great capability of the proposed EOA with the Pareto front for minimizing total fuel cost and emission as a multi-OF.

**Table 24:** Simulation results of multi-OF using different algorithms with Pareto front considering VPE, RRL, and POZs for 140-unit system (Case 10).

Unit (MW)	DEA	PSO	GWO	Proposed EOA	Unit (MW)	DEA	PSO	GWO	Proposed EOA	Unit (MW)	DEA	PSO	GWO	Proposed EOA
P_G1_	76.749	102.401	71.218	78.789	P_G51_	166.617	188.046	165.000	301.705	P_G101_	847.274	958.000	958.000	957.244
P_G2_	189.000	120.000	130.123	187.500	P_G52_	504.000	460.435	165.000	171.496	P_G102_	905.698	994.151	1007.000	1002.642
P_G3_	169.374	179.173	188.092	189.545	P_G53_	165.000	349.706	504.000	168.166	P_G103_	929.059	1006.000	1004.272	985.473
P_G4_	171.115	127.807	125.000	186.323	P_G54_	165.000	369.035	195.087	169.669	P_G104_	1013.000	976.929	1007.888	974.904
P_G5_	183.874	90.771	90.000	90.674	P_G55_	206.240	186.741	227.480	232.250	P_G105_	1011.966	1020.000	1015.224	1019.989
P_G6_	129.748	134.173	160.067	91.734	P_G56_	274.837	180.000	282.910	260.354	P_G106_	951.169	934.042	940.920	954.000
P_G7_	485.839	438.214	490.000	490.000	P_G57_	232.736	253.190	289.215	207.807	P_G107_	805.214	786.000	952.000	864.375
P_G8_	484.081	452.841	477.067	489.273	P_G58_	219.363	221.452	252.909	281.695	P_G108_	814.922	857.235	795.000	886.408
P_G9_	480.288	260.000	495.651	472.803	P_G59_	183.941	138.382	100.991	100.000	P_G109_	1009.704	795.000	795.000	830.345
P_G10_	408.846	496.000	489.984	494.787	P_G60_	153.000	311.440	452.638	442.613	P_G110_	980.577	873.021	972.839	990.697
P_G11_	496.000	484.270	496.000	492.591	P_G61_	441.857	163.000	167.386	178.459	P_G111_	1015.000	1010.617	1000.093	1013.241
P_G12_	478.152	376.249	470.573	495.879	P_G62_	95.000	138.743	114.862	294.457	P_G112_	95.037	94.000	158.991	94.000
P_G13_	436.984	506.000	506.000	500.954	P_G63_	436.661	511.000	161.898	164.720	P_G113_	200.382	203.000	133.673	94.000
P_G14_	483.200	506.411	509.000	508.798	P_G64_	160.017	160.932	496.241	179.137	P_G114_	97.560	103.475	110.301	94.000
P_G15_	464.569	506.000	506.000	505.253	P_G65_	445.265	296.181	366.033	210.017	P_G115_	244.000	314.591	276.266	363.289
P_G16_	494.931	505.000	495.826	505.000	P_G66_	309.956	196.000	490.000	428.121	P_G116_	311.030	261.431	245.155	245.852
P_G17_	503.813	505.724	506.000	504.432	P_G67_	199.004	310.995	486.898	202.942	P_G117_	379.000	244.000	244.000	316.479
P_G18_	271.274	506.000	477.915	505.935	P_G68_	346.184	490.000	241.611	196.000	P_G118_	95.604	166.504	97.442	97.601
P_G19_	483.839	505.000	505.000	431.099	P_G69_	152.727	130.000	178.431	142.068	P_G119_	178.210	96.046	189.000	97.571
P_G20_	495.806	489.271	490.395	486.437	P_G70_	301.252	263.501	431.785	139.343	P_G120_	158.434	119.315	135.497	120.209
P_G21_	439.895	500.915	460.665	501.094	P_G71_	446.800	274.700	137.319	352.567	P_G121_	250.676	179.235	203.168	260.972
P_G22_	412.769	505.000	439.876	497.335	P_G72_	137.000	137.000	137.076	137.400	P_G122_	2.000	2.000	6.216	2.490
P_G23_	505.000	444.874	505.000	505.000	P_G73_	541.000	229.004	273.377	203.285	P_G123_	31.340	45.071	58.920	6.887
P_G24_	424.382	505.000	287.138	504.149	P_G74_	535.393	536.000	536.000	187.561	P_G124_	81.199	83.000	39.502	18.264
P_G25_	465.286	537.000	403.012	526.561	P_G75_	267.558	540.000	196.195	180.664	P_G125_	32.156	53.000	16.045	27.130
P_G26_	530.541	509.950	522.922	527.758	P_G76_	175.421	350.386	284.242	409.826	P_G126_	37.000	14.378	31.261	12.969
P_G27_	386.045	549.000	503.823	547.910	P_G77_	175.000	175.000	175.000	175.523	P_G127_	10.000	31.278	10.000	10.263
P_G28_	441.113	520.472	370.188	548.314	P_G78_	402.686	330.000	330.436	330.000	P_G128_	269.768	112.000	112.806	117.487
P_G29_	379.574	371.060	418.708	496.176	P_G79_	465.837	531.000	505.869	518.728	P_G129_	16.213	17.001	4.000	5.442
P_G30_	501.000	467.054	501.000	463.157	P_G80_	531.000	322.669	525.545	530.963	P_G130_	8.338	20.230	9.004	5.000
P_G31_	495.431	503.883	506.000	506.000	P_G81_	542.000	200.000	278.087	542.000	P_G131_	6.980	11.728	5.000	18.952
P_G32_	495.427	388.010	501.238	501.851	P_G82_	132.000	128.223	56.000	121.370	P_G132_	52.220	50.306	87.652	71.006
P_G33_	506.000	506.000	506.000	506.000	P_G83_	236.748	245.000	120.969	115.000	P_G133_	5.000	5.857	5.000	8.682
P_G34_	495.052	498.883	488.440	491.905	P_G84_	239.809	126.734	125.734	115.000	P_G134_	74.000	44.628	65.879	42.531
P_G35_	260.000	499.986	500.000	499.198	P_G85_	115.000	133.629	138.716	116.998	P_G135_	44.981	44.982	74.000	43.257
P_G36_	260.000	500.000	463.828	499.996	P_G86_	262.734	236.383	212.804	207.000	P_G136_	41.931	78.832	48.631	41.777
P_G37_	241.000	241.000	164.559	241.000	P_G87_	207.292	225.468	250.522	211.248	P_G137_	51.000	17.000	34.131	17.000
P_G38_	120.251	241.000	194.458	239.077	P_G88_	317.321	212.378	345.000	175.359	P_G138_	19.000	16.114	11.835	17.974
P_G39_	773.315	773.698	683.198	774.000	P_G89_	197.002	175.000	306.442	281.450	P_G139_	7.016	14.455	7.556	7.001
P_G40_	751.665	766.230	769.000	765.634	P_G90_	177.452	345.000	295.768	341.060	P_G140_	26.381	31.670	32.837	39.811
P_G41_	6.946	4.827	7.881	3.068	P_G91_	345.000	189.948	175.000	344.782	Total PG	49,342	49,342	49,342	49,342
P_G42_	20.248	25.438	8.577	3.057	P_G92_	579.897	580.000	580.000	573.693					
P_G43_	238.054	246.402	178.749	166.752	P_G93_	632.909	631.553	638.925	644.933	**Cost ($/hr)**	**1.7761 × 10** ^**6**^	**1.7219 × 10** ^**6**^	**1.7008 × 10** ^**6**^	**1.6417 × 10** ^**6**^
P_G44_	241.149	249.715	250.000	205.859	P_G94_	984.000	984.000	984.000	984.000	**Emission (ton/hr)**	**627.1013**	**637.2430**	**641.6806**	**663.6264**
P_G45_	161.868	244.779	160.000	249.407	P_G95_	967.175	858.781	978.000	978.000					
P_G46_	160.000	175.965	174.068	175.938	P_G96_	636.674	633.445	666.686	655.514					
P_G47_	160.000	250.000	161.374	246.677	P_G97_	713.927	720.000	677.525	720.000					
P_G48_	177.044	250.000	179.640	226.540	P_G99_	672.176	655.837	718.000	613.333					
P_G49_	180.731	202.730	160.905	250.000	P_G99_	672.659	720.000	719.645	712.728					
P_G50_	182.570	175.782	199.948	168.211	P_G100_	964.000	964.000	950.632	959.222					

Table 25 presents a comparison between the proposed EOA and other methods for minimizing the total fuel cost and emission simultaneously with and without VPE, RRL, and POZs for 140-unit system. The proposed EOA gives better results than other methods by finding the best compromise solution for reducing the total fuel cost and total emission. This comparison reflects the superiority of the proposed EOA for solving the multi-OF for large-scale power systems.

**Table 25:** Comparison between the multi-OF using the proposed EOA and other methods for 140-unit system (Cases 9, 10).

Method	Case 9		Case 10	
	Fuel cost	Emission	Fuel cost	Emission
Proposed EOA	1.6063 × 10^6^	714.4009		
GWO	1.6599 × 10^6^	667.6225	1.7008 × 10^6^	641.6806
PSO	1.6725 × 10^6^	650.3219	1.7219 × 10^6^	637.2430
DEA	1.7563 × 10^6^	601.7356		
MOMSA^22^	1.6491 × 10^6^	49625.757 lb/hr	N/A	N/A

#### Results of statistical analysis

Comparison based on the statistical analysis between the proposed EOA and other algorithms such as GWO, PSO, and DEA has been carried out to evaluate the capability of the proposed EOA to reduce the total fuel cost and emission. Table 26 shows the statistical summary by finding the best, mean, worst, and standard deviation (SD) for each single OF after 50 random trials with the same parameters for test systems. Better performance is obtained using the proposed EOA because of the convergence to the best solution in most trials. In addition, the lower values of SDs based on the proposed EOA are great evidence for this convergence. This comparison reflects the superiority and robustness of the proposed EOA to reach either optimum value or very near to it in every trial for small, medium, and large-scale systems.

**Table 26:** Results of statistical analysis after 50 random trials for test systems.

Test system		Case#	Method	Best	Worst	Average	Standard deviation
**10-Unit**	Without losses	Case 1	Proposed EOA	1.0596 × 10^5^	1.0605 × 10^5^	1.0599 × 10^5^	2.59379 × 10^−4^
			GWO	1.0603 × 10^5^	1.0631 × 10^5^	1.0617 × 10^5^	8.08531 × 10^−4^
			DEA	1.0626 × 10^5^	1.0657 × 10^5^	1.0644 × 10^5^	9.16892 × 10^−4^
			PSO	1.0611 × 10^5^	1.0639 × 10^5^	1.0626 × 10^5^	8.55446 × 10^−4^
			PHOA [49]	1.0621 × 10^5^	1.0621 × 10^5^	1.0621 × 10^5^	1.7822 × 10^−11^
		Case 2	Proposed EOA	1.0617 × 10^5^	1.0631 × 10^5^	1.0623 × 10^5^	3.83231 × 10^−4^
			GWO	1.0619 × 10^5^	1.0645 × 10^5^	1.0632 × 10^5^	7.76101 × 10^−4^
			DEA	1.0634 × 10^5^	1.0667 × 10^5^	1.0651 × 10^5^	9.42759 × 10^−4^
			PSO	1.0625 × 10^5^	1.0653 × 10^5^	1.0640 × 10^5^	7.41848 × 10^−4^
		Case 3	Proposed EOA	91.9695	91.9706	91.9698	3.05773 × 10^−4^
			GWO	91.9960	92.2431	92.1179	0.0678991
			DEA	93.8757	94.3543	94.1196	0.1584541
			PSO	93.6060	93.9584	93.7747	0.1053699
**20-Unit**	Without losses	Case 1	Proposed EOA	6.0156 × 10^4^	6.0171 × 10^4^	6.0161 × 10^4^	4.60128 × 10^−4^
			GWO	6.0193 × 10^4^	6.0246 × 10^4^	6.0219 × 10^4^	0.0016301
			DEA	6.0216 × 10^4^	6.0261 × 10^4^	6.0239 × 10^4^	0.0013085
			PSO	6.0234 × 10^4^	6.0281 × 10^4^	6.0257 × 10^4^	0.0013543
	Considering losses	Case 1	Proposed EOA	6.2136 × 10^4^	6.2151 × 10^4^	6.2141 × 10^4^	4.74109 × 10^−4^
			GWO	6.2271 × 10^4^	6.2308 × 10^4^	6.2291 × 10^4^	0.0011424
			DEA	6.2311 × 10^4^	6.2364 × 10^4^	6.2336 × 10^4^	0.0015726
			PSO	6.2294 × 10^4^	6.2357 × 10^4^	6.2327 × 10^4^	0.0018873
			BSA^48^	6.24566 × 10^4^	6.2458 × 10^4^	6.2457 × 10^4^	NA
**40-Unit**	Without losses	Case 1	Proposed EOA	1.1865 × 10^5^	1.1897 × 10^5^	1.1876 × 10^5^	9.91527 × 10^−4^
			GWO	1.1914 × 10^5^	1.2186 × 10^5^	1.2039 × 10^5^	0.0084653
			DEA	1.1998 × 10^5^	1.3054 × 10^5^	1.2427 × 10^5^	0.0300562
			PSO	1.1947 × 10^5^	1.2843 × 10^5^	1.2431 × 10^5^	0.0261402
			EMFO^48^	1.2039 × 10^5^	1.2049 × 10^5^	1.2045 × 10^5^	4.02
		Case 2	Proposed EOA	1.21408 × 10^5^	1.2213 × 10^5^	1.2169 × 10^5^	0.0013968
			GWO	1.2344 × 10^5^	1.2947 × 10^5^	1.2651 × 10^5^	0.0165992
			DEA	1.2468 × 10^5^	1.3582 × 10^5^	1.2937 × 10^5^	0.0337135
			PSO	1.2502 × 10^5^	1.3746 × 10^5^	1.3154 × 10^5^	0.0378627
		Case 3	Proposed EOA	0.66599 × 10^5^	0.66657 × 10^5^	0.6662 × 10^5^	1.72744 × 10^−4^
			GWO	0.72978 × 10^5^	0.73816 × 10^5^	0.7351 × 10^5^	0.0026167
			DEA	0.82389 × 10^5^	0.88519 × 10^5^	0.8563 × 10^5^	0.0180595
			PSO	0.76434 × 10^5^	0.82243 × 10^5^	0.7944 × 10^5^	0.0183292
**80-Unit**	Without losses	Case1	Proposed EOA	2.3732 × 10^5^	2.3779 × 10^5^	2.3748 × 10^5^	0.0013572
			GWO	2.3943 × 10^5^	2.4108 × 10^5^	2.4032 × 10^5^	0.0050623
			DEA	2.4540 × 10^5^	2.5347 × 10^5^	2.4945 × 10^5^	0.0243934
			PSO	2.5205 × 10^5^	2.6079 × 10^5^	2.5712 × 10^5^	0.0274695
		Case 2	Proposed EOA	2.4584 × 10^5^	2.4651 × 10^5^	2.4608 × 10^5^	0.0021214
			GWO	2.5132 × 10^5^	2.5387 × 10^5^	2.5278 × 10^5^	0.0074468
			DEA	2.6017 × 10^5^	2.6714 × 10^5^	2.6395 × 10^5^	0.0203537
			PSO	2.5507 × 10^5^	2.6108 × 10^5^	2.5807 × 10^5^	0.0180165
			EMFO^48^	2.4290 × 10^5a^	2.4325 × 10^5^	2.4303 × 10^5^	51.651
		Case 3	Proposed EOA	1.3335 × 10^5^	1.3378 × 10^5^	1.3342 × 10^5^	0.0012851
			GWO	1.3957 × 10^5^	1.4216 × 10^5^	1.4119 × 10^5^	0.0070898
			DEA	1.5562 × 10^5^	1.5682 × 10^5^	1.5637 × 10^5^	0.0037674
			PSO	1.4431 × 10^5^	1.4521 × 10^5^	1.4489 × 10^5^	0.0023845
**140-Unit**	Without losses	Case 6	Proposed EOA	1.5625 × 10^6^	1.5683 × 10^6^	1.5649 × 10^6^	0.0017213
			GWO	1.6384 × 10^6^	1.6817 × 10^6^	1.6653 × 10^6^	0.0062714
			DEA	1.6823 × 10^6^	1.7531 × 10^6^	1.6291 × 10^6^	0.0075812
			PSO	1.6697 × 10^6^	1.8143 × 10^6^	1.7512 × 10^6^	0.0089176
		Case 7	Proposed EOA	1.6196 × 10^6^	1.6318 × 10^6^	1.6237 × 10^6^	0.0025041
			GWO	1.6871 × 10^6^	1.7235 × 10^6^	1.7109 × 10^6^	0.0083151
			DEA	1.7544 × 10^6^	1.8565 × 10^6^	1.8211 × 10^6^	0.0107253
			PSO	1.7460 × 10^6^	1.8392 × 10^6^	1.8153 × 10^6^	0.0098374
		Case 8	Proposed EOA	457.0131	457.2624	457.1532	0.0013832
			GWO	464.9570	466.1691	465.7225	0.0051985
			DEA	468.3059	469.7316	469.4208	0.0074829
			PSO	469.0625	470.6139	469.8957	0.0089137

NA: Not available.

^a^ The exact value of total fuel cost is 2.5558 × 10^5^ $/hr, which is higher than that reported in^48^.

#### Results of nonparametric statistical analysis

Comparison based on the Wilcoxon signed-rank test between the proposed EOA and other algorithms such as GWO, PSO, and DEA has been carried out to compare the OF values from each run with a 5% significance threshold. Table 27 shows the results of the p-value obtained for test systems for the Wilcoxon signed-rank test using the proposed EOA and other methods after 50 random trials with the same parameters and iterations for test systems. It can be observed that all the p-values are less than 0.05 for all cases, indicating that the proposed EOA is significantly different than other methods. This test reflects the superiority of the proposed EOA for finding better solutions than other methods when solving the EELD problem.

**Table 27:** Results of Wilcoxon signed-rank test after 50 random trials for test systems.

Test system	Case#	Algorithms	*P*-value
10-unit	1	EOA vs. GWO	2.2414 × 10^−8^
		EOA vs. PSO	7.4613 × 10^−10^
		EOA vs. DEA	5.2661 × 10^−12^
	2	EOA vs. GWO	2.1605 × 10^−8^
		EOA vs. PSO	1.3418 × 10^−11^
		EOA vs. DEA	7.9688 × 10^−12^
	3	EOA vs. GWO	3.2752 × 10^−7^
		EOA vs. PSO	8.2913 × 10^−11^
		EOA vs. DEA	7.9657 × 10^−13^
20-unit	1 (Without losses)	EOA vs. GWO	4.2852 × 10^−9^
		EOA vs. PSO	7.5362 × 10^−11^
		EOA vs. DEA	7.3041 × 10^−11^
	1 (Considering losses)	EOA vs. GWO	5.1467 × 10^−10^
		EOA vs. PSO	6.7854 × 10^−12^
		EOA vs. DEA	7.2139 × 10^−13^
40-unit	1	EOA vs. GWO	5.5853 × 10^−8^
		EOA vs. PSO	7.3178 × 10^−12^
		EOA vs. DEA	6.8052 × 10^−13^
	2	EOA vs. GWO	3.8192 × 10^−9^
		EOA vs. PSO	7.1207 × 10^−12^
		EOA vs. DEA	8.3514 × 10^−12^
	3	EOA vs. GWO	5.0501 × 10^−7^
		EOA vs. PSO	4.1287 × 10^−11^
		EOA vs. DEA	6.3514 × 10^−12^
80-unit	1	EOA vs. GWO	5.5853 × 10^−11^
		EOA vs. PSO	7.3178 × 10^−13^
		EOA vs. DEA	6.8052 × 10^−14^
	2	EOA vs. GWO	3.7521 × 10^−10^
		EOA vs. PSO	5.6274 × 10^−13^
		EOA vs. DEA	6.7359 × 10^−14^
	3	EOA vs. GWO	4.8546 × 10^−9^
		EOA vs. PSO	6.3587 × 10^−11^
		EOA vs. DEA	7.1085 × 10^−11^
140-unit	1	EOA vs. GWO	8.1672 × 10^−10^
		EOA vs. PSO	6.5931 × 10^−14^
		EOA vs. DEA	5.8916 × 10^−13^
	2	EOA vs. GWO	5.7213 × 10^−9^
		EOA vs. PSO	7.1158 × 10^−13^
		EOA vs. DEA	9.0734 × 10^−12^
	3	EOA vs. GWO	6.5193 × 10^−9^
		EOA vs. PSO	5.8573 × 10^−11^
		EOA vs. DEA	7.1842 × 10^−12^

## Conclusions

This paper proposed an efficient procedure based on the EOA for an economical/environmental operation of power systems by solving the EELD problem considering single and multi-objective functions. Two OFs have been considered by minimizing the total fuel cost and emission with and without considering practical constraints such as VPE, RRL, POZs, and transmission system losses. In addition, the multi-OF, which aims to minimize these objectives simultaneously, has been considered. The proposed EOA has been evaluated and tested on small, medium, and large-scale test systems having 10, 20, 40, 80, and 140 units. The numerical results have been compared with the results using other optimization techniques such as GWO, PSO, DEA, and other optimization techniques in the literature. Also, the proposed EOA has been evaluated and compared with other optimization techniques based on statistical analysis and statistical checks-based Wilcoxon-score rank test for solving the EELD problem considering different OFs. These comparisons proved the superiority of the proposed EOA for solving the EELD problem with more accuracy and efficiency. According to the numerical results and comparisons between the proposed EOA and other methods for different studied cases, it can be concluded that,

- For 10-unit test system, the total fuel cost without and with considering VPE obtained using the proposed EOA is reduced by 0.1414%, and 0.0753% than the base case (results of PSO) with maximum savings of 150 $/hr, and 80 $/hr, respectively. The total emission is reduced by 1.7483% than the base case. For multi-OF, the total fuel cost without and with considering VPE is reduced with maximum savings of 70 $/hr, and 1700 $/hr than the base case, while the total emission is reduced by 4.4067%, and 1.7456% than the base case.
- For 20-unit test system, the total fuel cost without and with considering system losses is reduced by 0.1295%, and 0.2536% than the base case (results of PSO) with maximum savings of 78 $/hr and 158 $/hr.
- For 40-unit test system, the total fuel cost without and with considering VPE is reduced by 0.6864%, and 2.8891% than the base case (results of PSO) with maximum savings of 820 $/hr and 3610 $/hr, respectively. The total emission is reduced by 12.8673% than the base case. For multi-OF, the total fuel cost without and with considering VPE is reduced with maximum savings of 1230 $/hr, and 4960 $/hr than the base case, while the total emission is reduced by 10.7355%, and 9.2553% than the base case.
- For 80-unit test system, the total fuel cost without and with considering VPE is reduced by 5.8441% and 3.6186% than the base case (results of PSO) with maximum savings of 14,730 $/hr and 9230 $/hr, respectively. The total emission is reduced by 7.5948% than the base case. For multi-OF, the total fuel cost without and with considering VPE is reduced with maximum savings of 18,470 $/hr and 15,270 $/hr than the base case, while the total emission is reduced by 15.4641%, and 14.2918% to the base case.
- For 140-unit test system as a large-scale power system, the total fuel cost without and with considering VPE, RRL, and POZs is reduced by 6.4203%, and 7.2394% with maximum savings of 107,200 $/hr and 126,400 $/hr than the base case (results of PSO), respectively. The total emission is reduced by 2.5688% than the base case. For multi-OF with Pareto optimal front, the proposed EOA gives the best compromise between the considered OFs.
- The comparison based on the statistical analysis between the proposed EOA, and other optimization techniques proved the superiority of the proposed EOA for solving the EELD problem.
- The application of non-parametric tests by the Wilcoxon signed-rank test on the results of the proposed EOA explains the reliability of the proposed algorithm.

In future work, we plan to solve the EELD problem, considering the integration of renewable energy sources (RES) and plug-in electric vehicles (PEVs) in microgrid (MG).

## Data Availability

All data generated or analyzed during this study are included in this published article.
